# Thermodynamically programmed one-pot CRISPR platform for point-of-care SNP genotyping

**DOI:** 10.1038/s41467-026-75358-1

**Published:** 2026-07-07

**Authors:** Xiaolong Wu, Yanan Li, Yumeng Cao, Zibin Zhao, Hongyu Lu, Shaochong Liang, Grace C. Y. Lui, Denise P. C. Chan, I-Ming Hsing

**Affiliations:** 1 Department of Chemical and Biological Engineering, The Hong Kong University of Science and Technology, Kowloon, Hong Kong China; 2 Department of Medicine and Therapeutics, The Chinese University of Hong Kong, Shatin, Hong Kong China; 3 S.H. Ho Research Centre for Infectious Diseases, The Chinese University of Hong Kong, Shatin, Hong Kong China

**Keywords:** Assay systems, CRISPR-Cas systems, Nucleic acids, Biosensors

## Abstract

One-pot CRISPR diagnostics face a fundamental incompatibility: isothermal nucleic acid amplification enables rapid target accumulation, whereas CRISPR activation irreversibly consumes those substrates, destabilizing reaction kinetics. Here we show that reaction order can be programmed into DNA primers through thermodynamic design. Differences in primer-binding strength create two sequential amplification stages, delaying CRISPR activation until enough amplicons have accumulated without physical separation or external control. The design also introduces the protospacer adjacent motif (PAM), a short sequence required for CRISPR recognition, through the primer rather than relying on its presence in the native target, expanding target accessibility while retaining single-nucleotide discrimination. An ordinary differential equation model captures the threshold behavior and establishes a predictable framework for primer design. Building on this principle, we develop Thermodynamically Encoded Molecular Programming for One-pot diagnostics (TEMPO), which achieves attomolar sensitivity within 30 min and enables sequencing-concordant SNP genotyping and pathogen detection in a single-step microfluidic format.

## Abstract (web summary)

One-pot CRISPR diagnostics are limited by kinetic conflict between isothermal amplification and CRISPR detection. By thermodynamically programming reaction order into DNA primers, the authors create staged amplification enabling rapid, sensitive, single-step nucleic acid testing.

## Introduction

Fully integrated molecular diagnostics aim to combine nucleic acid amplification, molecular recognition and signal transduction within a single reaction environment^1^. Achieving such integration, however, requires multiple biochemical processes to occur in a defined temporal order despite sharing the same reactor and molecular intermediates^2^. When reaction order is externally imposed, through physical separation, staged operation or manual intervention, robust performance can be maintained^3–5^. In contrast, when competing reactions proceed simultaneously without intrinsic ordering, system-level instability emerges, undermining sensitivity, reproducibility and predictability^6^. Designing molecular systems that autonomously enforce reaction order therefore represents a central but unresolved challenge in integrated diagnostics.

This challenge is particularly acute in one-pot CRISPR-based diagnostics^2,7,8^. CRISPR–Cas12a has enabled highly sensitive nucleic acid detection by coupling sequence-specific target recognition to collateral nuclease activity^9,10^. When combined with isothermal amplification, such systems promise rapid and portable diagnostics^11–13^. However, amplification and CRISPR activation form a tightly coupled reaction network that competes for the same target molecules^14^. Amplification requires sustained accumulation of target DNA, whereas CRISPR activation irreversibly consumes those targets through *cis*- and *trans*-cleavage. In a one-pot format, this intrinsic conflict destabilizes reaction kinetics, leading to premature target depletion, suppressed amplification and reduced sensitivity^8,14,15^. These limitations are amplified in applications requiring high specificity, such as single-nucleotide polymorphism (SNP) genotyping, where both stringent sequence discrimination and stable reaction kinetics are required^16–18^. Many clinically relevant SNPs underpin pathogen drug resistance, cancer heterogeneity and pharmacogenomic decision-making, yet their reliable detection remains difficult in one-pot CRISPR systems^16,17,19^. In this context, minor kinetic instability or premature target consumption can obscure single-nucleotide differences, rendering high-resolution genotyping unreliable.

Existing strategies to address this interference have focused on mitigating, rather than resolving, the underlying lack of reaction order. Approaches such as empirical tuning of reagent concentrations^20,21^, weakening protospacer adjacent motif (PAM) recognition^22^, engineering crRNA^23,24^ or primer^25,26^, or introducing physical or temporal separation^27,28^ can partially alleviate competition between amplification and CRISPR activity. However, these solutions rely on external control or context-specific parameter balancing and therefore cannot guarantee robust performance across different targets, sequence contexts or operating conditions. Moreover, PAM dependence fundamentally constrains genomic addressability: fewer than a minority of clinically relevant SNP loci are proximal to canonical Cas12a PAM sequences^29,30^, rendering most variants inaccessible to conventional CRISPR diagnostics. As a result, current one-pot CRISPR systems face a persistent trade-off between workflow simplicity, target scope and single-nucleotide specificity.

Here we propose that reaction order in one-pot CRISPR diagnostics can be molecularly programmed rather than externally imposed, enabling autonomous and predictable coordination of amplification and CRISPR transduction within a single reactor. We introduce thermodynamic encoding as a design principle in which reaction sequencing is embedded directly into DNA primer energetics. By engineering a defined free-energy asymmetry between competing amplification primers, the system autonomously evolves through kinetically ordered amplification regimes, prioritizing target accumulation before activating CRISPR-mediated signal transduction. This intrinsic, threshold-gated coordination decouples amplification from CRISPR activity within a single, unsegmented reactor, without the need for physical separation or external triggers. Importantly, this architecture relocates PAM dependence from native genomic targets to primer-encoded design, enabling access to loci that are otherwise undetectable while preserving single-nucleotide discrimination.

Building on this principle, we develop Thermodynamically Encoded Molecular Programming for One-pot diagnostics (TEMPO), a programmable CRISPR platform that integrates isothermal amplification, PAM design and kinetic control into a single molecular framework. Using a quantitative ordinary differential equation model to guide primer design, TEMPO achieves predictable reaction ordering, attomolar sensitivity and robust SNP discrimination in a fully integrated one-pot format. We validate this approach using HIV RNA mock samples, Influenza A and SARS-CoV-2 clinical samples, and clinically relevant human SNPs lacking proximal PAM sites and further demonstrate its applicability through integration into a single-step microfluidic diagnostic chip. Together, these results establish thermodynamic encoding as a generalizable design paradigm for coordinating competing biochemical processes within shared reactors, with implications extending beyond CRISPR diagnostics to integrated molecular systems that require autonomous reaction ordering.

## Results

### Thermodynamically encoded reaction ordering enables autonomous one-pot CRISPR coordination

Previous efforts have attempted to temporally separate amplification and CRISPR activation using external gating elements such as photocaged crRNAs^23^ or temperature switch^31^. While effective, these systems lack an intrinsic mechanism to prioritize amplification over CRISPR activation, resulting in uncontrolled competition for shared target molecules. Under such conditions, premature CRISPR engagement inevitably suppresses amplification efficiency and destabilizes signal generation. We reasoned that enforcing reaction order requires not parameter tuning or weakened CRISPR activity, but the introduction of an explicit energetic hierarchy between competing amplification pathways, such that reaction sequencing could occur as an intrinsic consequence of molecular thermodynamics.

**Fig. 1:**
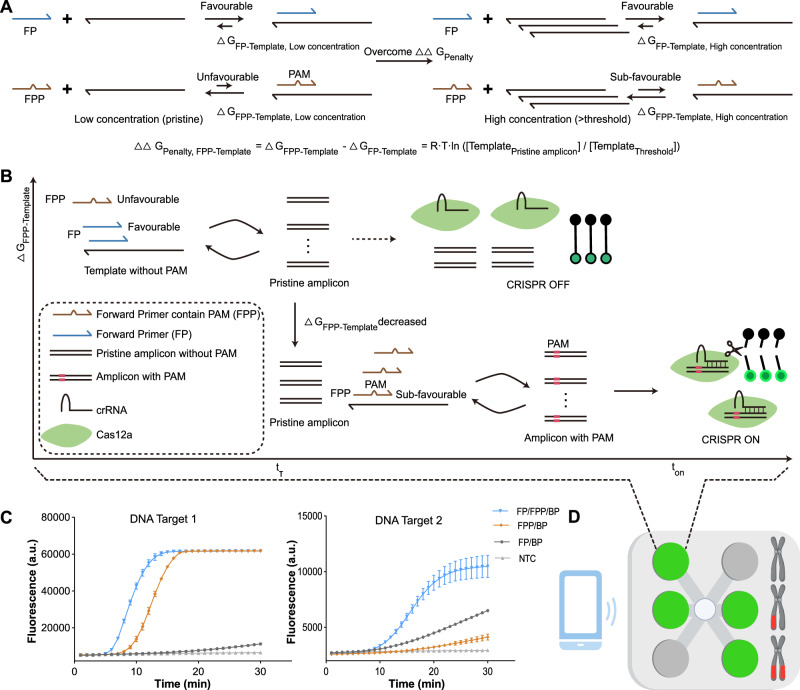
Thermodynamically encoded reaction ordering enables autonomous one-pot CRISPR coordination. Thermodynamic design of two competing primer-binding pathways. A fully complementary forward primer (FP) readily hybridizes to the target and drives rapid recombinase polymerase amplification (RPA), producing pristine amplicons. In contrast, the PAM-containing primer carries mismatches (FPP), creating an unfavorable hybridization energy and preventing early engagement. Numbers of mismatches in FPP can be designed via ΔΔG_penalty, FPP-Template_ = RT ln ([Template _Pristine amplicons_] / [Template _Threshold_]). **B** Conceptual free-energy landscape illustrating chemical potential-driven pathway activation in the TEMPO reactor. Pristine RPA carrying FP primer initiates immediately along a low-barrier pathway due to favorable primer–template hybridization, producing PAM-free amplicons that accumulate without engaging CRISPR. The PAM-introducing amplification branch is initially inaccessible because an intentional mismatch imposes a higher free-energy barrier. As pristine amplicons accumulate, the increasing chemical potential of the template pool provides a concentration-dependent driving force that progressively lowers the effective barrier, enabling PAM-containing amplification. The resulting mixed amplicon population activates Cas12a via PAM-dependent *cis*-cleavage, followed by *trans*-cleavage of the reporter for signal generation. **C** TEMPO validation using DNA target 1 (derived from HIV genome, 1 fM) and DNA target 2 (derived from Human genome, 1 fM). *n* = 3 technical replicates; error bars represent the mean ± SD. **D** Schematic of on-site TEMPO detection using a single-step microfluidic chip. Following sample loading, the one-pot amplification–CRISPR reaction proceeds autonomously, and fluorescence signals are captured and interpreted by a smartphone-based readout. All fluorescence intensity is reported in arbitrary units (a.u.). Source data are provided as a Source Data file. (Created in BioRender. Hsing, I. (2026) https://BioRender.com/a8fgwuo).

To achieve this, we introduced two forward primers with a defined thermodynamic asymmetry (Fig. 1A, Supplementary Fig. 1): a fully complementary primer (FP, ΔG_FP-Template_ ≈ −37.20 kcal/mol) that drives efficient amplification of PAM-free amplicons, and a deliberately mismatched primer (FPP, ΔG_FPP-Template_ ≈ −30.71 kcal/mol) that encodes a PAM but incurs a higher hybridization free-energy penalty. This energetic imbalance necessarily partitions amplification into two kinetically distinct regimes. At low template abundance, only the high-affinity FP pathway is thermodynamically accessible, leading to rapid accumulation of pristine amplicons that cannot activate Cas12a (Fig. 1B). As amplification proceeds and template concentration increases, according to the thermodynamic relationship ΔG_eff_ = ΔG° − RT ln([Template]) + C where C is a system-specific constant, the effective free-energy barrier for FPP binding is progressively reduced, enabling a second amplification regime that installs PAM-containing amplicons. CRISPR activation therefore acts as a threshold-gated behavior rather than an externally triggered event.

Our results reveal that a defined free-energy difference between the two forward primers gives rise to two kinetically ordered phases of isothermal recombinase polymerase amplification (RPA). RPA reactions using the primer pairs FP/BP or FPP/BP (where BP is the backward primer) each produced a single amplicon of the expected size, whereas reactions containing both forward primers generated two distinct products corresponding to the pristine and FPP-extended amplicons (Supplementary Fig. 2A). Sequencing data verified that the FPP primer introduces the engineered PAM without affecting the original product (Supplementary Fig. 2B). Real-time kinetics further indicated that the higher-affinity FP primer drives the early phase of amplification, while the FPP primer, penalized by reduced binding strength, engages more slowly. At high template input, the rate difference narrows, consistent with mass-action effects overcoming the thermodynamic penalty (Supplementary Fig. 3**)**. Additional assays verified that only PAM-containing dsDNA efficiently activates Cas12a (Supplementary Fig. 4).

Consistent with this mechanism, reactions containing only FP produced robust amplification without detectable CRISPR signal, whereas reactions driven solely by FPP exhibited weak and unstable fluorescence due to simultaneous amplification and target consumption. In contrast, reactions containing both primers reproducibly displayed delayed but strong CRISPR activation, reflecting autonomous reaction ordering encoded by primer thermodynamics rather than empirical parameter balancing (Fig. 1C). Importantly, ongoing FP-driven amplification continuously replenished templates, preventing target depletion even after CRISPR activation and yielding stable one-pot kinetics. Furthermore, the system remained largely insensitive to potential Mg²⁺ fluctuations during this dynamic process, with highly similar kinetics observed across 19–21 mM Mg²⁺ and only a modest reduction in reaction performance at 15 mM (Supplementary Fig. 5). Based on this principle, we establish Thermodynamically Encoded Molecular Programming for One-pot diagnostics (TEMPO) and integrate it into a microfluidic platform (Fig. 1D) for rapid pathogen detection and SNP analysis.

### Quantitative modeling establishes reaction ordering as a predictable design outcome

To conceptualize this behavior and distinguish intrinsic reaction ordering from empirical tuning, we constructed a quantitative reaction-network model describing primer competition, amplification and PAM-dependent Cas12a activation (Supplementary Note 1). The model comprises coupled ordinary differential equations that encode primer hybridization kinetics, strand extension and CRISPR-mediated cleavage as mass-action and Michaelis–Menten processes.

#### Primer competition and thermodynamic gating

We modeled primer binding as bimolecular association processes. Central to the engineered thermodynamic switch is the mismatch-bearing primer FPP, whose reduced hybridization free energy is encoded directly into slower kinetic rate constants:

1 $$\frac{d[{{{\rm{FPP}}}}\cdot {{{\rm{D}}}}]}{{dt}}={k}_{6}\left[{{{\rm{FPP}}}}\right]\left[{{{\rm{D}}}}\right]-{k}_{6}^{{\mbox{ext}}}\left[{{{\rm{FPP}}}}\cdot {{{\rm{D}}}}\right]$$

in contrast to fully complementary FP:

2 $$\frac{d[{{{\rm{FP}}}}\cdot {{{\rm{D}}}}]}{{dt}}={k}_{1}[{{{\rm{FP}}}}][{{{\rm{D}}}}]-{k}_{1}^{{\mbox{ext}}}[{{{\rm{FP}}}}\cdot {{{\rm{D}}}}]$$

with *k*_6_ < *k*_1_ capturing the thermodynamic penalty imposed by the designed mismatch. This asymmetry produces two kinetically distinct amplification channels: a high-affinity pristine pathway leading to the native amplicon *D*, and a delayed mismatch pathway that generates the PAM-containing amplicon *D*_2_.

#### Sequential amplification and PAM emergence

The reactor evolves according to mass-action coupling between strand-displacement extension and template regeneration:

3 $$\frac{d[D]}{{dt}}={k}_{3}[{{{\rm{BP}}}}][{{{\rm{ST}}}}]+{k}_{4}[{{{\rm{FP}}}}][{{{\rm{AT}}}}]+{k}_{5}[{{{\rm{ST}}}}][{{{\rm{AT}}}}]-{k}_{13}[{{{\rm{C}}}}][D]$$

4 $$\frac{d[{D}_{2}]}{{dt}}={k}_{8}[{{{\rm{BP}}}}][{{{{\rm{ST}}}}}_{2}]+{k}_{11}[{{{\rm{FPP}}}}][{{{{\rm{AT}}}}}_{2}]+{k}_{12}[{{{{\rm{ST}}}}}_{2}][{{{{\rm{AT}}}}}_{2}]-{k}_{14}[{{{\rm{C}}}}][{D}_{2}]$$

where the emergence of *D*_2_ is controlled by the mismatch-driven delay in FPP-mediated extension. These expressions quantitatively reveal that PAM introduction is not an external trigger but a threshold phenomenon emergent from the kinetic imbalance between the two amplification tracks.

#### PAM-dependent CRISPR transduction as a catalytic nonlinear activation

Cas12a activation is represented using two parallel Michaelis–Menten channels with dramatically different catalytic efficiencies:

5 $${v}_{1}=\frac{{k}_{{{{\rm{cat}}}}1}[{{{\rm{CD}}}}][R]}{{K}_{m1}+[R]},{v}_{2}=\frac{{k}_{{{{\rm{cat}}}}2}[{{{{\rm{CD}}}}}_{2}][R]}{{K}_{m2}+[R]}$$

with ${k}_{{{{\rm{cat}}}},2}\gg {k}_{{{{\rm{cat}}}},1}$. This structure creates an activation nonlinearity normally seen in engineered chemical reactors where a slow path primes the system for abrupt activation once a specific intermediate (here, *D*_2_) reaches a critical concentration.

#### Model-experiment agreement validates a predictable sequential reactor

In this framework, the mismatch-bearing FPP primer is represented by a reduced effective association rate constant relative to FP, directly reflecting its higher hybridization free-energy. This asymmetry necessarily generates two amplification pathways with distinct kinetic accessibility. Model simulations predict three defining features of thermodynamically encoded reaction ordering: (i) suppression of CRISPR activation during early exponential amplification, (ii) delayed emergence of PAM-containing amplicons once a threshold template concentration is reached, and (iii) a sharp, nonlinear onset of *trans*-cleavage activity following PAM installation.

Experimental kinetics closely matched these predictions across multiple targets and primer designs (Fig. 1C, Supplementary Fig. 6). The observed delay in CRISPR activation, its threshold-like onset, and the sustained amplification after activation all emerged without invoking external triggers or time-dependent parameter changes. Notably, both the model and the experimental data suggest that Cas12a *trans*-cleavage activity may also cleave primers, thereby introducing a negative-feedback effect. Although this feedback slightly reduced amplicon yield, it had minimal impact on signal-generation kinetics or endpoint output in the one-pot reaction (Supplementary Fig. 7). Importantly, altering primer free-energy penalties or PAM identity shifted the predicted threshold and activation kinetics in a manner quantitatively consistent with experimental observations, confirming that primer thermodynamics act as a tunable control variable rather than an empirical adjustment.

### Programmable DNA engineering operates as a multi-axis kinetic control element

Building on the model, we next examined whether primer engineering could systematically shape reaction dynamics. Conventional RPA–CRISPR-Cas12a coupling, primarily regulates reaction kinetics by modifying crRNA–target interactions^32–34^. RPA primers are seldom altered, as sequence changes frequently compromise amplification efficiency. In contrast, in TEMPO the amplification efficiency is primarily contributed by the pristine primer FP, while FPP serves as a molecular bridge linking RPA output to Cas12a activation. This architecture makes FPP an unusually flexible design element: because it is not the dominant driver of bulk amplification, its sequence can be engineered not only at the PAM-encoding segment, but also across multiple positions along the primer, including regions that overlap with the downstream crRNA recognition sequence. As a result, FPP programming can simultaneously tune both RPA progression and CRISPR kinetics, providing a level of multidimensional control not accessible in conventional one-pot designs.

We infer that FPP exposes three orthogonal axes of design flexibility: (i) Varying FPP length modulated hybridization free-energy and predictably shifted the timing of PAM-containing amplification, thereby tuning the delay between amplification and CRISPR activation. (ii) Independently, altering the encoded PAM sequence modulated Cas12a activation strength without substantially affecting amplification kinetics, decoupling signal transduction from target generation. (iii) Finally, modifying the FPP–crRNA overlap region effectively introduced programmable mismatches at the recognition interface, providing an additional layer of kinetic control without rescreening crRNAs.

**Fig. 2:**
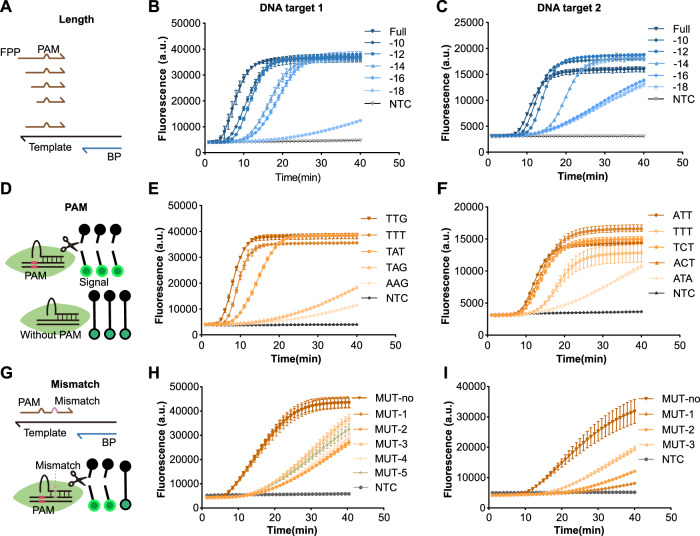
Programmability of FPP primers via hybridization strength and PAM engineering. **A** Schematic illustrating modulation of primer–template hybridization thermodynamics by varying the length of the FPP primer, thereby tuning the rate of PAM-containing secondary amplification. Primer length serves as a programmable handle to adjust −ΔG_FPP-Template_. All reactions were performed with a fixed template input of 1 fM. **B**, **C** Kinetic profiles of TEMPO reactions programmed by FPP primer length. “Full” denotes the full-length FPP primer, and “−n” indicates truncation by n nucleotides. Panels **B** and **C** correspond to DNA targets derived from the HIV and human genomes, respectively. *n* = 3 technical replicates; error bars represent the mean ± SD. **D** Schematic illustrating FPP primers encoding different PAM sequences modulate CRISPR signal output. **E**, **F** Kinetic profiles of TEMPO reactions programmed with different PAM variants. Panel **E** shows reactions evaluated using DNA target 1 derived from the HIV genome (PAMs: TTG, TTT, TAT, TAG, and AAG), whereas panel F shows reactions evaluated using DNA target 2 derived from the human genome (PAMs: TTT, ATT, TCT, ACT, and ATA). *n* = 3 technical replicates; error bars represent the mean ± SD. **G** Schematic of crRNA-overlap engineering, where FPP primer modification introduces an effective mismatch at the CRISPR recognition locus. **H**, **I** Kinetic profiles of TEMPO reactions programmed with effective mismatches at defined positions downstream of the PAM. MUT-n indicates a single-nucleotide mismatch introduced n bases after the PAM via FPP–crRNA overlap engineering. Panel H and panel I correspond to DNA targets derived from the HIV and human genomes, respectively. *n* = 3 technical replicates; error bars represent the mean ± SD. All fluorescence intensity is reported in arbitrary units (a.u.). Source data are provided as a Source Data file.

Firstly, we systematically adjusted FPP length while maintaining mismatch position constant (Fig. 2A). Shorter FPP variants, with weaker binding affinity, slowed and reduced PAM-containing product formation, extending the temporal separation between amplification and Cas12a activation (Fig. 2B, C). Conversely, increasing FPP length lowered the thermodynamic penalty, advancing the onset of CRISPR activation. These results indicate that mismatch-encoded ΔG operates as a kinetic valve, determining when the secondary amplification branch begins contributing to reactor output.

We next tuned Cas12a activation independently of amplification by varying the PAM triplet within FPP (Fig. 2D, F). PAM identity modulated Cas12a activation strength without substantially affecting RPA kinetics. Canonical PAMs (TTT) induced rapid Cas12a activation driven by efficient *cis*-cleavage, whereas sub-optimal PAMs (TTG, ATT) produced more gradual and sustained activation kinetics. This behavior likely arises because weaker PAMs mediate reduced Cas12a cleavage activity, thereby alleviating competition between CRISPR cleavage and PAM-containing amplification. In contrast, excessively weak PAMs resulted in minimal signal output, consistent with their intrinsically low Cas12a activation efficiency.

Finally, inspired by prior studies showing that crRNA mismatches modulate Cas12a kinetics^34^, but usually require extensive crRNA screening, we hypothesized that mutating the FPP–crRNA overlap region would indirectly introduce mismatch into the crRNA recognition interface, providing an alternative regulatory handle. Indeed, kinetic profiles confirmed this effect (Fig. 2G-I).

Across these perturbations, reaction trajectories followed the same underlying sequence predicted by the model, differing only in timing and amplitude. This consistency demonstrates that thermodynamic encoding transforms primer design into a compact, multi-dimensional control space for shaping one-pot reaction dynamics, rather than a trial-and-error optimization problem.

### DNA engineering enables broad-spectrum and highly specific SNP detection

CRISPR-based diagnostics have been widely applied to SNP genotyping^18,35^. However, practical implementation remains constrained by two longstanding challenges: (i) strict PAM requirements limit accessible loci^9,36^, and (ii) single-nucleotide discrimination is not consistently achieved, particularly in one-pot RPA–CRISPR formats^37,38^. As a result, many clinically important SNPs remain undetectable.

**Fig. 3:**
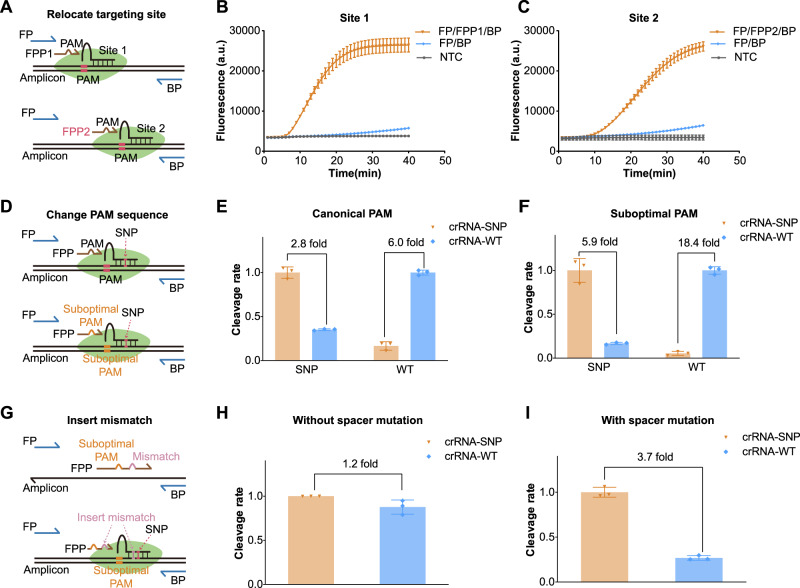
DNA engineering enables broad-spectrum and highly specific SNP detection. **A** Relocation of targeting site via primer engineering. Diagram of the two forward-selective primers (FPP1 and FPP2) positioned at different sites on the target sequence. **B**, **C** Real-time fluorescence amplification curves for the rs199476104 target (1 fM) in the presence or absence of FPP1 and FPP2. The curves (FP/FPP/BP) represent the full TEMPO reaction system, while the curves (FP/BP) show the reaction without FPP enhancement. NTC, non-template control; *n* = 3 technical replicates; error bars represent the mean ± SD. **D** Design strategy for PAM switching via FPP mismatch engineering. Mismatches within the FPP-binding region reassign the PAM sequence, enabling allele-dependent Cas12a activation. **E**, **F** Cas12a cleavage results for canonical PAM (TTT) and suboptimal PAM variants (ATT) using the rs671 target SNP/WT genotype (1 fM input). *n* = 3 technical replicates; Error bars represent the means ± SD. **G** Design strategy using a single-base insertion to enhance allele discrimination. The insertion is introduced downstream of the suboptimal PAM-encoding position within FPP, deliberately shifting the crRNA–target register to create a defined single-base mismatch in the seed region. **H**, **I** Cas12a cleavage results for the rs1229984 target SNP genotype (1 fM input) comparing designs without and with an insertion mutation. *n* = 3 technical replicates; Error bars represent the means ±SD. All fluorescence intensity is reported in arbitrary units (a.u.). Source data are provided as a Source Data file.

Given the programmable kinetic control demonstrated previously in TEMPO, we reasoned that FPP primer engineering could convert arbitrary SNPs, regardless of PAM availability, into high-fidelity, one-pot detectable targets. We first validated that TEMPO could redirect Cas12a targeting solely through FPP design (Fig. 3A-C). Within a fixed RPA amplicon, repositioning the FPP primer selectively introduced PAMs at user-defined locations, enabling Cas12a activation at either of two sites. Additional sequence designs further support the generality of this strategy (Supplementary Fig. 8). Importantly, this targeting flexibility did not compromise sensitivity, as amplification driven by FPP remained intact. This property is particularly critical for SNP genotyping, where PAMs are often absent near the variant.

Previous work has shown that crRNA or PAM engineering can enhance SNP discrimination^32^; here we demonstrate that DNA-level primer engineering achieves comparable, and in some cases superior control. Building on earlier results showing that PAM identity modulates reaction kinetics, we evaluated whether PAM tuning could improve allelic contrast. Systematic substitution of the PAM triplet within FPP (Fig. 3D) yielded tunable genotype separation: canonical PAMs (TTT) produced 2.8–6.0-fold discrimination between SNP and WT alleles (Fig. 3E), whereas suboptimal PAM variants (ATT) further elevated contrast to 5.9–18.4-fold (Fig. 3F). We attribute this enhancement to moderate reductions in Cas12a affinity that amplify thermodynamic discrimination between matched and mismatched dsDNA substrates.

However, certain SNPs remained poorly resolved even with optimized PAM weakening (Fig. 3H). We therefore introduced a second layer of control by programming the FPP–crRNA overlap region, effectively inserting an additional mismatch at the recognition interface (Fig. 3G). This approach mirrors crRNA engineering but eliminates the need for extensive crRNA rescreening. A single additional mismatch increased wild-type/mutant separation up to 3.7-fold (Fig. 3I), converting previously non-resolvable SNPs into clearly distinguishable genotypes. Together, these results show that FPP primer design enables direct control of both CRISPR target accessibility and allele-level discrimination within a single amplification reaction.

In summary, TEMPO expands the CRISPR detection landscape from PAM-constrained to universally addressable SNP sites, while substantially simplifying assay design. DNA primer programming alone is sufficient to convert undetectable SNPs into high-fidelity readouts, positioning TEMPO as a generalizable molecular framework for precision genotyping and sequence-specific diagnostics.

### Rapid pathogen detection via TEMPO

**Fig. 4:**
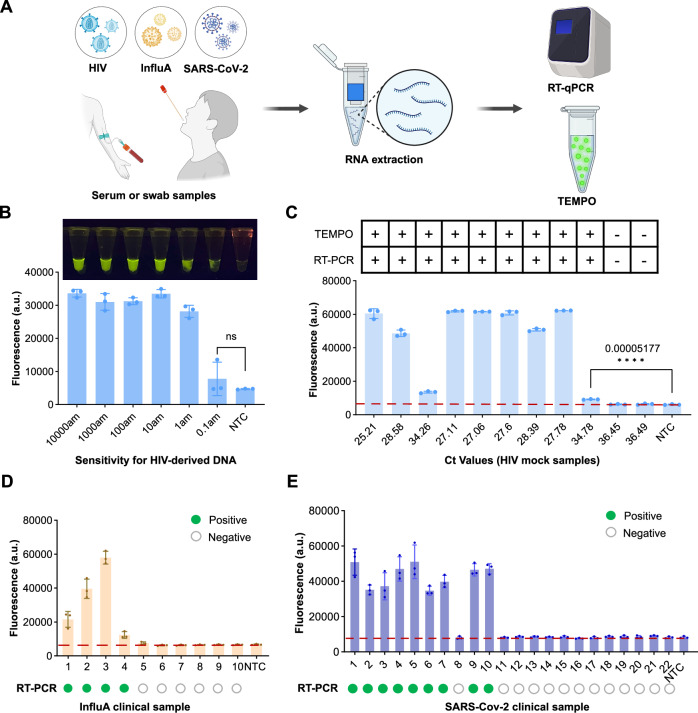
Rapid pathogen detection via TEMPO. **A** Schematic of the streamlined diagnostic workflow for pathogen detection. Clinical specimens (serum or swabs) undergo rapid RNA extraction followed by parallel evaluation via TEMPO and gold-standard RT-qPCR. (Created in BioRender. Hsing, I. (2026) https://BioRender.com/bxoi8iw) **B** Sensitivity of TEMPO for detecting different concentrations of HIV-derived DNA (10,000 aM,1000 aM, 100 aM, 10 aM,1 aM, and 0.1 aM). Strong fluorescence signals were observed down to 1 aM concentration, with clear visual fluorescence under blue light. NTC, non-template control reaction; *n* = 3 technical replicates; error bars represent the mean ± SD. The unpaired two-tailed *t*-test was used to analyze the statistical significance; ns, not significant. **C** Fluorescence intensity of TEMPO platform across extracted HIV RNA mock samples with varying RT-qPCR Ct values. NTC, non-template control reaction; *n* = 3 technical replicates; error bars represent the mean ± SD. The unpaired two-tailed *t*-test was used to analyze the statistical significance (****P < 0.0001). Validation of TEMPO across clinical specimens. Endpoint fluorescence output of TEMPO measured at 30 min for (**D**) 10 Influenza A and (**E**) 22 SARS-CoV-2 clinical samples, with corresponding RT-qPCR results indicated below (green dots: positive; empty circles: negative). The dashed red line represents the threshold for positive detection. For all panels, NTC, non-template control reaction; *n* = 3 technical replicates; error bars represent the means ±SD. All fluorescence intensity is reported in arbitrary units (a.u.). Source data are provided as a Source Data file.

To evaluate the translational potential of TEMPO for rapid pathogen detection, we examined its performance across multiple viral targets and clinically relevant sample types. As illustrated in Fig. 4A, viral RNA from HIV, Influenza A, and SARS-CoV-2, derived from different matrices including serum and respiratory swabs, could be introduced directly into a single-tube TEMPO reaction and benchmarked in parallel against standard RT-qPCR.

Leveraging the programmability of the TEMPO reaction architecture, we first used the established HIV detection system to characterize its analytical performance. The platform exhibited high sensitivity over a broad dynamic range, generating fluorescence signals across input HIV-derived DNA concentrations from 10,000 aM to 1 aM. This response enabled approximate quantitative analysis as well as clear naked-eye discrimination, with a statistically defined analytical limit of detection (LOD) of 1 aM (Fig. 4B, Supplementary Figs. 9, 10). Moreover, the reaction kinetics showed a strong linear correlation with target input (R² = 0.968), supporting a semi-quantitative relationship between signal output and HIV-derived DNA concentration, although the quantitative precision remained below that of standard qPCR (Supplementary Fig. 11). To further assess assay specificity, we challenged the system with four non-target nucleic acid species and observed only background-level fluorescence, supporting the high sequence specificity of the TEMPO platform (Supplementary Fig. 12).

We next evaluated the clinical performance and diagnostic robustness of TEMPO using a diverse set of specimens. In an initial test of 11 HIV RNA mock samples (9 positive and 2 negative), TEMPO results were fully concordant with RT-qPCR (Fig. 4C). This performance was maintained in unpurified crude clinical extracts (Supplementary Fig. 13) and was further extended to additional viral pathogens. Validation of 10 clinical specimens for Influenza A (4 positive and 6 negative) and 22 clinical specimens for SARS-CoV-2 (9 positive and 13 negative) likewise showed agreement with the corresponding RT-qPCR results (Fig. 4D-E, Supplementary Table 1). Notably, clear separation between positive and negative samples was achieved within 30 min under isothermal conditions, representing a substantially shorter assay time than the conventional thermal-cycling qPCR.

Together, these results establish TEMPO as a robust single-tube isothermal platform with sensitivity and sequence selectivity comparable to RT-qPCR across multiple viral targets. Although its quantitative performance does not yet match the precision of RT-qPCR, TEMPO provides reliable semi-quantitative readout together with attomolar-level sensitivity and a simplified workflow. The speed, operational simplicity, and consistent performance across HIV RNA mock samples and clinical samples of Influenza A, and SARS-CoV-2 support its potential as a broadly adaptable framework for decentralized molecular diagnostics and point-of-care pathogen surveillance.

### On-site human genotyping by using TEMPO

Having established the flexibility, extensibility, and allele-level selectivity of TEMPO under controlled benchmark conditions, we next evaluated its performance in human SNP genotyping. We analyzed 20 clinical genomic samples spanning three medically relevant loci, rs1229984, rs671^39^, and rs199476104^40^, each representing distinct challenges in thermodynamic discrimination. For rs671 and rs199476104, single-nucleotide resolution was achieved solely through PAM attenuation, whereas rs1229984 required a combined strategy involving suboptimal PAM insertion and a single engineered mismatch encoded within the FPP primer. This comparative result underscores the modularity of FPP design and demonstrates that TEMPO can be rationally programmed to resolve thermodynamically recalcitrant SNP sites.

**Fig. 5:**
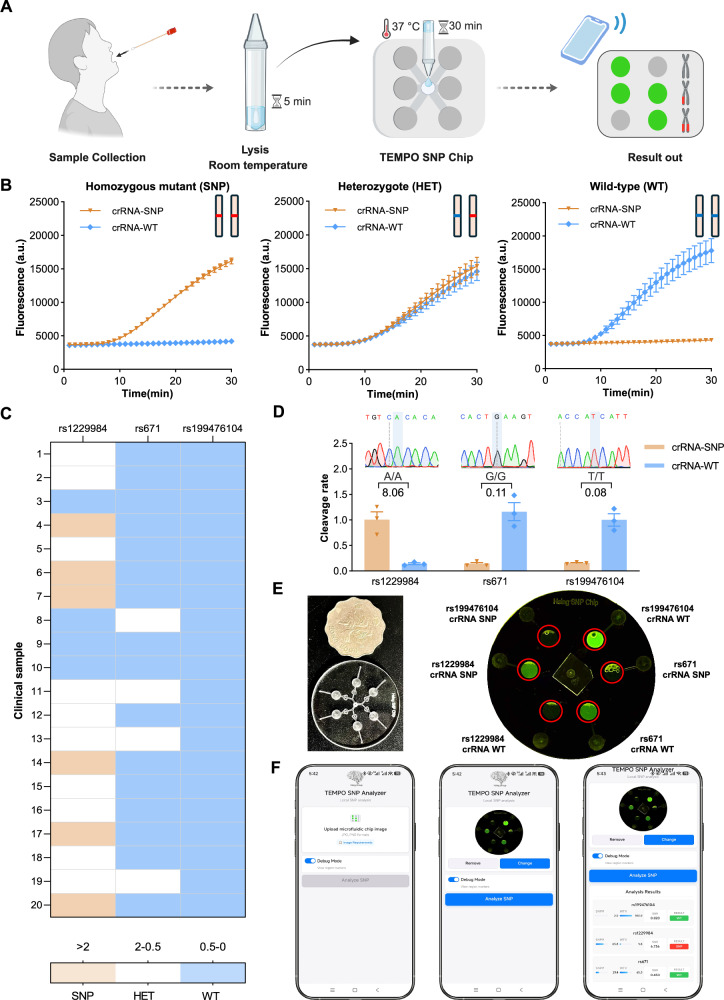
Rapid genotyping of clinically relevant SNPs using TEMPO. **A** Workflow of the SNP genotyping using TEMPO. Swab samples are collected and lysed at room temperature for approximately 5 min. The crude lysate is applied directly to the SNP Chip, where a one-pot RPA–Cas12a reaction is performed at 37 °C for 30 min. Fluorescence signals are then recorded and interpreted using a smartphone interface. (Created in BioRender. Hsing, I. (2026) https://BioRender.com/sq19j81) **B** Representative amplification trajectories illustrating allele-dependent kinetics for rs199476104. Distinct curves corresponding to the wild-type, heterozygous, and mutant genotypes are generated within the one-pot reaction using a 1 fM standard template input. *n* = 3 technical replicates; error bars represent the mean ± SD. **C** Heat map summarizing TEMPO genotyping outcomes for 20 clinical samples across three SNP loci (rs1229984, rs671, and rs199476104). The discrimination factor (DF) encoded in each cell quantitatively captures the relative fluorescence contrast between SNP and wild-type alleles, enabling unambiguous genotype assignment with consistent performance across all samples and loci. Genotype calls were assigned based on DF thresholds, with DF values of 0-0.5 classified as wild type, 0.5–2.0 as heterozygous, and >2 as SNP **D**. Cleavage rate measured from Sample 20 as a representative example. The observed cleavage patterns match the expected allelic state and show strong agreement with Sanger sequencing traces provided above each bar. *n* = 3 technical replicates; error bars represent the mean ± SD. **E** Images of the SNP chip shown with a Hong Kong two-dollar coin for size reference, including the assembled microfluidic chip and representative endpoint fluorescence patterns from sample 20 after a 30 min reaction, highlighting allele-dependent signal differences. **F** Smartphone interface used for real-time data acquisition, visualization and automated SNP reporting. All fluorescence intensity is reported in arbitrary units (a.u.). Source data are provided as a Source Data file.

To translate TEMPO into a clinically viable POC workflow, we developed an integrated platform coupling sample collection, room-temperature lysis, lyophilized one-pot amplification–CRISPR activation, and fluorescence readout into a 35 min sample-to-answer pipeline (Fig. 5A). Buccal-swab lysates were processed by 5 min extraction-free lysis and directly loaded into a palm-sized microfluidic chip (diameter 4 cm, Supplementary Fig. 14) preloaded with freeze-dried TEMPO reagents. Individual reaction chambers can be customized with locus-specific RPA primers and crRNAs. Upon rehydration, reactions self-initiate and produce a quantifiable readout after a 30 min incubation at 37 °C. Fluorescence interpretation is automated through a custom smartphone application, removing the need for manual thresholding or trained operators. This architecture generatively supports on-site genotype determination within 35 min.

We first validated genotyping accuracy using allele-specific crRNAs. TEMPO clearly distinguished wild-type (WT), heterozygous (HET), and homozygous mutant (SNP) states via distinct kinetic trajectories (Fig. 5B), while maintaining attomolar analytical sensitivity (Supplementary Fig. 15). All 20 clinical samples showed full concordance relative to Sanger-validated genotypes across the three loci (Fig. 5C, D**;** Supplementary Data 1): rs1229984 (4 WT/6 SNP/10 HET), rs671 (16 WT/4 HET), and rs199476104 (20 WT). Beyond its robustness under direct-lysis conditions (Supplementary Figs. 16, 17), the system’s utility is further enhanced by a sucrose–mannitol lyophilization matrix. This formulation showed strong stability, retaining near-native enzymatic activity over 4 weeks under refrigerated and frozen storage conditions, 4 °C and −20 °C, respectively. These results support the feasibility of TEMPO for decentralized use while alleviating, although not fully eliminating, dependence on cold-chain handling (Supplementary Figs. 18–20). For on-site SNP genotyping, the validated TEMPO reaction was integrated into a portable microfluidic chip (Supplementary Figs. 14, 21, 22). Distinct chip architectures were developed to support either dual-target or multi-target detection, allowing the number of loci interrogated to be adjusted according to application-specific requirements while preserving a unified one-pot workflow. Clinical sample testing demonstrated clear and reliable signal outputs (Fig. 5E; Supplementary Fig. 23), enabling both direct visual interpretation and automated genotype assignment via a custom smartphone application (Fig. 5F), with minimal user intervention.

Collectively, these results establish TEMPO as a rapid, lyophilization-compatible SNP genotyping platform with programmable resolution down to single-nucleotide variation. By relocating PAM control from the native genome to FPP-encoded design, TEMPO unlocks previously inaccessible loci and greatly simplifies assay re-targeting without extensive crRNA screening. This modularity, coupled with chip integration and mobile analytics, positions TEMPO as a practical foundation for point-of-care precision genotyping and population-scale genetic screening.

## Discussion

Fully integrated one-pot CRISPR diagnostics promise simplified workflows and improved accessibility, yet their practical deployment has been persistently limited by the difficulty of coordinating nucleic acid amplification and CRISPR-mediated signal transduction within a single reaction environment^8^. In most existing systems, these processes compete for shared intermediates, resulting in unstable kinetics, premature target consumption, and inconsistent signal generation^41^. Previous solutions, such as empirical parameter tuning^15^, physical compartmentalization^42^, or externally imposed temporal control^43,44^, have alleviated these conflicts only partially, often at the cost of robustness, generalizability, or operational simplicity. From an engineering perspective, these constraints underscore a fundamental limitation: reaction order in one-pot CRISPR diagnostics is rarely designed, but instead indirectly enforced.

In this work, we introduce thermodynamically encoded reaction ordering as a molecular engineering strategy that represents a conceptual shift in one-pot CRISPR diagnostics. Rather than mitigating interference through weakened interactions or external staging, TEMPO encodes reaction sequencing directly into molecular free-energy landscapes, converting reaction order from an operational challenge into a programmable system property. This framework simultaneously addresses a major barrier to SNP genotyping: dependence on protospacer adjacent motifs. By encoding PAM sequences within primers rather than relying on native targets, TEMPO enables PAM-independent access to arbitrary loci while preserving single-nucleotide discrimination, expanding genomic addressability for clinically relevant variants. At the platform level, TEMPO achieves attomolar sensitivity, single-nucleotide resolution, and sequencing-concordant genotyping across multiple human SNP loci within a fully integrated workflow. Compatibility with extraction-free lysis, lyophilized reagents, and single-step microfluidic implementation further supports deployment in point-of-care and resource-limited settings, achieving high performance without additional reaction steps, specialized hardware, or user intervention. Together, these features establish TEMPO as a programmable, robust, and clinically adaptable framework for one-pot CRISPR diagnostics.

Several limitations define the current scope of the approach. First, while TEMPO supports robust qualitative and semi-quantitative readouts, its quantitative precision does not yet match that of RT-qPCR. This reflects fundamental differences in reaction control: qPCR enforces deterministic amplification stages through thermal cycling^45^, whereas isothermal amplification exhibits greater variability in initiation and growth kinetics^46,47^. In one-pot CRISPR systems, this variability is compounded by dynamic sharing of amplicons between amplification and CRISPR consumption, rendering signal intensity dependent on coupled reaction dynamics rather than template abundance alone. Our ODE model further suggests that nonspecific amplification is an additional contributor to this limitation (Supplementary Fig. 24). Consequently, TEMPO is presently best suited for presence–absence testing and genotyping applications rather than absolute quantification. A second limitation concerns SNP discrimination, which remains inherently dependent on local sequence context. Identical single-nucleotide variants can exhibit divergent discrimination performance depending on neighboring bases and thermodynamic landscape^48–50^. Although multilayer energetic modulation, combining PAM down tuning with engineered mismatches, can enhance allelic resolution, predictive design rules for SNP discrimination remain incomplete, necessitating empirical validation for new targets. Third, while the present study already supports multiplexed analysis through spatial compartmentalization on a microfluidic chip, extending TEMPO to true homogeneous multiplexing within a single reactor remains challenging. In the current chip format, each chamber functions as an isolated one-pot module, enabling six parallel reactions for simultaneous analysis of three SNP loci while avoiding biochemical cross-talk. By contrast, single-reaction multiplexing would require simultaneous control of multiple thermodynamic gates in a shared environment, raising challenges including primer cross-reactivity, competition for shared amplification resources, signal coupling from indiscriminate Cas12a *trans*-cleavage, and overlap between target-specific activation windows. Thus, while TEMPO is already compatible with device-level spatial multiplexing, extending the same principle to true single-pot multiplexing will require further engineering of primer orthogonality, resource balancing, and signal-channel decoupling.

Looking forward, data-driven and physics-informed modeling approaches may help address these challenges^49,51^. Machine-learning frameworks trained on large-scale kinetic and sequence datasets could improve predictive mapping between primer energetics, reaction dynamics, and allelic discrimination performance, thereby enhancing both quantitative accuracy and target generalizability^52,53^. At the same time, advanced algorithmic design tools will likely be required to achieve the thermodynamic orthogonality needed for higher-order multiplexing. More broadly, the principle of thermodynamically encoded reaction ordering is not limited to RPA or Cas12a and may be extended to other amplification chemistries and CRISPR effectors, providing a generalizable strategy for coordinating competing biochemical processes within shared reactors. Integration of TEMPO with digital isothermal amplification architectures may further offer a route toward absolute template quantification, helping overcome its current limitations in quantitative precision. Additionally, while the TEMPO system can be successfully lyophilized, its long-term room-temperature stability requires further optimization for practical clinical applications; future work aims to systematically screen tailored lyoprotectants and refine lyophilization protocols to extend platform shelf-life. Beyond canonical Cas12a, a recently reported DNA-guided RNA-targeting CRISPR-Cas12a system^54^ introduces an additional axis of guide programmability and target scope, which may further broaden the design space for one-pot diagnostics built on the principles described here.

In summary, TEMPO establishes thermodynamic encoding as a practical engineering paradigm for designing predictable, integrated, and deployable one-pot CRISPR diagnostics. By unifying reaction sequencing, PAM independence, and SNP discrimination within a single molecular design framework, this work advances the feasibility of scalable point-of-care and at-home nucleic acid testing, bridging the gap between CRISPR diagnostic concepts and real-world biomedical applications.

## Methods

### Ethical statement

Influenza A and SARS-CoV-2 clinical samples were provided by The Prince of Wales Hospital with approval from the Chinese University of Hong Kong Clinical Research Ethics Committee (CREC-2021-0188). Human genome swab samples used in this study were obtained from volunteers recruited at The Hong Kong University of Science and Technology under a protocol approved by the university’s Human and Artefacts Research Ethics Committee (HREP-2026-0148). All participants provided informed consent prior to sample collection, demonstrating a full understanding of the study’s purpose, procedures, and potential risks. Participants’ personal information will be maintained in strict confidentiality, and all data will be processed anonymously to ensure the protection of their privacy. Samples were obtained by the research team in a deidentified manner, and no demographic information (including sex, age, gender, or other identifiers) was collected or made available to ensure strict participant privacy protection. Participants received no compensation for their participation.

### Materials

*Lachnospiraceae bacterium* Cas12a (LbCas12a) protein was from Tolo Biotech (Shanghai). RNA and DNA oligonucleotides, as well as synthetic DNA templates (Supplementary Data 2), were provided by GENEWIZ (Suzhou, China). Acrylamide/bisacrylamide solution was purchased from Bio-Rad. SYBR Green Nucleic Acid Gel Stain, and SYTO 82 Orange Fluorescent Nucleic Acid Stain were purchased from Thermo Fisher Scientific (Hong Kong). DEPC Treated Water was from Sangon Biotech (Shanghai). DNA Isothermal Rapid Amplification Kit, RNA Isothermal Rapid Amplification Kit and Rapid Nucleic Acid Release Reagent were from Amp-future (Amp-future, Changzhou, China). RNase inhibitor, and NEBuffer 2.1 were from New England Biolabs. Proteinase K, Magnetic Bead Virus DNA/RNA Kit and Magnetic Universal Genomic DNA Kit were from Tiangen (Beijing). One Step RT-qPCR Master Mix and Universal Blue qPCR SYBR Green Master Mix was from YEASEN (Shanghai). Sucrose and mannitol were from Macklin (Shanghai).

### RPA and real-time RPA reactions, gel analysis, and sequencing

RPA reactions were carried out in a 50 µL reaction mixture containing 29.5 µL primer-free rehydration buffer, 1 µL forward primer (10 µM), 1 µL FPP primer (10 µM), 2 µL backward primer (10 µM), 4 µL template, 2.5 µL MgOAc (280 mM), and 1 µL DNase/RNase-free water. Reactions were incubated at 37 °C for 60 min.

To terminate amplification, Proteinase K was added to the completed reaction and incubated at room temperature for 30 min. The reaction was then heat-inactivated at 95 °C for 10 min. The resulting products were either analyzed on 10% denaturing PAGE for gel visualization or submitted for Sanger sequencing (GENEWIZ).

For real-time RPA, the reaction was assembled as described above with the addition of 1 µM SYTO 82 stain, and fluorescence was recorded continuously during incubation at 37 °C.

### TEMPO system

One-pot RPA-Cas12a reactions were assembled in a 20 µL final volume as summarized in Supplementary Table 2. Briefly, 25 nM LbCas12a and 50 nM crRNA were premixed and incubated for 5 min at room temperature to allow formation of the ribonucleoprotein complex. The reaction mixture contained 400 nM reporters, 125 nM each of primer FP and primer FPP, 250 nM primer BP, NEB Buffer 2.1, the rehydration buffer supplied with the RPA kit, and nuclease-free water. 4 µL Template was added to each reaction. MgOAc (280 mM) was added last to initiate amplification, yielding a total reaction volume of 20 µL. All reactions were mixed gently and incubated under standard one-pot amplification conditions. The fluorescent signal (485 nm excitation and 535 nm emission) was monitored with a qPCR system (Bio-Rad) with an interval time of 1 min.

### Sample preparation

HIV RNA mock samples. HIV RNA standards were prepared by spiking 0.5 µL of HIV RNA into 49.5 µL of human serum, followed by nucleic acid extraction using TIANamp Virus DNA/RNA Kit (Tiangen; Beijing; China). The purified RNA was used as the template for the TEMPO one-pot assay as well as RT-qPCR benchmarking.

Influenza A and SARS-CoV-2 raw clinical samples were obtained and inactivated in the Prince of Wales Hospital (Hong Kong). Clinical samples were pretreated for pure RNA collection using the QIAamp Viral RNA Mini Kit (QIAGEN; Hilden; Germany), a fast column extraction kit.

Human genomic samples. Twenty volunteers were recruited at the Hong Kong University of Science and Technology. Buccal swabs were collected from each participant and divided into two aliquots. One aliquot was processed using the Magnetic Universal Genomic DNA Kit to obtain purified genomic DNA, which served as the input for one-pot TEMPO genotyping and for Sanger sequencing after RPA amplification of target. The second aliquot was subjected to rapid lysis using a commercial Rapid Nucleic Acid Release Reagent (Amp-future, Changzhou) and used directly for microfluidic chip–based detection according to the manufacturer’s protocol. Briefly, the swab sample was immersed in a centrifuge tube containing 1 mL of lysis buffer and vortexed thoroughly, followed by incubation at room temperature for 5 min. The tube was briefly centrifuged for 5 s, and 10 μL of the supernatant was collected as the template for direct amplification.

### RT-qPCR validation of HIV RNA mock samples

HIV RNA mock samples were validated using the Hifair Advanced One-Step RT-qPCR SYBR Green Kit (Yeasen). Each 25 μL reaction mixture contained 12.5 μL of 2× Hifair Advanced SYBR Green Buffer, 1 μL of forward primer (10 μM), 1 μL of backward primer (10 μM), 1 μL of Hifair Advanced UH Enzyme Mix, 4 μL of RNA template, and nuclease-free DEPC-treated water to the final volume. RT-qPCR was performed with an initial reverse transcription step at 50 °C for 6 min, followed by pre-denaturation at 95 °C for 5 min. Amplification was then carried out for 40 cycles consisting of denaturation at 95 °C for 15 s and annealing/extension at 60 °C for 30 s.

### Design and fabrication of the microfluidic diagnostic chip

The microfluidic diagnostic chip was designed in SolidWorks 2020 to integrate multiple reaction chambers and fluidic pathways for parallel SNP detection, with chamber volumes defined to accommodate 20–25 μL reactions. Passive Tesla-type valve structures were incorporated to prevent contamination arising from liquid backflow during the reaction process^55^. The chip employed a two-layer architecture, consisting of a CNC-machined polymethyl methacrylate (PMMA) layer containing the microfluidic channels and a non-fluorescent adhesive sealing film (Microseal B, Bio-Rad) as the bottom layer. Vent ports were sealed with a waterproof, breathable expanded polytetrafluoroethylene (ePTFE) membrane (diameter, 3.5 mm) to allow gas exchange while preventing liquid leakage and external contamination. The finalized designs were fabricated from PMMA using CNC micromachining by a commercial microfabrication service provider (Shenzhen Huilixing Precision Manufacturing Co., Ltd., China).

### Reagent loading and lyophilization on chip

The lyophilizable one-pot RPA–Cas12a reaction mixture was prepared according to the formulation summarized in Supplementary Table 3. A 20 μL lyophilizable one-pot RPA–Cas12a reaction mixture was prepared, containing RPA reagents were reconstituted in DEPC-treated water, 125 nM each forward primers FP and FPP, 250 nM backward primer BP, 50 nM Cas12a, 100 nM crRNA, and 800 nM ssDNA reporter, supplemented with 5% (w/w) sucrose and 2% (w/w) mannitol as lyoprotectants. The reaction mixture was dispensed into the reaction chambers (~25 μL per chamber). Chips were snap-frozen by positioning them approximately 1 cm above liquid nitrogen vapor for 2 min, followed by lyophilization for 12 h in the dark. After returning to room temperature, the bottom sealing film and top vent membrane were affixed to complete device assembly.

### Statistics and reproducibility

Statistical analyses were performed using GraphPad Prism 10 and Microsoft Excel 2019. Schematic illustrations were created with BioRender, Adobe Illustrator 2024 and Microsoft PowerPoint 2019. Nucleic acid secondary structures were predicted using NUPACK 4.1. Quantitative data (endpoint fluorescence and time-to-positive measurements) are reported as mean ± SD from 3 technical replicates unless stated otherwise. No statistical method was used to predetermine sample size. No data was excluded from the analysis. The experiments were not randomized for group allocation; samples and templates were assigned to predefined assay conditions, such as target identity, genotype, concentration or control status. Where applicable, the order of sample preparation and fluorescence measurement was randomized to minimize potential handling or run-order effects. The Investigators were not blinded to allocation during experiments and outcome assessment because the primary outcomes were instrument-based quantitative measurements analyzed using predefined criteria.

Fluorescence data were normalized using DF to quantify the relative signal contrast between SNP and wild-type reactions. The DF was calculated as^27^,

$${{{\rm{DF}}}}=({{{{\rm{F}}}}}_{{{{\rm{SNP}}}}}-{{{{\rm{F}}}}}_{{{{\rm{NTC}}}}})/({{{{\rm{F}}}}}_{{{{\rm{WT}}}}}-{{{{\rm{F}}}}}_{{{{\rm{NTC}}}}}),$$

where F_SNP_, F_WT_, and F_NTC_ represent the fluorescence intensities measured for SNP, wild-type, and no-target control reactions, respectively.

### Reporting summary

Further information on research design is available in the Nature Portfolio Reporting Summary linked to this article.

## Supplementary information

Supplementary Information

Description of Additional Supplementary Information

Supplementary Data 1

Supplementary Data 2

Supplementary Data 3

Reporting Summary

Transparent Peer Review file

## Source data

Source Data

## Data Availability

Source data are provided as a Source Data file with this paper. TEMPO-based detection and Sanger sequence result for human samples are provided in Supplementary Data 1. All nucleic acid sequences used in this study are provided in Supplementary Data 2. The Sanger sequencing raw data generated in this study is provided in Supplementary Data 3. Newly generated materials and assay-related information are available for academic research use, and requests should be directed to the corresponding author. Source data are provided with this paper.

## Code availability

The code used in this study is available in a public GitHub repository (https://github.com/zibin-zhao/TEMPO) and archived on Zenodo^56^ with a permanent 10.5281/zenodo.20712014.
