# Supplementary material for: Thermodynamically programmed one-pot CRISPR platform for point-of-care SNP genotyping
Source: Nat Commun. 2026 Jul 7;17:8376. doi: 10.1038/s41467-026-75358-1 (PMC13473493; doi:10.1038/s41467-026-75358-1)
Supplement: Supplementary file 1 [file 41467_2026_75358_MOESM1_ESM.pdf]

Supplementary Information

Thermodynamically programmed one-pot CRISPR platform for point-of-care SNP genotyping

Xiaolong Wu[1 †], Yanan Li[1 †], Yumeng Cao[1], Zibin Zhao[1], Hongyu Lu[1], Shaochong Liang[1], Grace C.Y. Lui[2, 3], Denise P.C. Chan[3], and I-Ming Hsing[1]*

[1]Department of Chemical and Biological Engineering, The Hong Kong University of Science and Technology, Kowloon, 999077, Hong Kong, China

[2]Department of Medicine and Therapeutics, The Chinese University of Hong Kong, Shatin, 999077, Hong Kong, China

[3]S.H. Ho Research Centre for Infectious Diseases, The Chinese University of Hong Kong, Shatin, 999077, Hong Kong, China

[†] These authors contributed equally to this work.

* To whom correspondence should be addressed. Email: kehsing@ust.hk

Table of Contents

Supplementary Figure 1. Secondary structure analysis and FP/FPP ratio optimization for different targets. 4

Supplementary Figure 2. RPA validation of parallel amplification tracks using different primer combinations. 5

Supplementary Figure 3. Real-time RPA amplification kinetics under different primer configurations. 6

Supplementary Figure 4. PAM-dependent activation of Cas12a in the CRISPR assay. 7

Supplementary Figure 5. Robustness of TEMPO kinetics against magnesium ion fluctuations. 8

Supplementary Figure 6. ODEs model of TEMPO systems using different primer sets. 9

Supplementary Figure 7. Impact of primer trans-cleavage on TEMPO signal generation. 11

Supplementary Figure 8. Relocating the targeting site via primer engineering in the HIV derived DNA target. 13

Supplementary Figure 9. Analytical sensitivity of the TEMPO assay for detection of HIV derived DNA concentrations. 14

Supplementary Figure 10. Fluorescence signal distribution at the limit of detection. 15

Supplementary Figure 11. Quantitative PCR benchmarking of detection sensitivity for HIV derived DNA. 16

Supplementary Figure 12. Detection specificity of the TEMPO assay for HIV derived DNA. 17

Supplementary Figure 13. Robustness of the diagnostic system against complex matrix effects. 18

Supplementary Figure 14. Design, fabrication and validation of the microfluidic SNP chip. . 19

Supplementary Figure 15. Sensitivity and SNP discrimination for rs199476104 in tube-based assays. 20

Supplementary Figure 16. Compatibility of direct lysis buffer with the TEMPO amplification reaction. 21

Supplementary Figure 17. TEMPO CRISPR detection following direct lysis of human samples. 22

Supplementary Figure 18. Optimization of lyoprotectants for lyophilized TEMPO reaction system toward point-of-care use. 23

Supplementary Figure 19. Storage stability and shelf-life assessment of lyophilized TEMPO reagents. 24

Supplementary Figure 20. Tube-based TEMPO reactions for real-sample analysis. 25

Supplementary Figure 21. Design and validation of a dual-target microfluidic SNP chip 26

Supplementary Figure 22. Lyophilization and reconstitution in microfluidic chip. 27

Supplementary Figure 23. Microfluidic chip–based fluorescence readout for representative samples. 28

Supplementary Figure 24. ODE modelling of how nonspecific amplification influences quantitative performance. 30

Supplementary Table 1. Influenza A and SARS-CoV-2 clinical samples detection results by RT-qPCR.. 31

Supplementary Table 2. Composition of the TEMPO system. 32

Supplementary Table 3. Lyophilization formulation of the TEMPO system. 33

Supplementary Note 1: Ordinary Differential Equations (ODEs) 34

Supplementary References.. 43

Uncropped gel images... 44

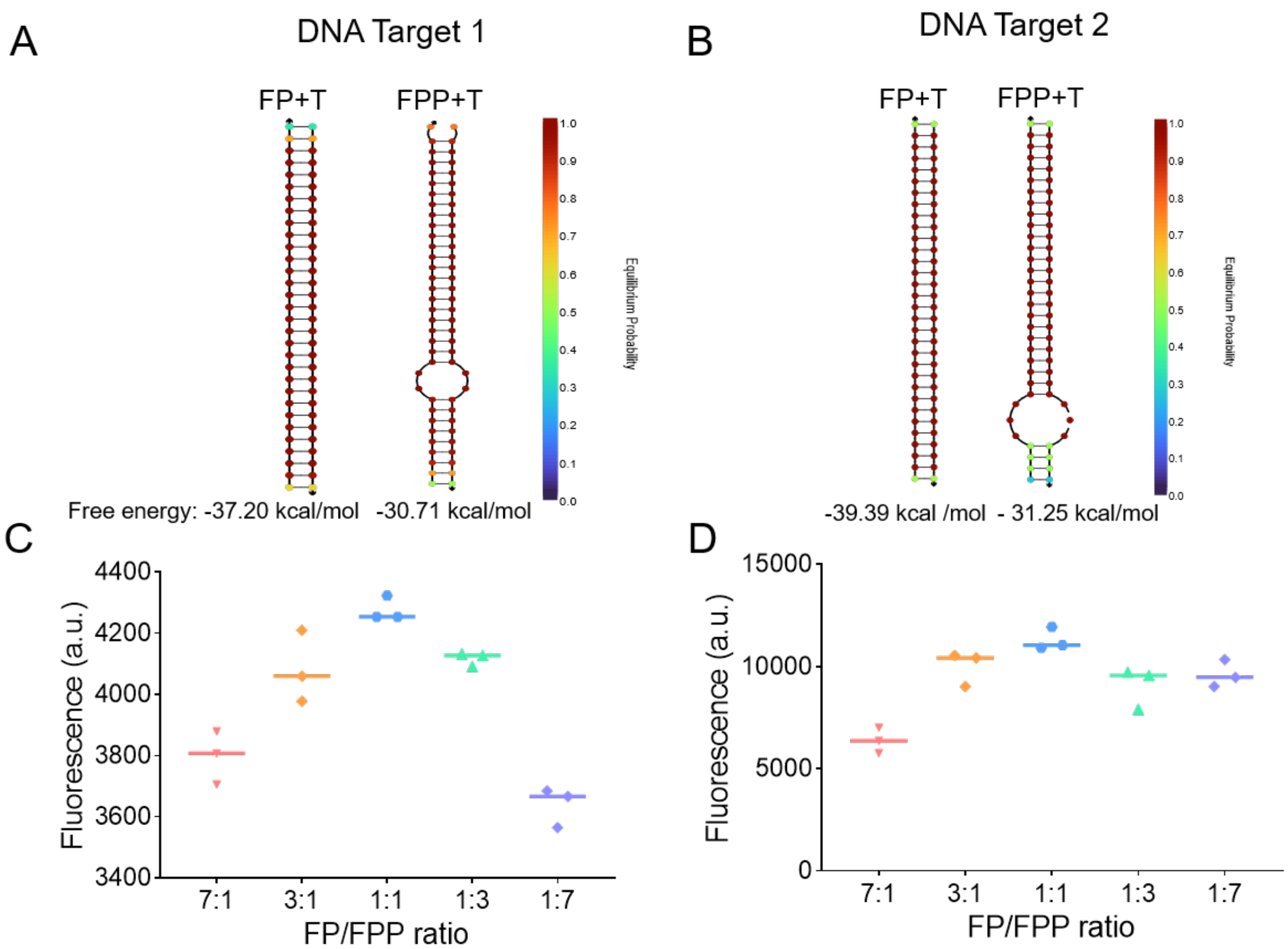

Supplementary Figure 1. Secondary structure analysis and FP/FPP ratio optimization for different targets.

(A and B) Secondary structure and hybridization-energy analysis of the forward primer (FP) and its corresponding template (T) for target 1 (HIV) (A) and target 2 (rs199476104) (B), predicted using NUPACK. Predicted base-pairing probabilities and structural features highlight differences in primer–template interactions across targets. (C and D) Optimization of the FP/FPP primer ratio for target 1 (HIV) (C) and target 2 (rs199476104) (D). Reactions were conducted with an input template concentration of 1 fM. Fluorescence responses across different FP/FPP ratios identify target-dependent optimal primer compositions. NTC denotes the non-template control. n = 3 technical replicates. All fluorescence intensity is reported in arbitrary units (a.u.). Source data are provided as a Source Data file.

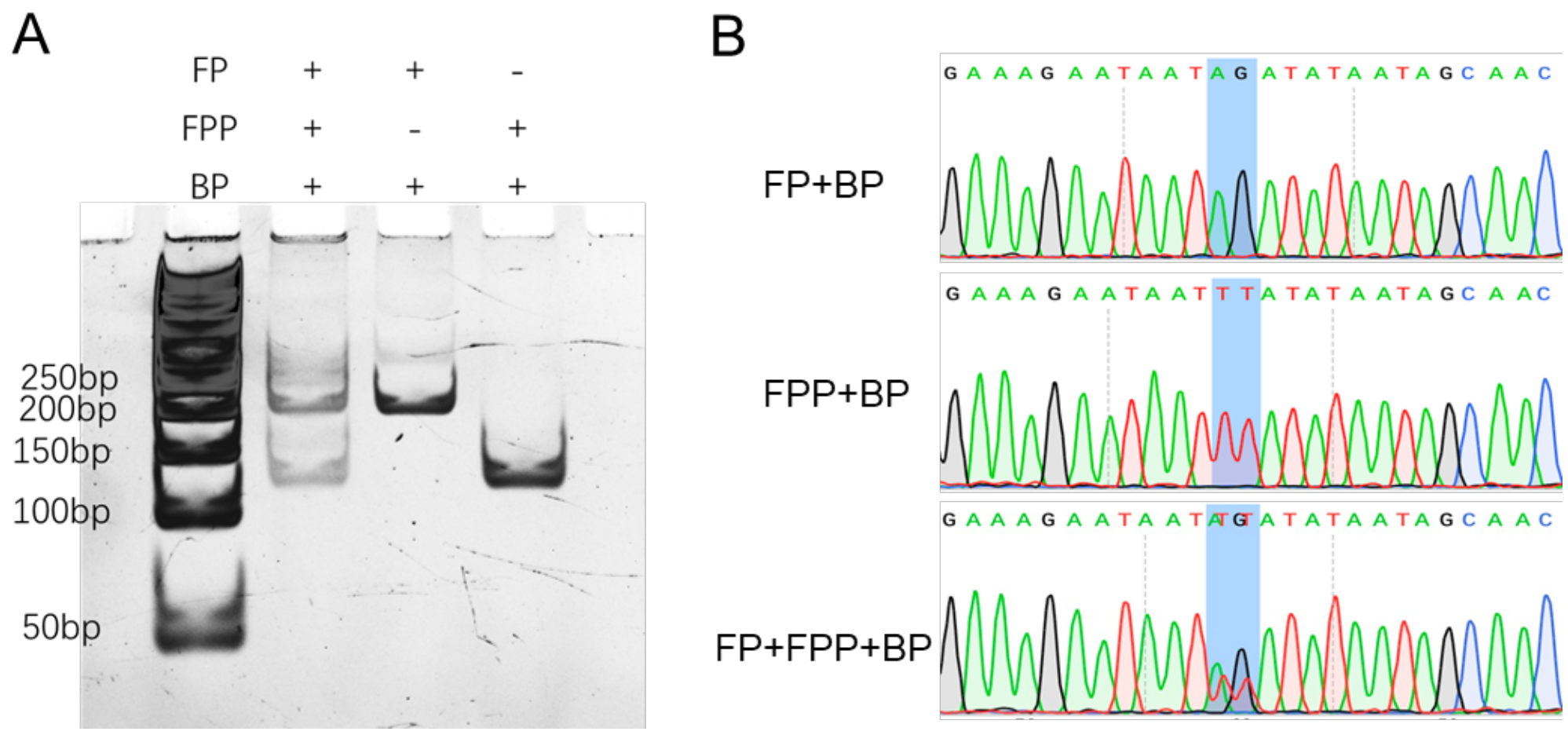

Supplementary Figure 2. RPA validation of parallel amplification tracks using different primer combinations.

Polyacrylamide gel electrophoresis (PAGE) analysis (A) and Sanger sequencing results (B) of RPA amplification products generated using different primer combinations (FP + BP, FPP + BP, and FP + FPP + BP) with HIV derived DNA as the target sequence. Distinct band patterns observed by PAGE, together with sequence verification by Sanger sequencing, confirm the formation of specific amplification products corresponding to different primer sets. These RPA assays support the presence of two parallel amplification tracks enabled by primer design. Representative gel image from 2 independent experiments with similar results.

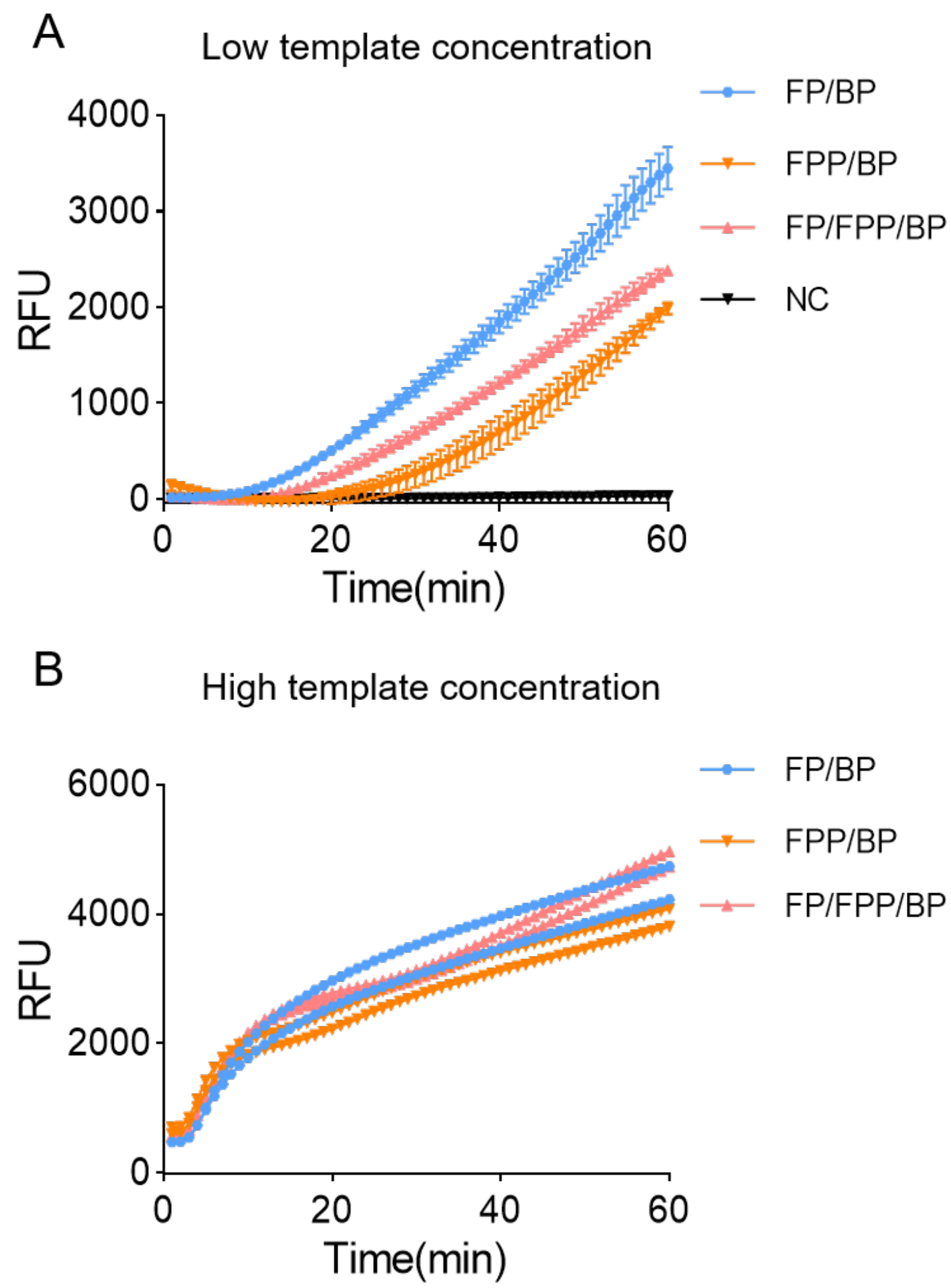

Supplementary Figure 3. Real-time RPA amplification kinetics under different primer configurations.

Real-time fluorescence kinetics of recombinase polymerase amplification (RPA) reactions were monitored using SYTO 82 (1 µM) as a fluorescent DNA-binding dye, with signals recorded in the HEX channel. (A) Low-template condition with an input template concentration of 50 aM. NC (negative control); n = 3 technical replicates; error bars indicate mean ± SD. (B) High-template condition with an input template concentration of 50 fM. Each curve represents an individual replicate (n = 2). All fluorescence intensity is reported in relative fluorescence units (RFU). Source data are provided as a Source Data file.

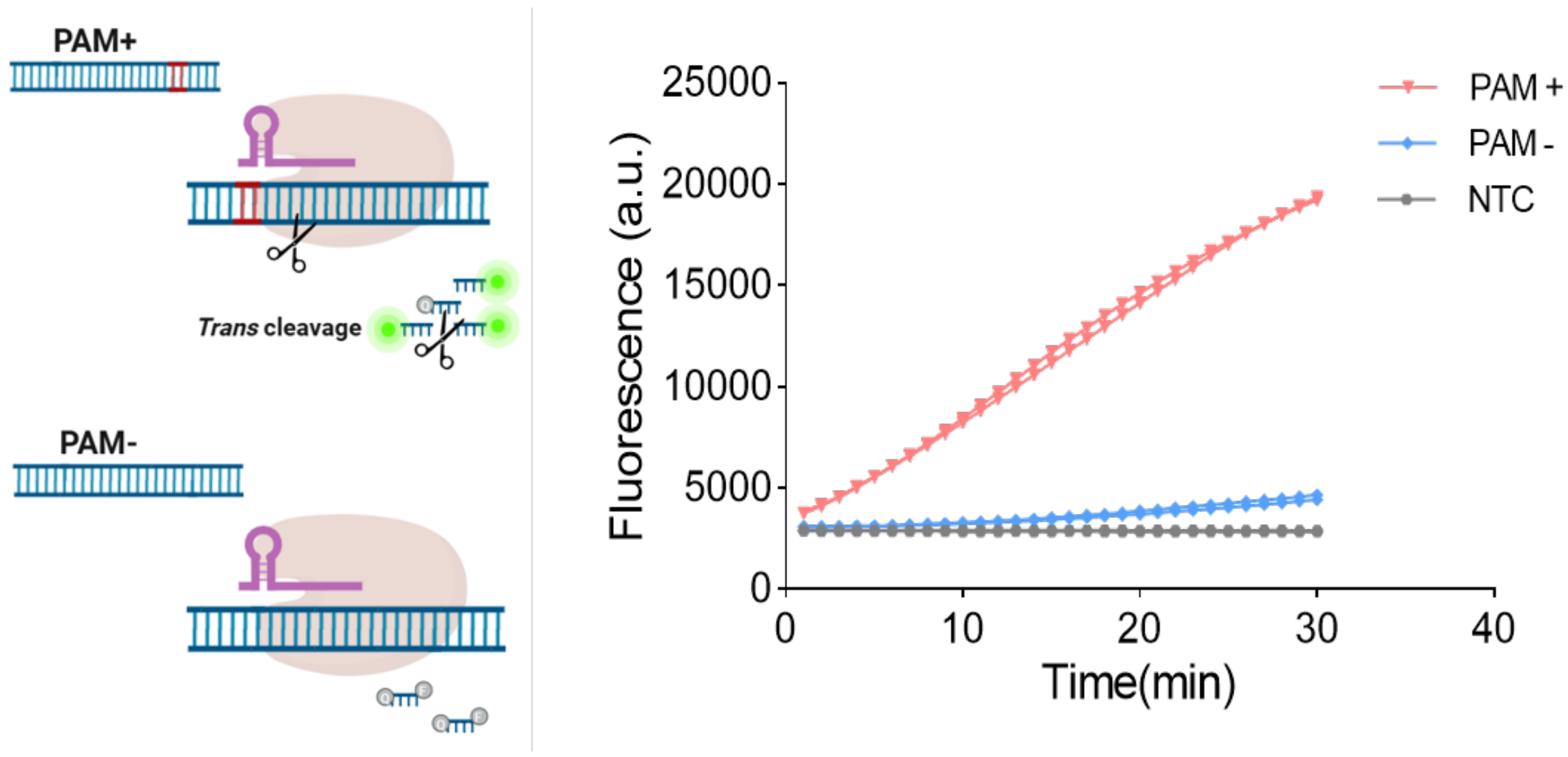

Supplementary Figure 4. PAM-dependent activation of Cas12a in the CRISPR assay.

CRISPR assays were performed using 1 nM of template sequences either containing a protospacer adjacent motif (PAM $^{+}$) or lacking a PAM site (PAM $^{-}$). In the PAM $^{+}$ condition, Cas12a–crRNA complexes efficiently recognized the target and became activated, resulting in robust trans-cleavage of the ssDNA reporter and strong fluorescence signals after incubation at 37 °C. In contrast, templates lacking a PAM sequence (PAM $^{-}$) induced minimal Cas12a activation and produced only weak fluorescence output. These results demonstrate that the presence of a PAM site in the template is essential for efficient CRISPR activation and reporter signal generation. NTC denotes the non-template control. Each curve represents an individual replicate (n = 2). All fluorescence intensity is reported in arbitrary units (a.u.). (Created in BioRender. Hsing, I. (2026) https://BioRender.com/97501wq). Source data are provided as a Source Data file.

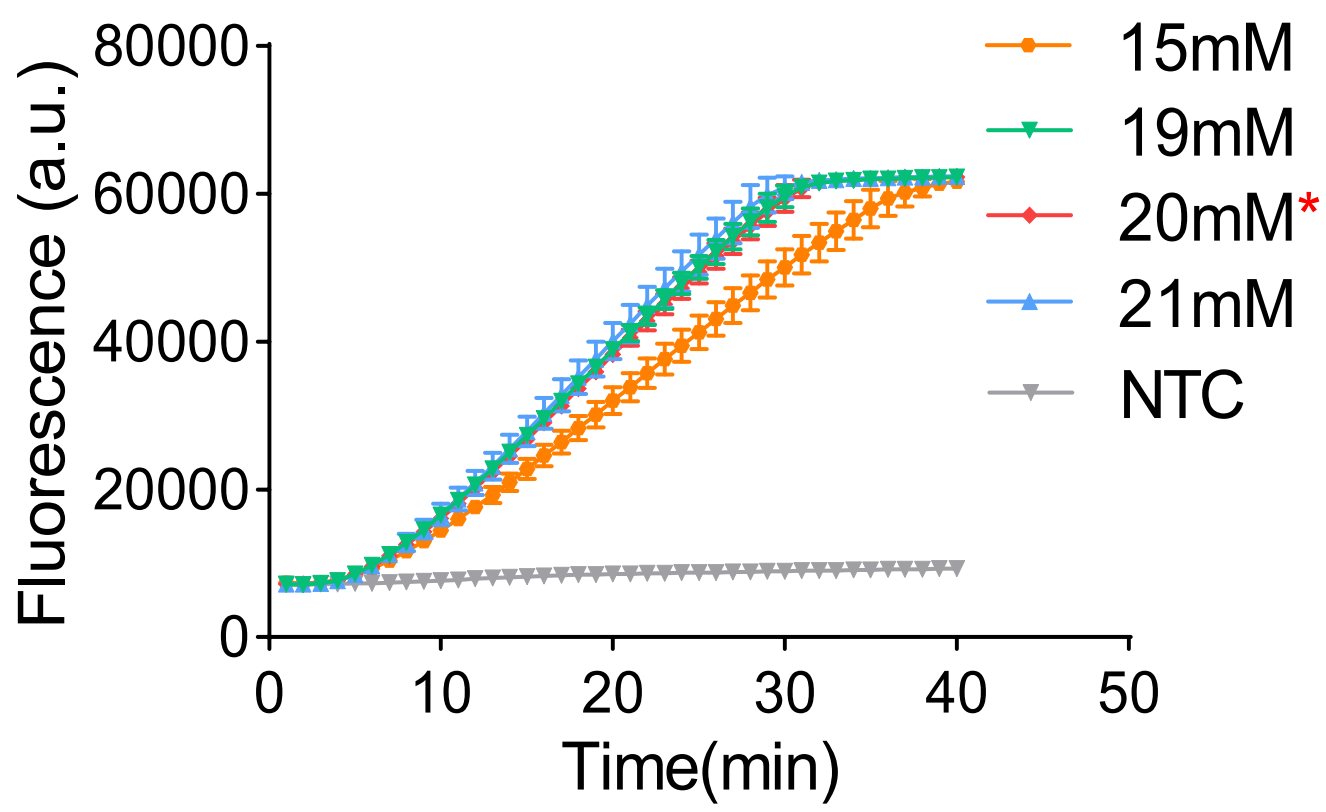

Supplementary Figure 5. Robustness of TEMPO kinetics against magnesium ion fluctuations.

To evaluate the impact of "thermodynamic drift" caused by potential Mg^{2+} depletion during RPA amplification, we monitored the fluorescence trajectories at varying initial magnesium acetate concentrations (15, 19, 20, and 21 mM). The overlapping kinetic curves across this concentration gradient demonstrate that the preset thermodynamic equilibrium is highly resistant to fluctuations in the ionic environment. 20 mM* denotes the standard concentration used in this study. NTC, non-template control. n = 3 technical replicates; error bars represent the mean ± SD. In RPA, the main sink of free Mg^{2+} is expected to be pyrophosphate (PPi) released during nucleotide incorporation. Since each incorporated nucleotide releases 1 mol of PPi, and each mol of PPi can sequester up to 2 mol of Mg^{2+}, the maximal Mg^{2+} decrease in our system can be estimated stoichiometrically. Assuming 400 nM primer consumption and generation of a 200 bp amplicon, the maximal product concentration would be ~400 nM, corresponding to ~0.16 mM incorporated nucleotides and a theoretical Mg^{2+} decrease of ~0.32 mM, which is small relative to the initial 20 mM Mg^{2+} concentration[1]. All fluorescence intensity is reported in arbitrary units (a.u.). Source data are provided as a Source Data file.

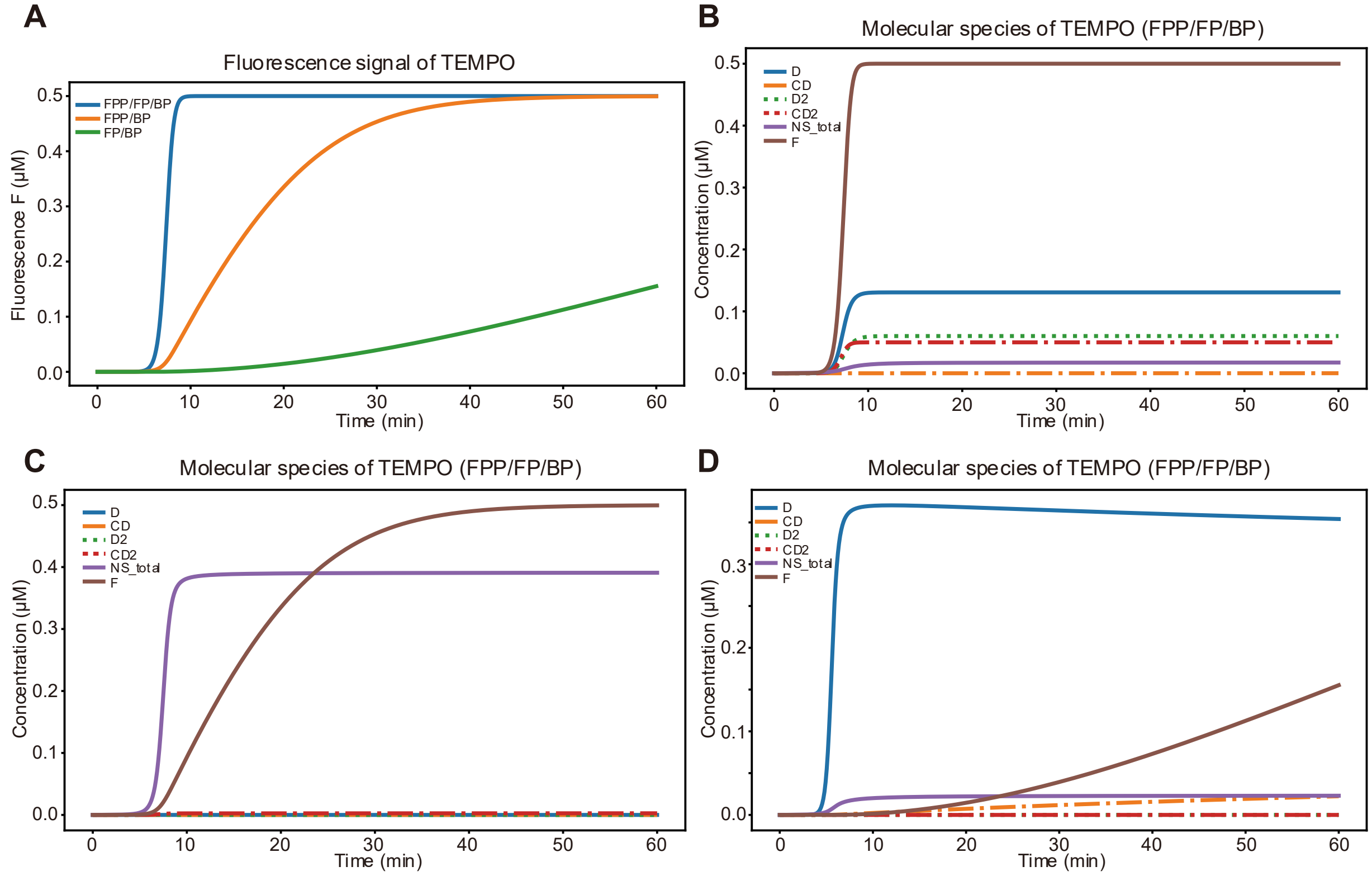

Supplementary Figure 6. ODEs model of TEMPO systems using different primer sets.

A system of ordinary differential equations (ODEs) was implemented in Python to simulate the reaction kinetics of the TEMPO assay under experimentally matched conditions. Primer and template concentrations were set to FP = 200 nM, FPP = 200 nM, BP = 400 nM, and target DNA (D) = 1 fM. Kinetic parameters (*k*cat and Km) for LbCas12a were adopted from previously reported literature[2]. Primer–template binding and extension were modeled using a uniform rate constant for fully complementary interactions (0.1 $\mu M^{-1} s^{-1}$), while mismatched primer binding (e.g., FPP binding to ST templates) was assigned a reduced rate constant (0.08 $\mu M^{-1} s^{-1}$). Cas12a–crRNA activation and trans-cleavage efficiencies were modeled as strongly PAM-dependent, with substantially higher activity in the presence of a PAM sequence. FP denotes the upstream forward primer lacking a PAM, FPP denotes the PAM-introducing forward primer, BP denotes the backward primer, D denotes the original PAM-free template, and D2 denotes PAM-containing amplicons generated via FPP-mediated amplification. CD and CD2 represent CRISPR complexes bound to D and D2 amplicons, respectively, and F denotes cleaved fluorescent reporters. (A) Simulated fluorescence output (F) of the TEMPO system using different primer combinations.
(B) Time-resolved concentration profiles of key molecular species in reactions containing FP, FPP, and BP, where both PAM-free (D) and PAM-containing (D2) amplicons are generated, enabling concurrent amplification and CRISPR signal transduction. (C) Time-resolved concentration profiles of key molecular species in reactions containing FPP and BP only, in which PAM-containing amplicons (D2) are produced and concurrently consumed by Cas12a, resulting in

attenuated amplification dynamics. (D) Time-resolved concentration profiles of key molecular species in reactions containing FP and BP only, where rapid accumulation of PAM-free amplicons (D) fails to efficiently activate Cas12a, yielding minimal fluorescence output.

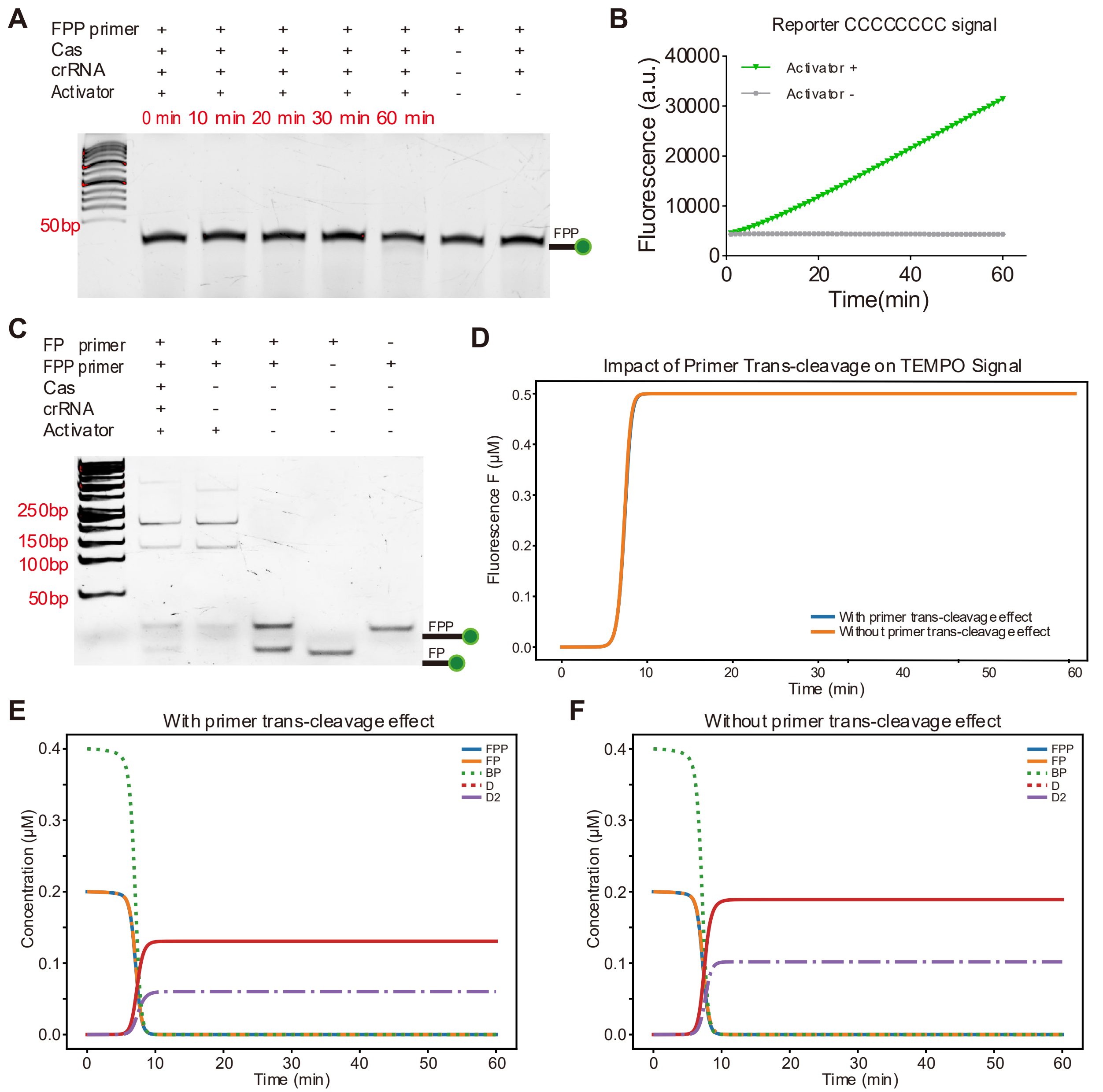

Supplementary Figure 7. Impact of primer trans-cleavage on TEMPO signal generation.

(A) Time-course analysis of primer stability under Cas12a trans-cleavage activity. Gel electrophoresis illustrates the impact of activated Cas12a on primer integrity across a series of incubation time points (0, 10, 20, 30, and 60 min). Representative gel image from 2 independent experiments with similar results. (B) Positive control confirming that, under the same experimental conditions as in A, activated Cas12a efficiently cleaved the fluorescent reporter. n = 3 technical replicates; error bars indicate mean ± SD. Fluorescence intensity is reported in arbitrary units (a.u.). (C) Gel electrophoresis of RPA products subjected to Cas12a trans-cleavage. Results indicate limited degradation of the target amplicons within a 30-min timeframe post-activation of the Cas12a-crRNA complex. Representative gel image from 2 independent experiments with similar

results. (D) Simulated signal trajectories in the ODE model with primer trans-cleavage. Experimental results showed that activated CRISPR–Cas12a exhibited only weak trans-cleavage activity toward primers. Consistent with these observations, the ODE model indicated that primer trans-cleavage slightly reduced amplicon yield but produced no observable effect on fluorescence signal generation in the one-pot reaction. (E and F) Simulated variation of molecular species in the ODE model with (E) or without (F) primer trans-cleavage. Source data are provided as a Source Data file.

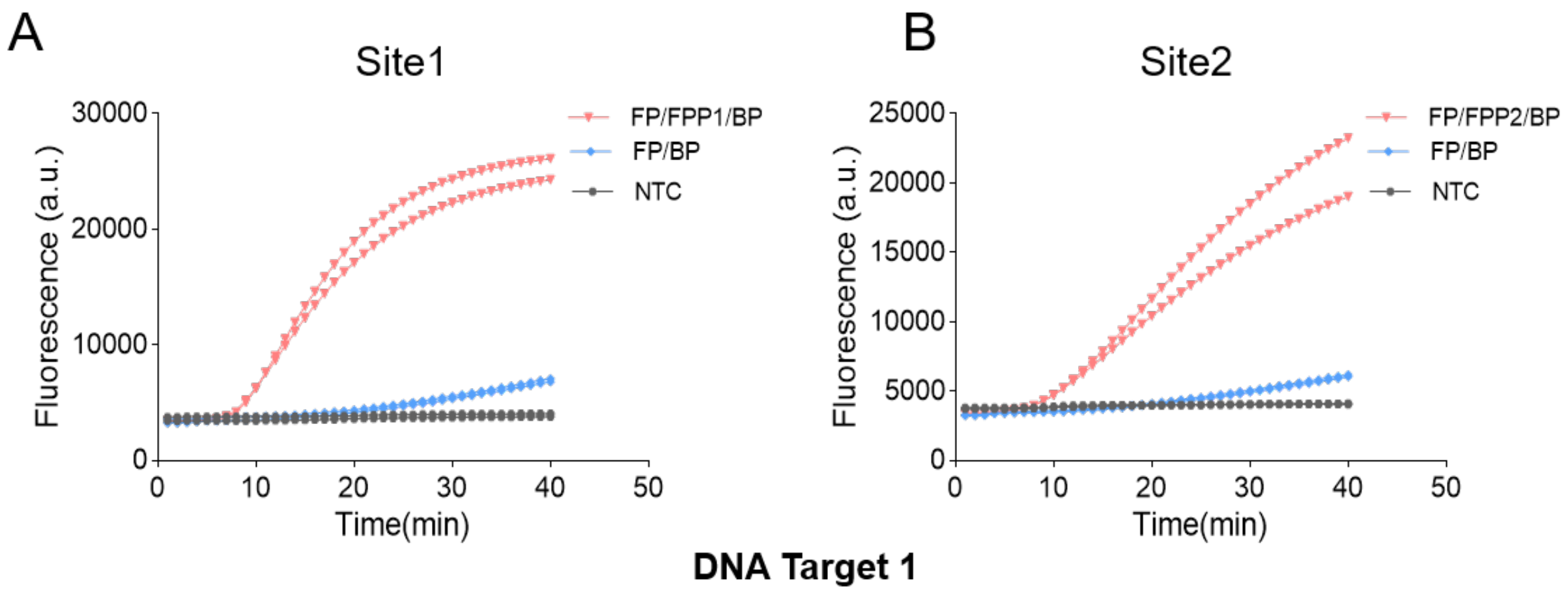

Supplementary Figure 8. Relocating the targeting site via primer engineering in the HIV derived DNA target.

Targeting site relocation was achieved by introducing forward primers encoding alternative targeting sites within the HIV derived DNA target. Real-time amplification kinetics were monitored at an input concentration of 1 fM. (A) Amplification curves obtained using the pristine forward primer or the engineered primer FPP1 targeting site 1. The pristine primer initiates amplification earlier, whereas FPP1 exhibits a delayed onset, consistent with reduced primer–template binding affinity. (B) Amplification curves obtained using the pristine forward primer or the engineered primer FPP2 targeting site 2. A similarly delayed and sequential onset of amplification is observed, demonstrating that primer engineering enables programmable relocation of the targeting site without disrupting amplification fidelity. Each curve represents an individual replicate (n = 2). All fluorescence intensity is reported in arbitrary units (a.u.). Source data are provided as a Source Data file.

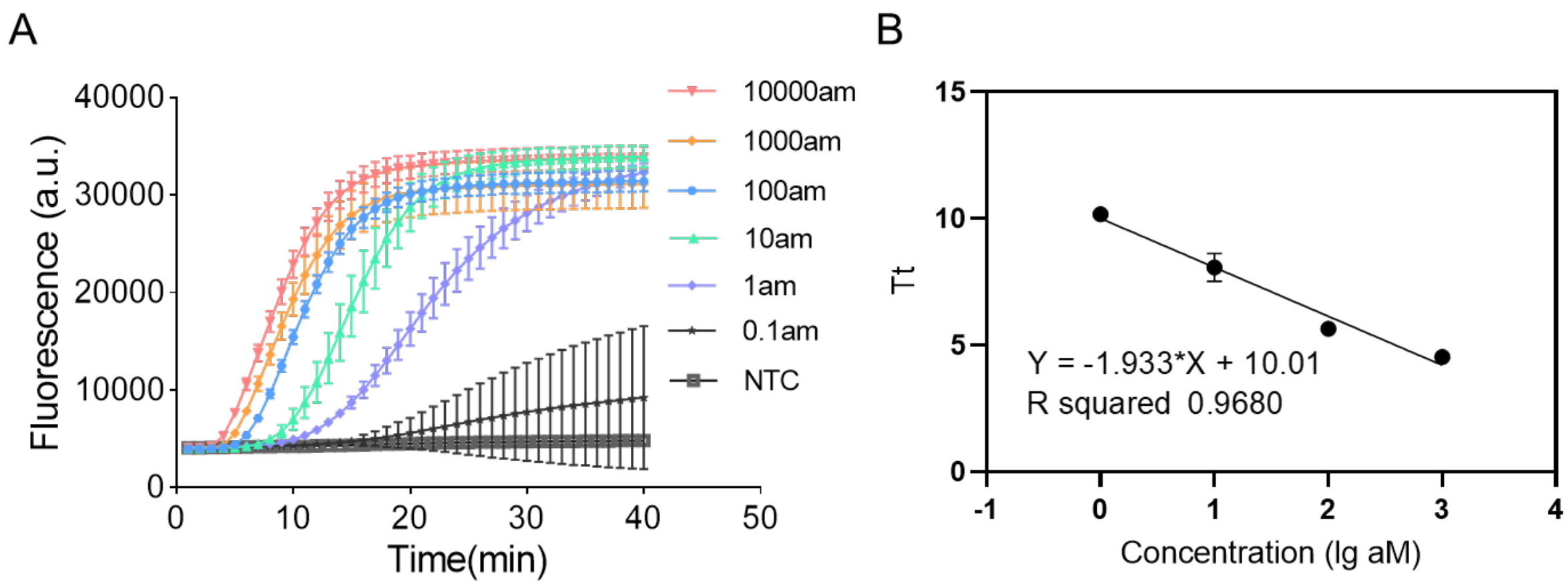

Supplementary Figure 9. Analytical sensitivity of the TEMPO assay for detection of HIV derived DNA concentrations.

(A) Real-time fluorescence amplification curves obtained using serially diluted HIV derived DNA inputs (10,000 aM, 1,000 aM, 100 aM, 10 aM, 1 aM, and 0.1 aM). Higher target concentrations produced earlier fluorescence onset and faster signal accumulation, whereas lower concentrations exhibited delayed amplification kinetics. (B) Standard curve of the detection platform. Threshold times (Tt) were determined using a fluorescence threshold of 5,000 a.u. across the tested concentration range. NTC, non-template control reaction; n = 3 technical replicates; error bars represent the means ± SD. All fluorescence intensity is reported in arbitrary units (a.u.). Source data are provided as a Source Data file.

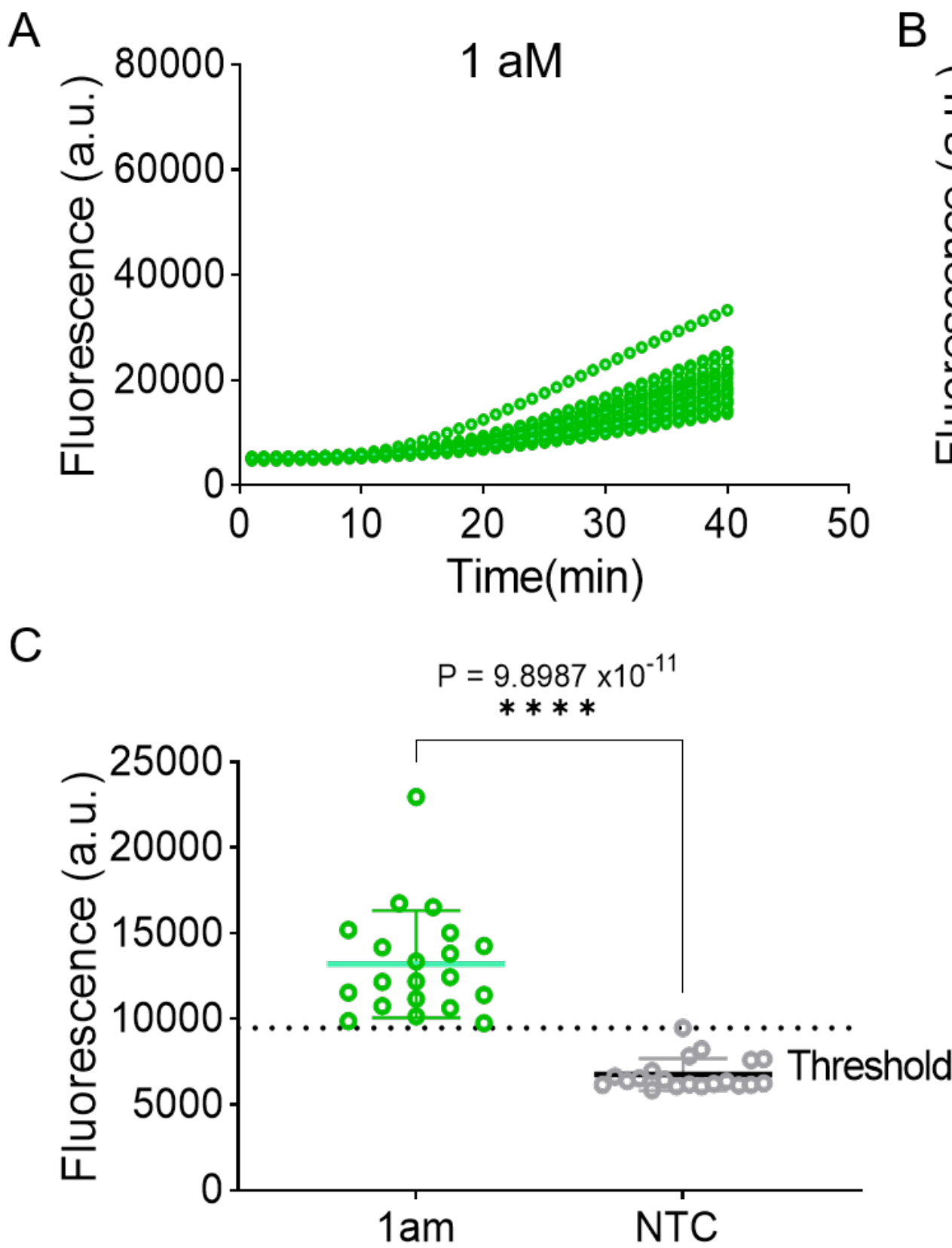

Supplementary Figure 10. Fluorescence signal distribution at the limit of detection.

(A and B) Real-time fluorescence kinetic curves of the assay in the presence of 1 aM HIV-derived DNA and non-template control (NTC). Each curve represents an individual replicate (n = 20). (C) Statistical analysis of end-point fluorescence intensity at 30 min for the 1 aM target and NTC. Each point represents an independent replicate (n = 20). The dashed line indicates the detection threshold defined as the mean fluorescence of NTC plus three standard deviations (mean + 3σ). Using this criterion, all 1 aM samples (20/20) were classified as positive, while only a small fraction of NTC replicates (1/20) exceeded the threshold, corresponding to a low false-positive rate. Statistical significance was evaluated using a two-tailed t-test (****, $P < 0.0001$). All fluorescence intensity is reported in arbitrary units (a.u.). Source data are provided as a Source Data file.

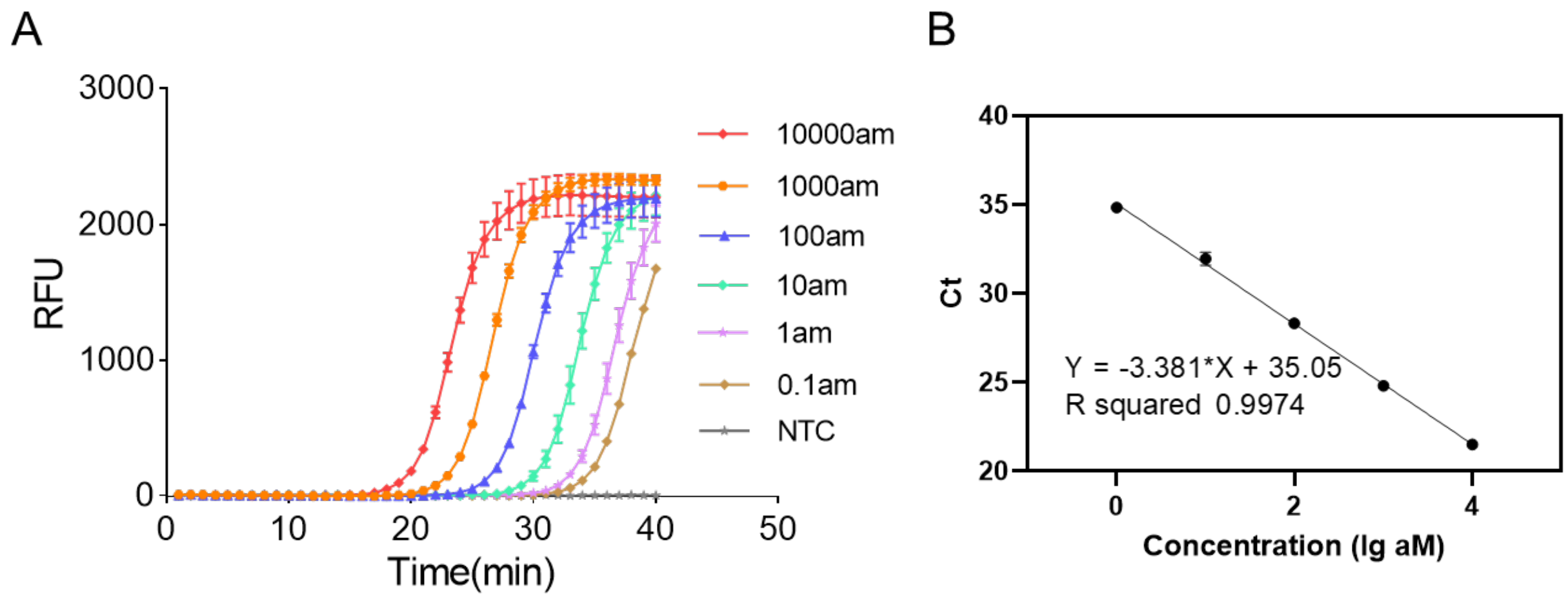

Supplementary Figure 11. Quantitative PCR benchmarking of detection sensitivity for HIV derived DNA.

Quantitative PCR (qPCR) was performed as a reference method to benchmark the analytical sensitivity of HIV derived DNA detection. (A) Representative real-time fluorescence amplification curves obtained by qPCR across a series of HIV derived DNA target concentrations (10000am, 1000am, 100am, 10am, 1am, 0.1am). NTC: non-template control. n = 3 technical replicates; error bars represent the mean ± SD. Fluorescence intensity is reported in relative fluorescence units (RFU). (B) Corresponding cycle threshold (Ct) values derived from the qPCR amplification curves, enabling quantitative comparison across input concentrations. Source data are provided as a Source Data file.

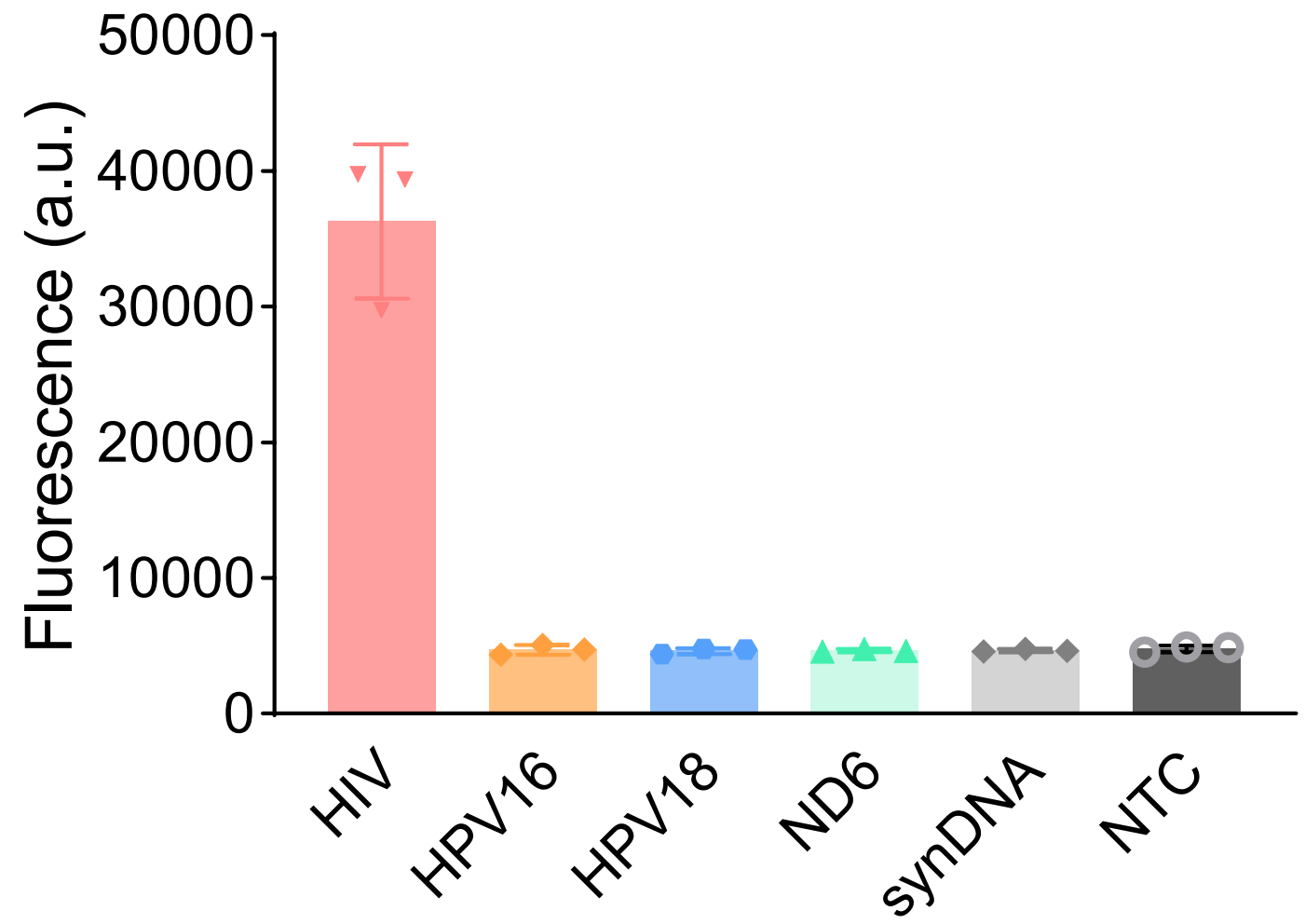

Supplementary Figure 12. Detection specificity of the TEMPO assay for HIV derived DNA.

The specificity of the HIV TEMPO assay was evaluated using non-target DNA templates, including ND6, HPV16, HPV18 plasmids, and a synthetic DNA sequence absent from natural genomes, with a non-template control (NTC) included as a negative control. All templates were tested at an input concentration of 1 fM. The assay generated a strong fluorescence signal exclusively in the presence of the target HIV derived DNA template, whereas non-target natural and synthetic DNAs produced only background-level signals comparable to the NTC. n = 3 technical replicates; error bars indicate mean ± SD. These results demonstrate the high specificity of the TEMPO system, with selective activation occurring only for the intended target sequence. All fluorescence intensity is reported in arbitrary units (a.u.). Source data are provided as a Source Data file.

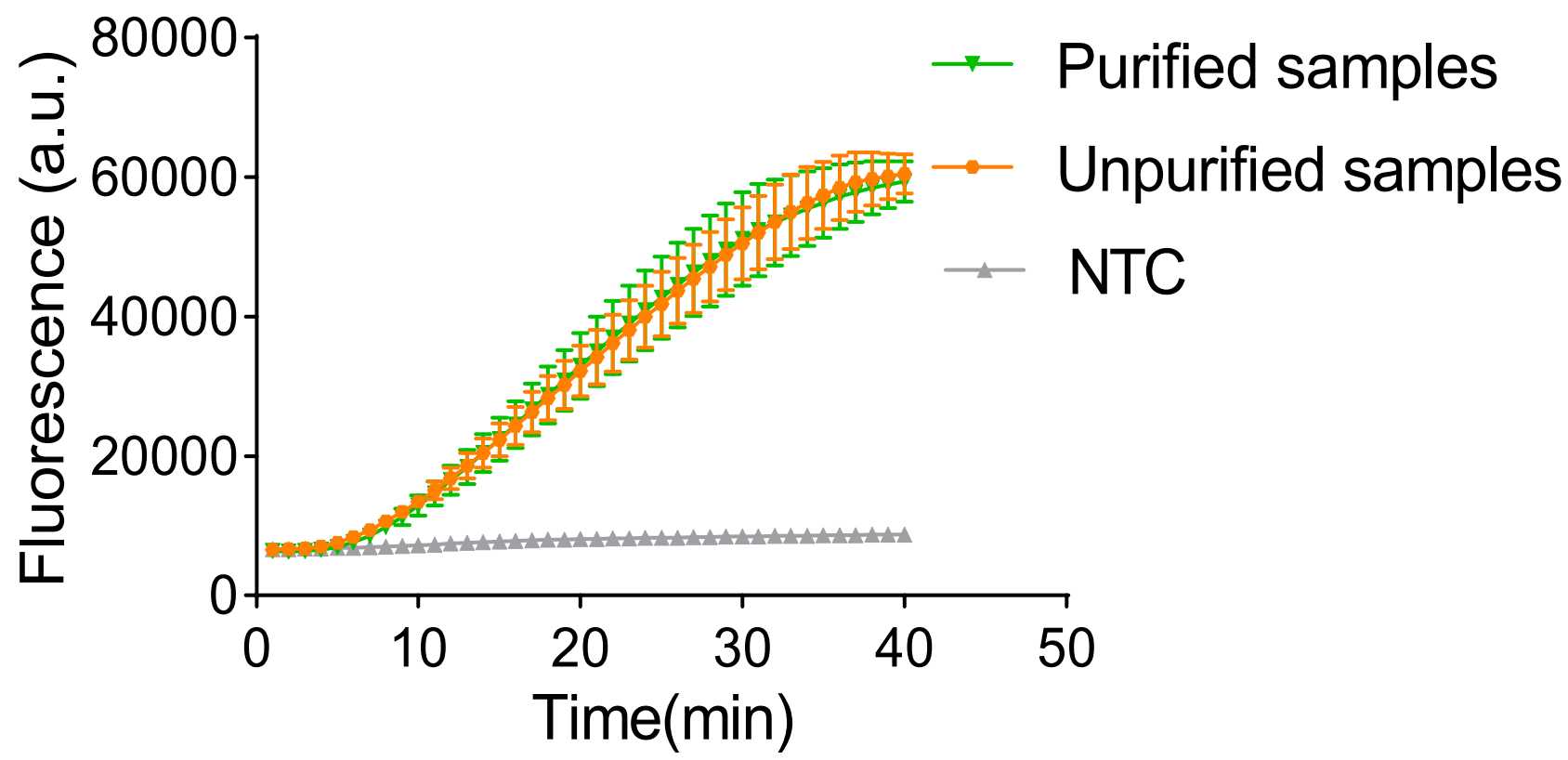

Supplementary Figure 13. Robustness of the diagnostic system against complex matrix effects.

Real-time fluorescence kinetic curves were compared between the positive control (PC, with purified templates) and the unpurified samples (crude human clinical extracts) to evaluate the impact of matrix interference. NTC, non-template control reaction; n = 3 technical replicates; error bars represent the means ± SD. All fluorescence intensity is reported in arbitrary units (a.u.). Source data are provided as a Source Data file.

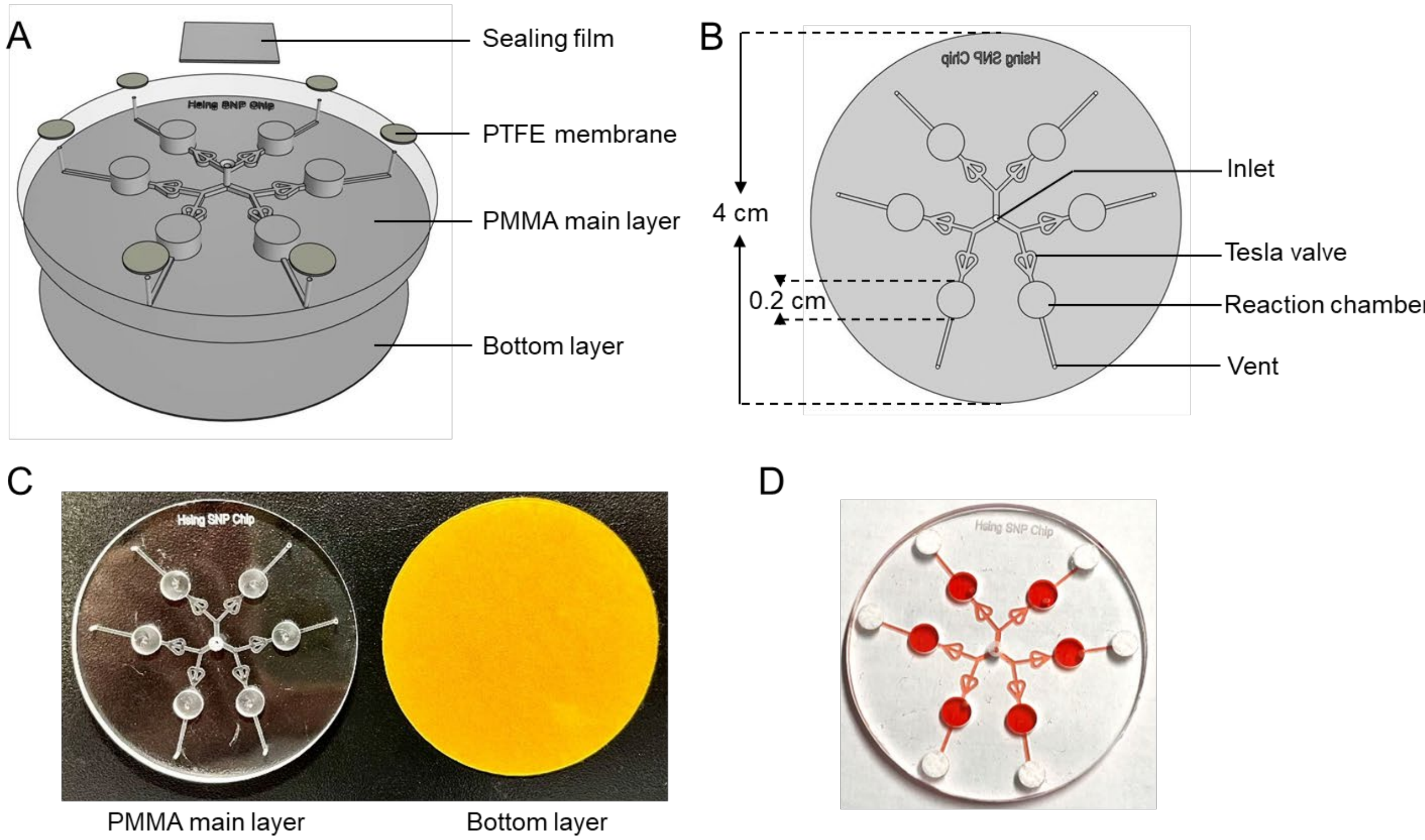

Supplementary Figure 14. Design, fabrication and validation of the microfluidic SNP chip.

(A) Schematic illustration of the overall microfluidic chip architecture. The chip comprises four vertically stacked layers from bottom to top: a single-sided adhesive film for channel sealing, a PMMA microfluidic channel layer, a hydrophobic PTFE vent membrane that permits gas exchange while preventing liquid leakage, and a top single-sided adhesive film used to seal the sample inlet. (B) Bottom-view design of the PMMA microfluidic channel layer integrating three independent SNP targets in a six-channel architecture. The circular chip (4 cm in diameter) incorporates a central sample inlet, Tesla-type valve microstructures for passive flow regulation, radially distributed reaction chambers, and air vents, enabling controlled fluid handling and reaction compartmentalization. (C) Photograph of the PMMA channel layer and the bottom single-sided adhesive film prior to assembly. (D) Photograph of the fully assembled microfluidic chip filled with red dye to validate channel integrity and connectivity.

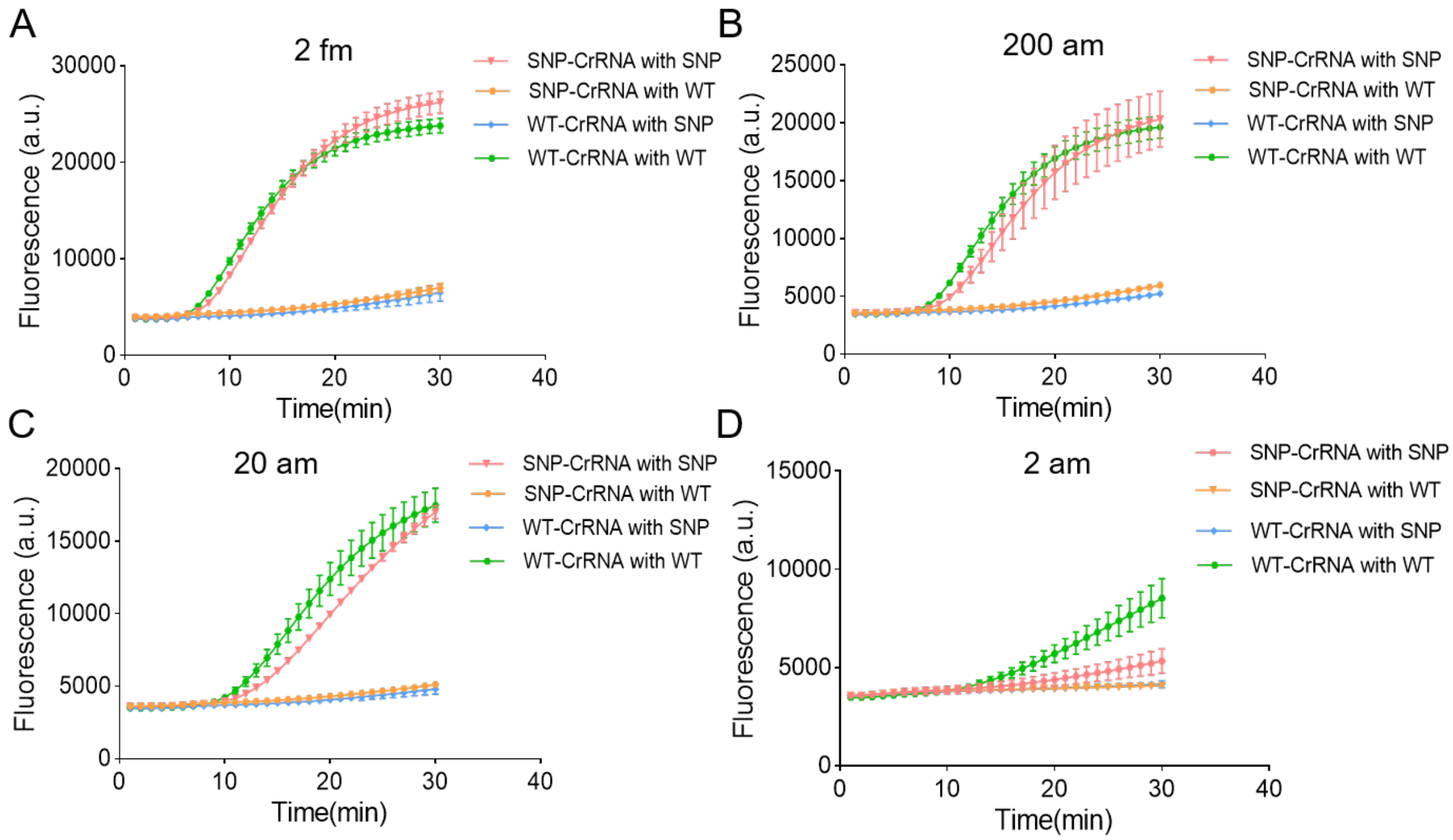

Supplementary Figure 15. Sensitivity and SNP discrimination for rs199476104 in tube-based assays.

Real-time fluorescence kinetics of WT and SNP targets at the rs199476104 locus. Reactions were carried out in test tubes using serially diluted target DNA inputs of 2 fM (A), 200 aM (B), 20 aM (C), and 2 aM (D). WT and SNP alleles exhibited distinct amplification kinetics across all concentrations, reflected by separable fluorescence trajectories over time. NTC denotes non-template controls. n = 3 technical replicates; error bars represent the mean ± SD. All fluorescence intensity is reported in arbitrary units (a.u.). Source data are provided as a Source Data file.

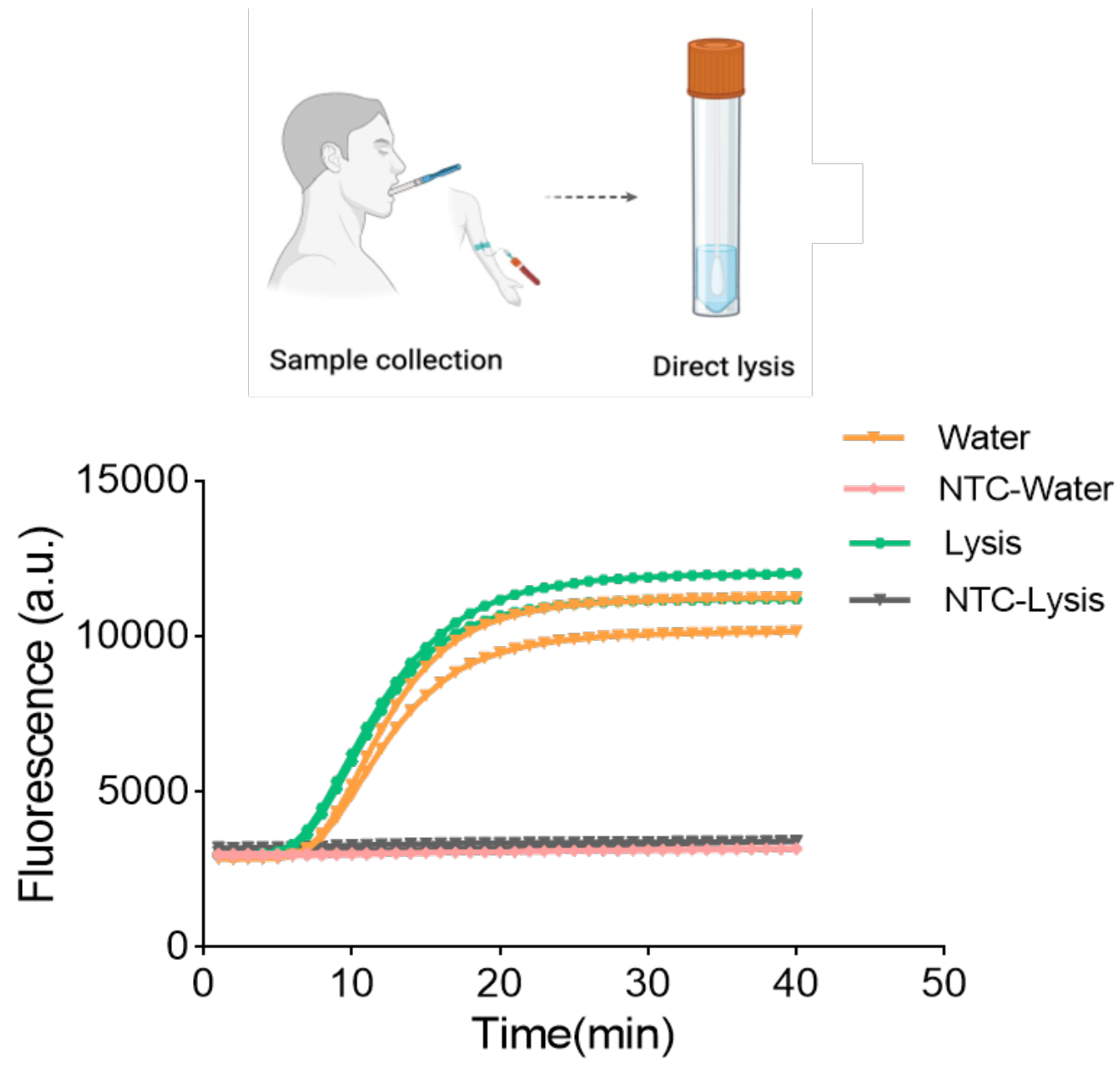

Supplementary Figure 16. Compatibility of direct lysis buffer with the TEMPO amplification reaction.

Schematic of sample collection followed by direct lysis (top) and real-time fluorescence kinetics of the TEMPO reaction in the presence of lysis buffer (bottom). Reactions were performed with a 1 fM input template. Two no-template controls (NTC–water and NTC–lysis buffer) remained at baseline throughout the assay, while comparable amplification kinetics were observed with and without lysis buffer, indicating that direct lysis does not interfere with reaction performance. Each curve represents an individual replicate (n = 2). All fluorescence intensity is reported in arbitrary units (a.u.). (Created in BioRender. Hsing, I. (2026) https://BioRender.com/1xaidy6). Source data are provided as a Source Data file.

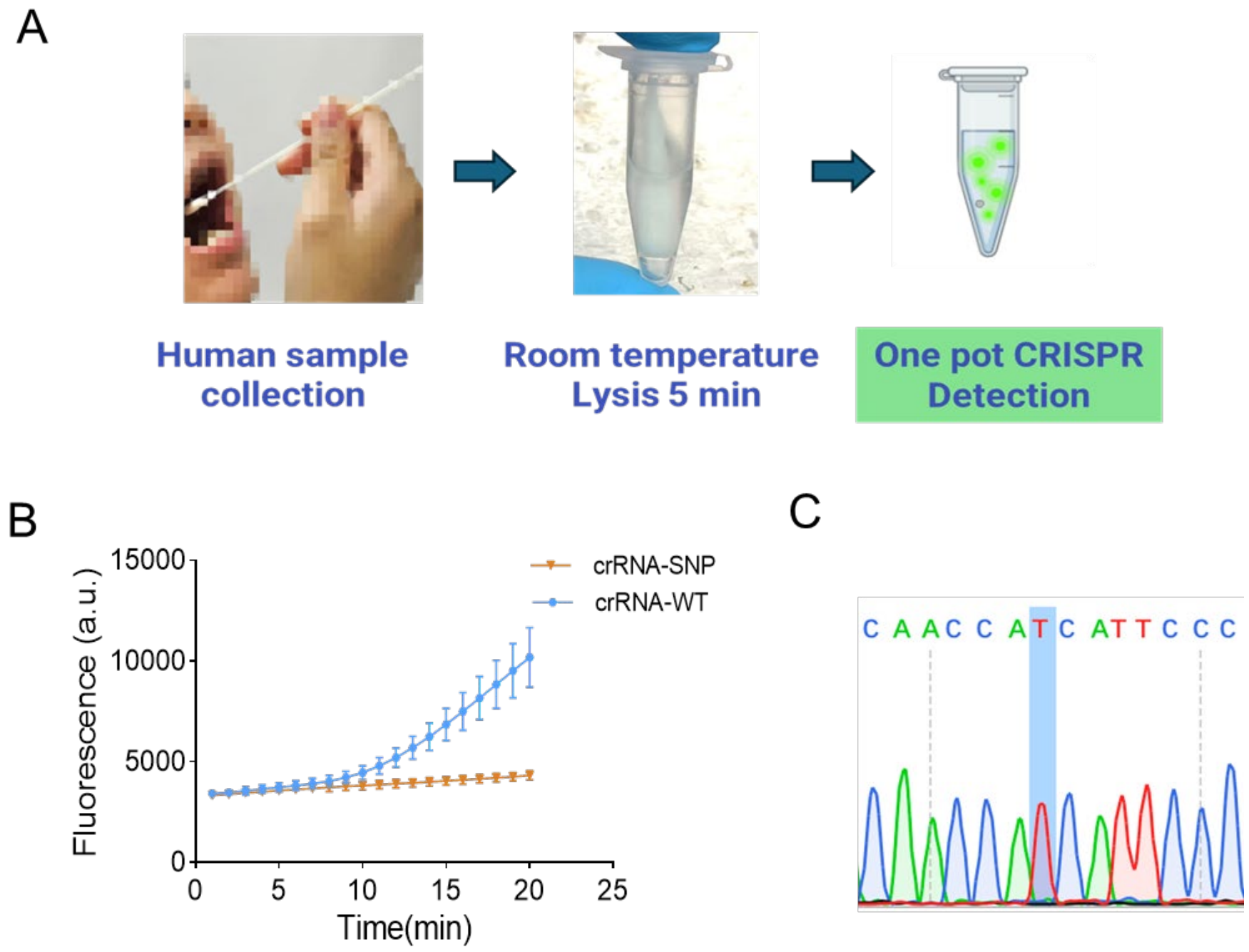

Supplementary Figure 17. TEMPO CRISPR detection following direct lysis of human samples.

(A) Schematic illustration of the workflow for TEMPO CRISPR detection following direct lysis of human samples. (B) Human samples were collected, lysed at room temperature for 5 min, and directly introduced into the one-pot CRISPR reaction without nucleic acid purification. Real-time fluorescence kinetics demonstrate successful target amplification and detection after direct lysis. n = 3 technical replicates; error bars represent the mean ± SD. (C) Sanger sequencing confirms the identity of the amplified target. All fluorescence intensity is reported in arbitrary units (a.u.). Source data are provided as a Source Data file.

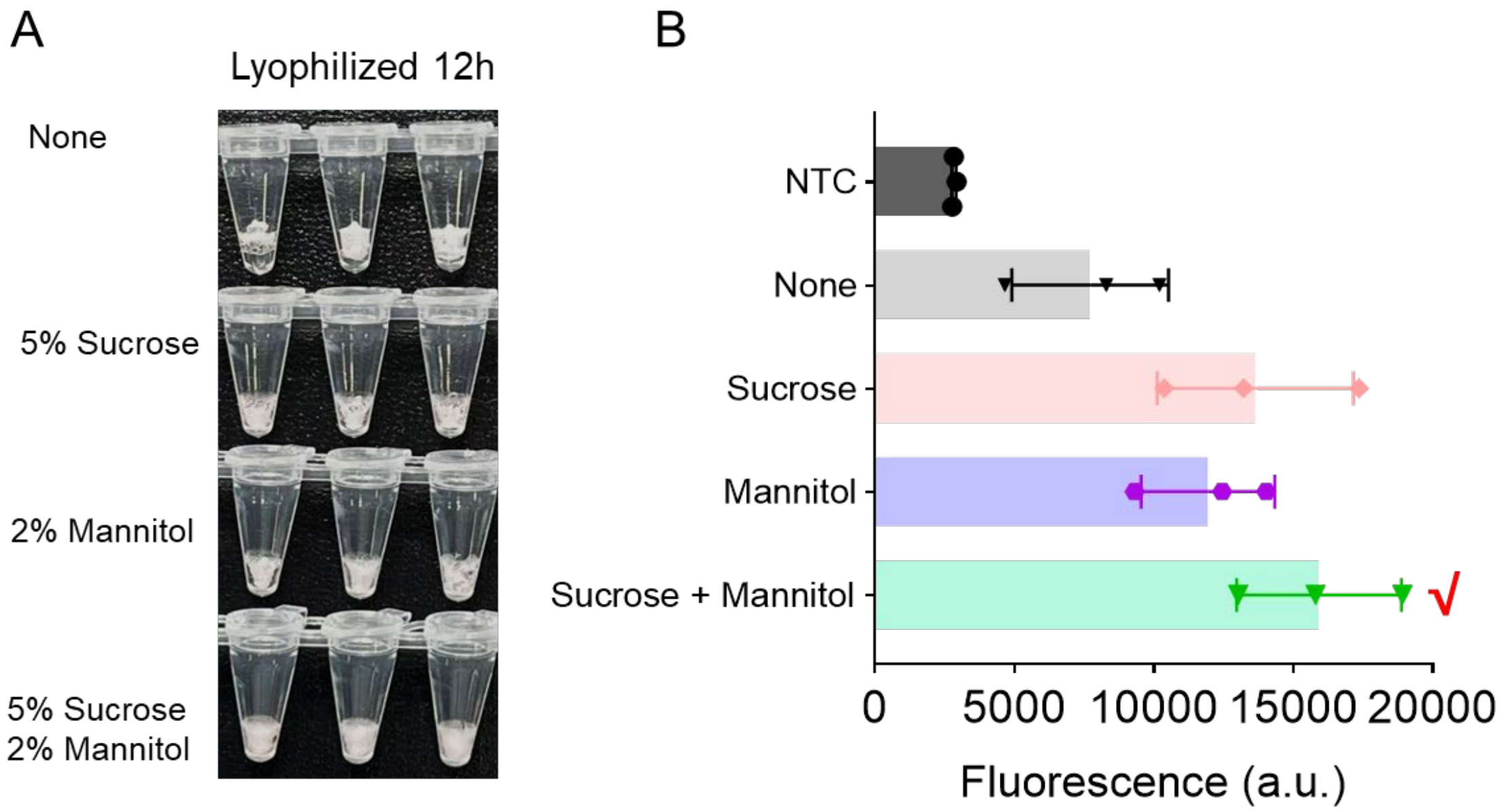

Supplementary Figure 18. Optimization of lyoprotectants for lyophilized TEMPO reaction system toward point-of-care use.

(A) Photographs of the TEMPO reaction system after 12 h lyophilization using different lyoprotectants. The reaction mixtures were lyophilized for 12 h in the presence of various lyoprotectants as indicated. None denotes the condition without the addition of any lyoprotectants. (B) Fluorescence validation of lyophilized one-pot reaction systems prepared with different lyoprotectants. Lyophilized reactions were rehydrated and challenged with 1 fM target input. Fluorescence signals were recorded after 20 min of reaction, demonstrating that 5% (w/w) sucrose combined with 2% (w/w) mannitol provided the optimal protection and preserved the highest reaction performance. n = 3 technical replicates; error bars indicate mean ± SD; NTC denotes non-template controls. All fluorescence intensity is reported in arbitrary units (a.u.). Source data are provided as a Source Data file.

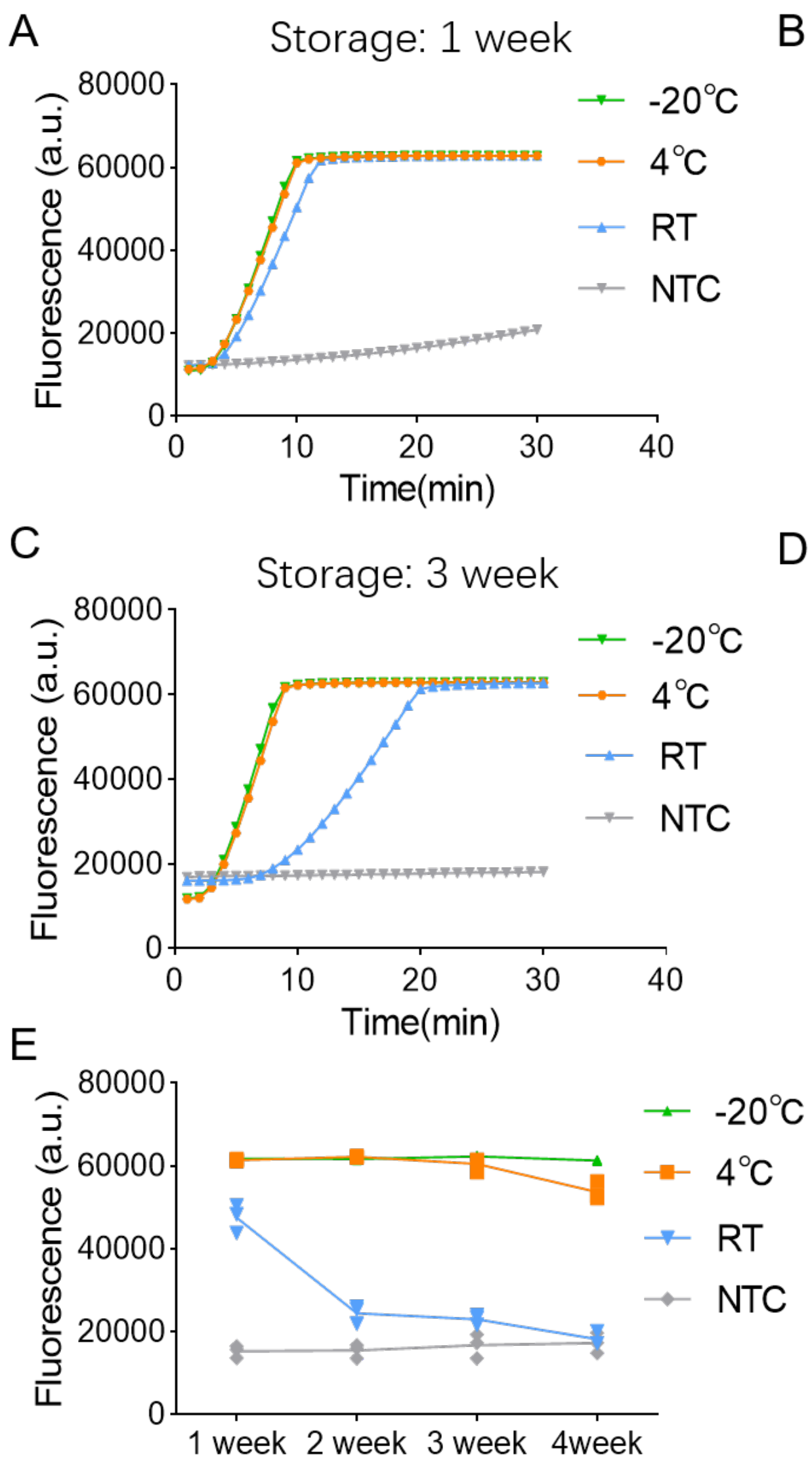

Supplementary Figure 19. Storage stability and shelf-life assessment of lyophilized TEMPO reagents.

(A-D) Real-time fluorescence kinetics of the lyophilized TEMPO assay reagents stored at various temperatures (-20°C, 4°C, and room temperature) over 4 weeks. Each panel represents a specific storage duration: 1 week, 2 weeks, 3 weeks, and 4 weeks. The performance of the reagents was evaluated by monitoring the fluorescence recovery at 30-minute intervals upon rehydration and initiation of the reaction. (E) Statistical comparison of end-point fluorescence intensities at 10 min for samples across various storage durations (1, 2, 3, and 4 weeks); n = 3 technical replicates; error bars indicate mean ± SD; NTC denotes non-template controls. All fluorescence intensity is reported in arbitrary units (a.u.). Source data are provided as a Source Data file.

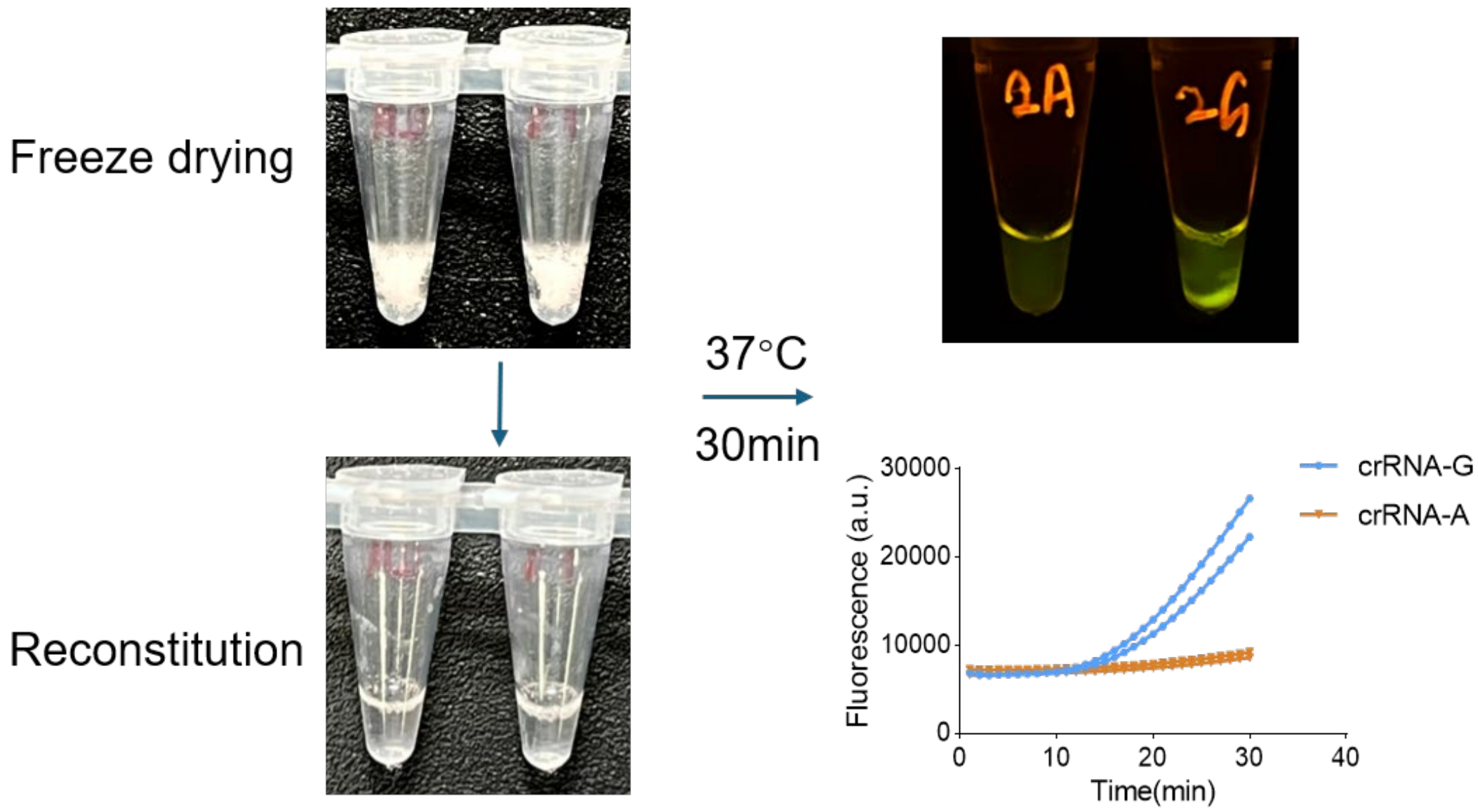

Supplementary Figure 20. Tube-based TEMPO reactions for real-sample analysis.

Photographs of the lyophilized TEMPO reaction system pre-stored in tubes before and after reconstitution with real samples (left). Following rehydration, endpoint fluorescence images illustrate distinct signal outputs between samples (top right). Real-time fluorescence kinetics obtained from a fresh, non-lyophilized reaction system are shown as a control to confirm successful amplification and detection in real samples (bottom right). Each curve represents an individual replicate (n = 2). All fluorescence intensity is reported in arbitrary units (a.u.). Source data are provided as a Source Data file.

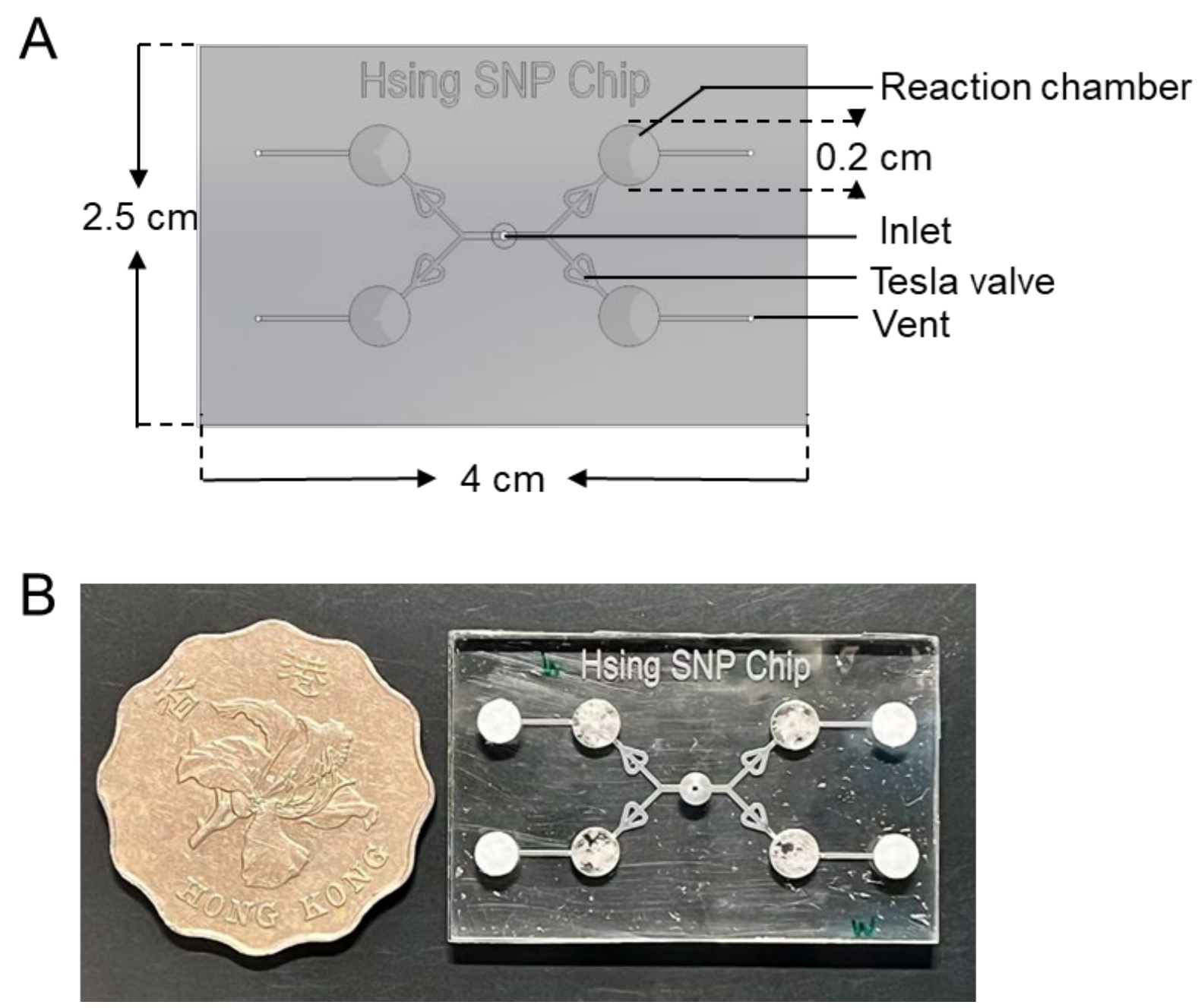

Supplementary Figure 21. Design and validation of a dual-target microfluidic SNP chip

(A) Schematic design of the microfluidic SNP chip integrating two independent SNP targets within a four-channel architecture. Each target is addressed by two parallel reaction channels converging toward a central reaction chamber, enabling multiplexed and symmetric assay operation. (B) Photograph of the fully assembled SNP chip. A HKD 2 coin is shown alongside the device for size reference.

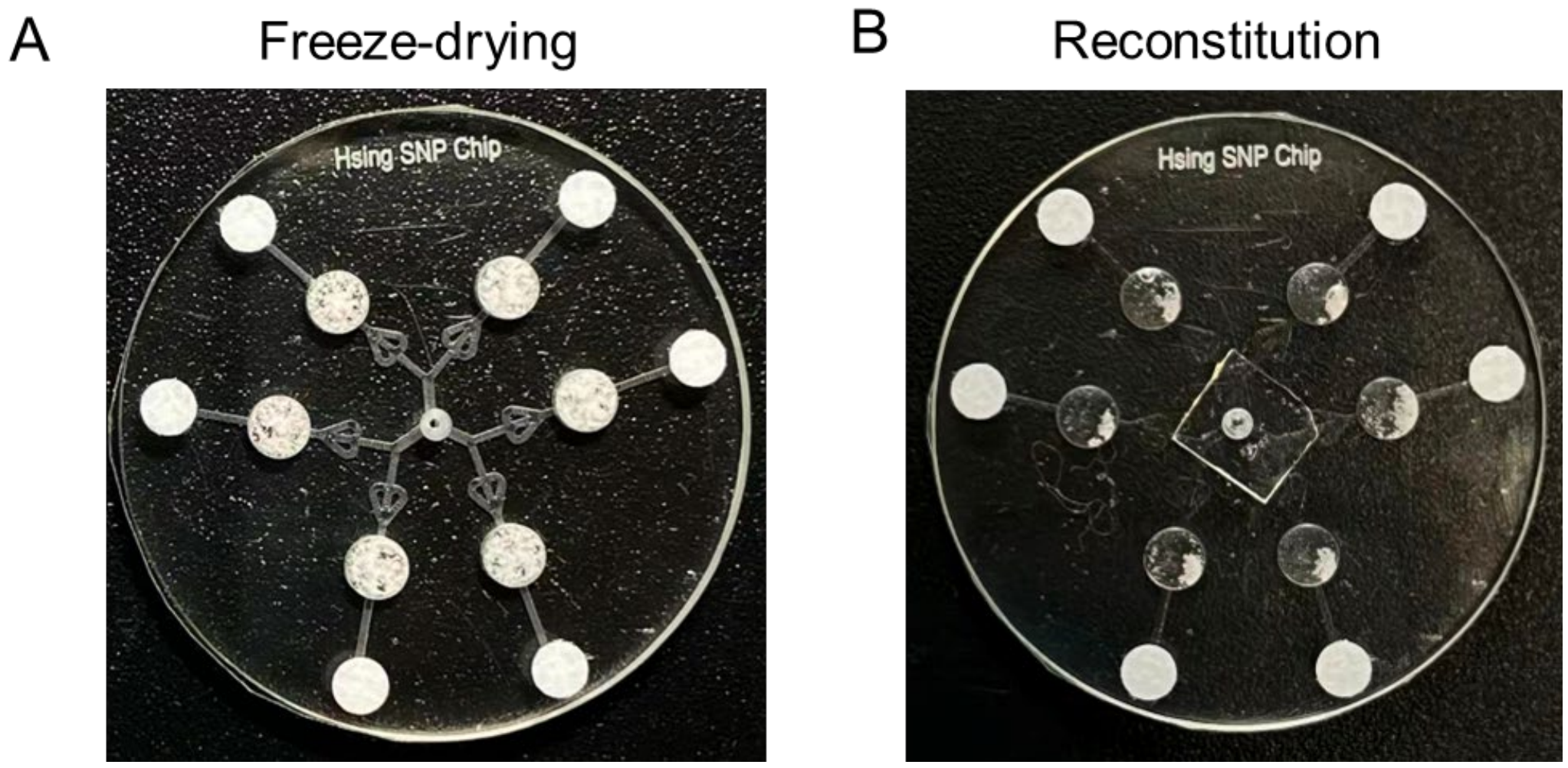

Supplementary Figure 22. Lyophilization and reconstitution in microfluidic chip.

Photographs of the microfluidic SNP chip after on-chip lyophilization (A) and following reagent reconstitution by sample loading (B). Lyophilized reaction components were preserved within the reaction chambers and rehydrated prior to analysis, demonstrating the compatibility of the chip with on-chip reagent storage.

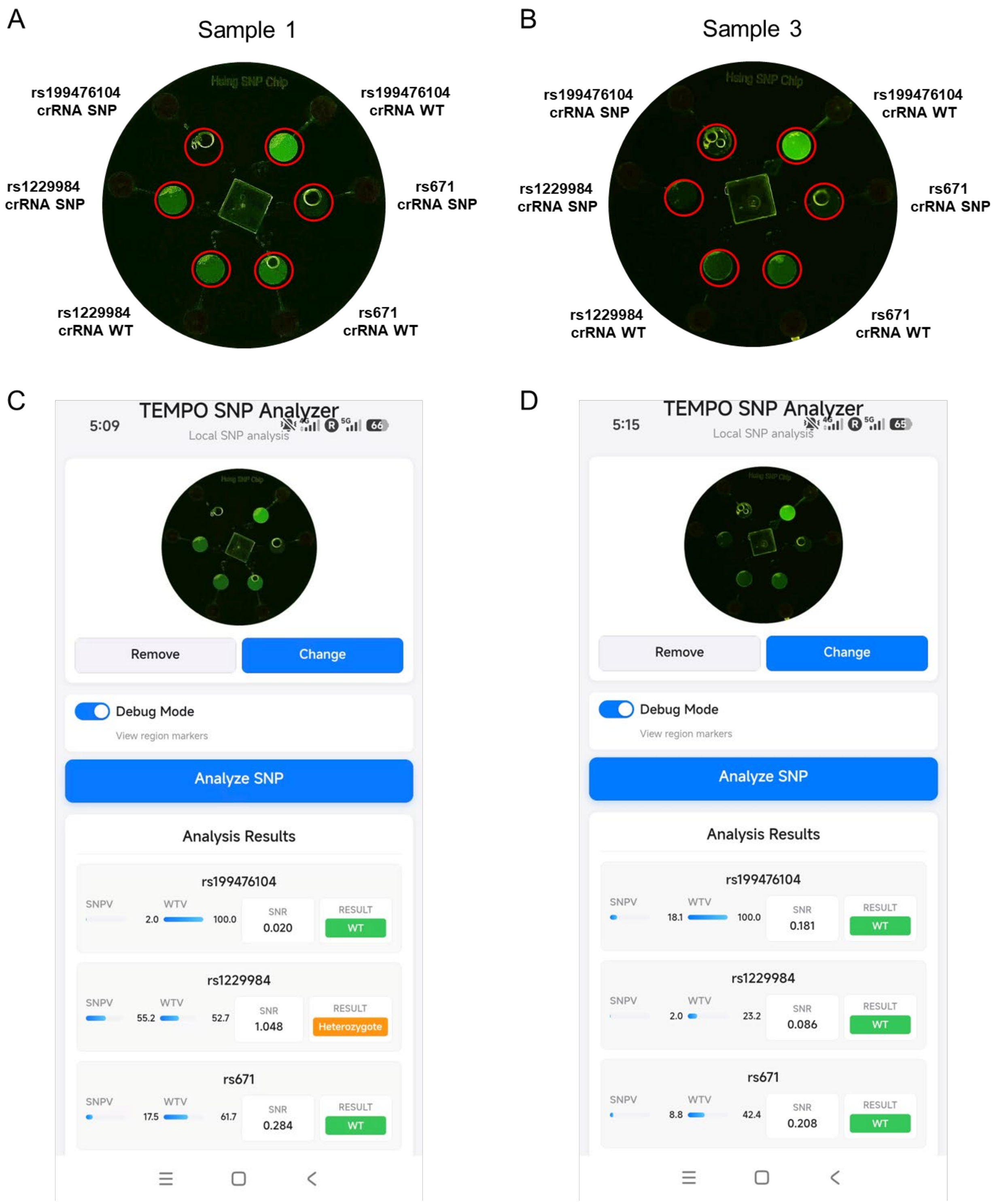

Supplementary Figure 23. Microfluidic chip–based fluorescence readout for representative samples.

Representative fluorescence images of the microfluidic SNP chip obtained using sample 1 (A) and sample 3 (B) after a 30 min reaction. (C and D) Smartphone application–based endpoint SNP

genotyping corresponding to the fluorescence images in (A) and (B). The mobile app automatically analyzed fluorescence intensities from individual reaction chambers and directly output genotype calls (WT, SNP, or heterozygous), enabling user-friendly, on-chip SNP discrimination.

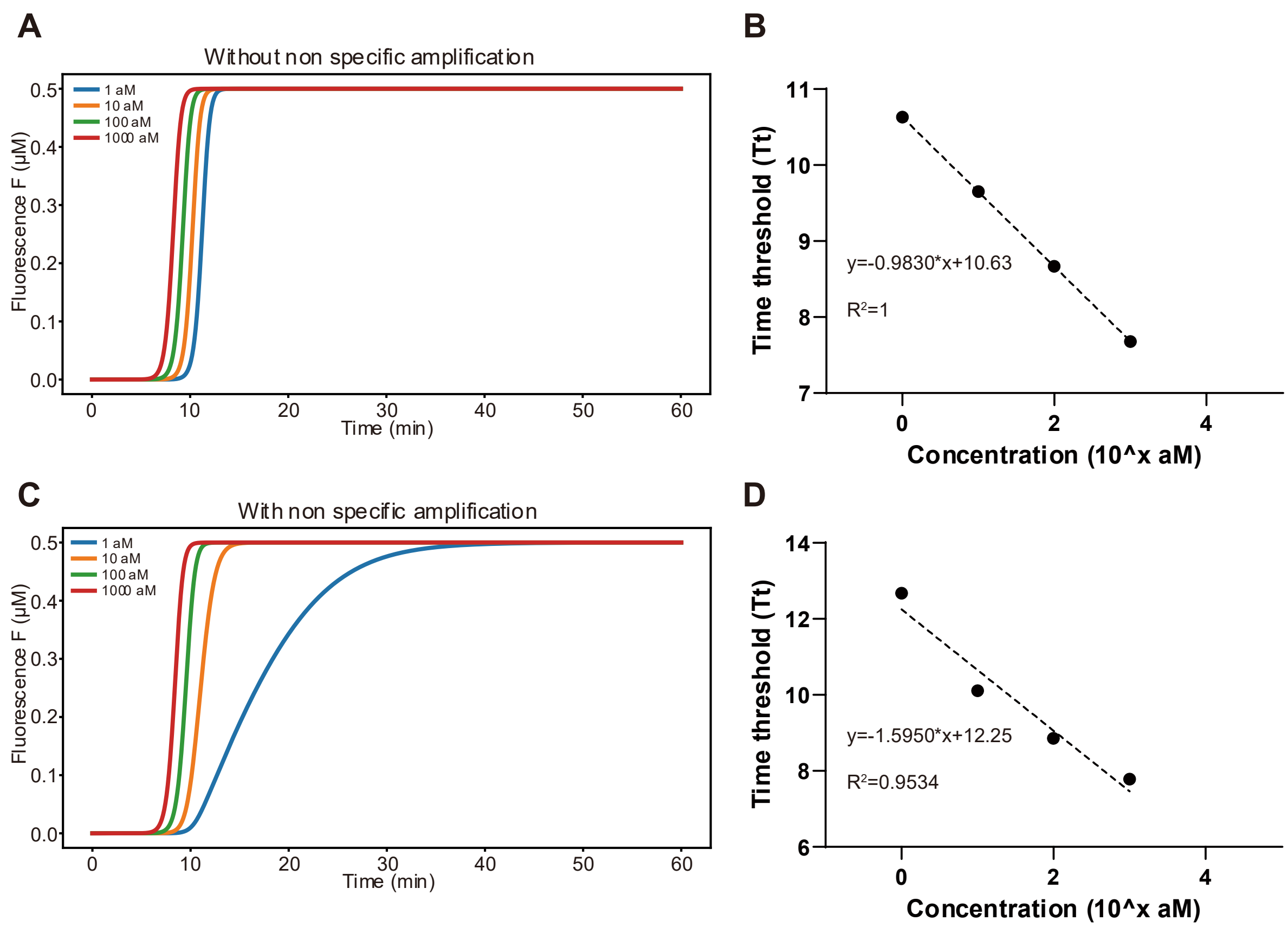

Supplementary Figure 24. ODE modelling of how nonspecific amplification influences quantitative performance.

(A) Simulated fluorescence generation kinetics for a gradient of template concentrations in the absence of nonspecific amplification ($k_{\text{ns\_init}} = 0$). **(B)** Corresponding standard curve for **A**, generated using the threshold time (Tt), defined as the time required for the fluorescence signal to reach a preset threshold. In the absence of nonspecific amplification, the simulated signal trajectories showed near-ideal behavior, with approximately equal Tt intervals between successive 10-fold changes in template concentration, consistent with quantitative detection performance ($R^2 = 1$). **(C)** Simulated fluorescence generation kinetics for a gradient of template concentrations in the presence of nonspecific amplification ($k_{\text{ns\_init}} = 1.5 \times 10^{-5}$). **(D)** Corresponding standard curve for **C**, generated using Tt values. Because nonspecific amplification is an intrinsic feature that must be considered in isothermal amplification, its inclusion in the model caused the Tt values at lower template concentrations to shift to earlier times relative to the ideal case. This compression of Tt spacing reduced the accuracy of template quantification, indicating impaired quantitative performance ($R^2 = 0.9534$).

Supplementary Table 1. Influenza A and SARS-CoV-2 clinical samples detection results by RT-qPCR.

Influenza A clinical samples		
**Sample No.**	**Ct**	**Result**
1	26.40	Positive
2	23.41	Positive
3	20.38	Positive
4	29.09	Positive
5	non	Negative
6	40.65	Negative
7	40.06	Negative
8	39.83	Negative
9	non	Negative
10	39.16	Negative
**SARS-CoV-2 clinical samples**		
**Sample No.**	**Ct**	**Result**
1	18.49	Positive
2	22.16	Positive
3	24.15	Positive
4	19.05	Positive
5	19.73	Positive
6	25.41	Positive
7	21.97	Positive
8	35.19	Negative
9	21.15	Positive
10	21.44	Positive
11	non	Negative
12	36.47	Negative
13	non	Negative
14	non	Negative
15	non	Negative
16	non	Negative
17	non	Negative
18	40.22	Negative
19	non	Negative
20	38.52	Negative
21	37.35	Negative
22	37.42	Negative

Supplementary Table 2. Composition of the TEMPO system.

Component	Conc	Final conc	Volume
LbCas12a*	1 μM	25 nM	0.5 μL
crRNA	2 μM	50 nM	0.5 μL
Reporter	10 μM	400 nM	0.8 μL
NEB buffer2.1	-	-	0.5 μL
Rehydration buffer A	-	-	7.5 μL
Primer-FP	10 μM	125 nM	0.25 μL
Primer-FPP	10 μM	125 nM	0.25 μL
Primer-BP	10 μM	250 nM	0.5 μL
DEPC H_2O	-	-	3.8 μL
Buffer B (MgOAc)**	280 mM	19.6 mM	1.4 μL
Target	-	-	4 μL
Total			20 μL

* The Cas12a–crRNA complex was preincubated at room temperature for 5 min prior to mixing with other reagents.

** MgOAc is added last to initiate amplification.

a. Final concentrations were calculated based on a total reaction volume of 20 μL.

Supplementary Table 3. Lyophilization formulation of the TEMPO system.

Component	Conc	Final conc	Volume
LbCas12a*	1 μM	50 nM	1 μL
crRNA	2 μM	100 nM	1 μL
Reporter	10 μM	800 nM	1.6 μL
NEB buffer2.1	-	-	0.5 μL
Primer-FP	10 μM	125 nM	0.25 μL
Primer-FPP	10 μM	125 nM	0.25 μL
Primer-BP	10 μM	250 nM	0.5 μL
DEPC H_2O*	-	-	10.9 μL
Sucrose	50%	5%	2 μL
Mannitol	20%	2%	2 μL
Total			20 μL

* The Cas12a–crRNA complex was preincubated at room temperature for 5 min prior to mixing with other reagents.

** Reconstitute RPA reagents with DEPC H_2O.

a. Final concentrations were calculated based on a total reaction volume of 20 μL.
b. Lyophilize for 12 hours in the benchtop freeze dryer according to the above formulation.
c. The resuspension solution contains the Buffer A reduction solution provided in the kit, B buffer (MgOAc), and the sample.

Supplementary Note 1: Ordinary Differential Equations (ODEs)

Below is a comprehensive summary of all reactants, products, and rate coefficients used in the reaction steps and ordinary differential equations (ODEs) describing the one-pot RPA–CRISPR/Cas system. In the current model, the engineered forward primer (FPP) generates a distinct antisense branch through explicit binding and extension intermediates, and three independent nonspecific amplification channels are further incorporated. Accordingly, the system now includes the original specific RPA pathway, the FPP-derived amplification pathway, explicit primer–template intermediate complexes, and three nonspecific seed species (NS1–NS3) together with their binding, extension, and dsDNA formation steps. The Cas protein–crRNA complex (C) can bind either D or D2 to form CD or CD2, respectively. Upon binding, CD and CD2 acquire trans-cleavage activity and catalyze reporter (R) degradation to generate the fluorescence signal (F), which is described using a Michaelis–Menten kinetic framework. In addition, activated Cas–crRNA–dsDNA complexes can also cleave primers (FP, BP, FPP), since they are single-stranded DNA substrates, albeit with lower catalytic efficiency than for the reporter.

Reactants and Products

The following table lists each reaction, its reactants, products, and the associated rate coefficient.

No.	Reaction	Rate Expression	Notes
1	FP + D → FP·D	r1 = k1 * FP * D	
2	FP·D → ST + D	r1e = k1_ext * FP_D	
3	BP + D → BP·D	r2 = k2 * BP * D	
4	BP·D → AT + D	r2e = k2_ext * BP_D	
5	BP + ST → BP·ST	r3 = k3 * BP * ST	
6	BP·ST → D	r3e = k3_ext * BP_ST	
7	FP + AT → FP·AT	r4 = k4 * FP * AT	
8	FP·AT → D	r4e = k4_ext * FP_AT	
9	ST + AT → D	r5 = k5 * ST * AT	
10	FPP + D → FPP·D	r6 = k6 * FPP * D	
11	FPP·D → ST2 + D	r6e = k6_ext * FPP_D	
12	FPP + AT → FPP·AT	r7 = k7 * FPP * AT	
13	FPP·AT → ST2 + AT	r7e = k7_ext * FPP_AT	
14	BP + ST2 → D2	r8 = k8 * BP * ST2	
15	BP + D2 → BP·D2	r9 = k9 * BP * D2	
16	BP·D2 → AT2 + D2	r9e = k9_ext * BP_D2	
17	FPP + D2 → FPP·D2	r10 = k10 * FPP * D2	
18	FPP·D2 → ST2 + D2	r10e = k10_ext * FPP_D2	
19	FPP + AT2 → D2	r11 = k11 * FPP * AT2	
20	ST2 + AT2 → D2	r12 = k12 * ST2 * AT2	
21	C + D → CD	r13 = k13 * C * D	k13 << k14
22	C + D2 → CD2	r14 = k14 * C * D2	k14 >> k13

23	FP + BP → NS1	rns1_init = k_ns1_init * FP * BP	nonspecific seed formation
24	FP + NS1 → FP·NS1	rns1_1 = k_ns1_bind_fp * FP * NS1	
25	FP · NS1 → NS1_ST + NS1	rns1_1e = k_ns1_ext_fp * FP_NS1	
26	BP + NS1 → BP·NS1	rns1_2 = k_ns1_bind_bp * BP * NS1	
27	BP · NS1 → NS1_AT + NS1	rns1_2e = k_ns1_ext_bp * BP_NS1	
28	NS1_ST + NS1_AT → NS1	rns1_3 = k_ns1_form * NS1_ST * NS1_AT	
29	FPP + BP → NS2	rns2_init = k_ns2_init * FPP * BP	nonspecific seed formation
30	FPP + NS2 → FPP·NS2	rns2_1 = k_ns2_bind_fpp * FPP * NS2	
31	FPP·NS2 → NS2_ST + NS2	rns2_1e = k_ns2_ext_fpp * FPP_NS2	
32	BP + NS2 → BP·NS2	rns2_2 = k_ns2_bind_bp * BP * NS2	
33	BP · NS2 → NS2_AT + NS2	rns2_2e = k_ns2_ext_bp * BP_NS2	
34	NS2_ST + NS2_AT → NS2	rns2_3 = k_ns2_form * NS2_ST * NS2_AT	
35	FP + FPP → NS3	rns3_init = k_ns3_init * FP * FPP	nonspecific seed formation
36	FP + NS3 → FP·NS3	rns3_1 = k_ns3_bind_fp * FP * NS3	
37	FP · NS3 → NS3_ST + NS3	rns3_1e = k_ns3_ext_fp * FP_NS3	
38	FPP + NS3 → FPP·NS3	rns3_2 = k_ns3_bind_fpp * FPP * NS3	
39	FPP·NS3 → NS3_AT + NS3	rns3_2e = k_ns3_ext_fpp * FPP_NS3	
40	NS3_ST + NS3_AT → NS3	rns3_3 = k_ns3_form * NS3_ST * NS3_AT	
41	CD + R → CD + F	V1 = (kcat1 * CD * R) / (Km1 + R)	
42	CD2 + R → CD2 + F	V2 = (kcat2 * CD2 * R) / (Km2 + R)	V2 >> V1
43	CD + FP → CD + FP1	V3 = (kcat3 * CD * FP) / (Km3 + FP)	
44	CD + BP → CD + BP1	V4 = (kcat3 * CD * BP) / (Km3 + BP)	
45	CD + FPP → CD + FPP1	V5 = (kcat3 * CD * FPP) / (Km3 + FPP)	

46	CD2 + FP → CD2 + FP1	V6 = (kcat4 * CD2 * FP) / (Km4 + FP)
47	CD2 + BP → CD2 + BP1	V7 = (kcat4 * CD2 * BP) / (Km4 + BP)
48	CD2 + FPP → CD2 + FPP1	V8 = (kcat4 * CD2 * FPP) / (Km4 + FPP)

kcat3 = kcat1 / 2 Km3 = Km1 * 2

kcat4 = kcat2 / 2 Km4 = Km2 * 2

Species (Reactants and Products)

The species included in the current reaction system and ODEs are:

• FP: Forward primer (original, fully complementary to AT).
• BP: Backward primer (complementary to ST).
• ST: Sense template (single-stranded DNA).
• AT: Anti-sense template (single-stranded DNA, complementary to ST).
• D: Original double-stranded DNA (ST·AT).
• FPP: Engineered forward primer.
• ST2: Extended sense template produced by FPP extension on D, AT, or D2.
• AT2: Extended anti-sense template produced by BP extension on D2.
• D2: New double-stranded DNA (ST2·AT2, produced via FPP and BP reactions).
• C: Cas12a–crRNA protein complex (free form).
• CD: Complex of Cas12a–crRNA bound to D (weak activation).
• CD2: Complex of Cas12a–crRNA bound to D2 (strong activation).
• R: DNA reporter molecule.
• F: Fluorescence signal generated upon reporter cleavage.
• FP·D, BP·D, BP·ST, FP·AT, FPP·D, FPP·AT, BP·D2, and FPP·D2: explicit primer–template binding intermediates in the specific amplification network.
• FP·NS3, FPP·NS3, NS3_ST, NS3_AT: intermediates and extension products in nonspecific channel 3.
• FPP·NS2, BP·NS2, NS2_ST, NS2_AT: intermediates and extension products in nonspecific channel 2.
• FP·NS1, BP·NS1, NS1_ST, NS1_AT: intermediates and extension products in nonspecific channel 1.
• NS1, NS2, NS3: three independent nonspecific seed species formed from FP/BP, FPP/BP, and FP/FPP initiation, respectively.

Rate Coefficients

The rate coefficients used in the current ODE model (units: $\mu M^{-1} \cdot s^{-1}$ for bimolecular binding, s^{-1} for unimolecular extension or catalytic turnover) are:

• k1–k12: specific amplification rate constants for primer binding, extension, and strand hybridization in the D/D2 network.
• k1_ext, k2_ext, k3_ext, k4_ext, k6_ext, k7_ext, k9_ext, k10_ext: extension rates for the explicit bound intermediates in the specific amplification network.
• k13: Cas12a binding to D (C + D → CD, weak binding, no PAM).
• k14: Cas12a binding to D2 (C + D2 → CD2, strong binding, PAM-dependent).
• k_ns1_init, k_ns2_init, k_ns3_init: initiation rates for the three nonspecific seed species.
• k_ns1_bind_fp, k_ns1_bind_bp, k_ns1_ext_fp, k_ns1_ext_bp, k_ns1_form: rates for nonspecific channel 1.
• k_ns2_bind_fpp, k_ns2_bind_bp, k_ns2_ext_fpp, k_ns2_ext_bp, k_ns2_form: rates for nonspecific channel 2.
• k_ns3_bind_fp, k_ns3_bind_fpp, k_ns3_ext_fp, k_ns3_ext_fpp, k_ns3_form: rates for nonspecific channel 3.
Michaelis–Menten catalytic parameters:
• kcat1, Km1: catalytic turnover and Michaelis constant for reporter cleavage by CD.
• kcat2, Km2: catalytic turnover and Michaelis constant for reporter cleavage by CD2 (≫ CD).
• kcat3, Km3: catalytic turnover and Michaelis constant for primer digestion by CD.
• kcat4, Km4: catalytic turnover and Michaelis constant for primer digestion by CD2.
• k_{14}: Cas12a binding to D2 (C + D2 → CD2, strong binding, PAM-dependent).
Michaelis–Menten catalytic parameters:

- **$kcat_1$, Km_1**: Catalytic turnover and Michaelis constant for reporter cleavage by CD.
- **$kcat_2$, Km_2**: Catalytic turnover and Michaelis constant for reporter cleavage by CD2 (≫ CD).
- **$kcat_3$, Km_3**: Catalytic turnover and Michaelis constant for primer digestion by CD.
- **$kcat_4$, Km_4**: Catalytic turnover and Michaelis constant for primer digestion by CD2.

Current default values are: k1 = k2 = k3 = k4 = k8 = k9 = k10 = k11 = 0.15, k5 = k12 = 1.0, k6 = k7 = 0.1, k13 = 5×10^{-4}, k14 = 1.0, k1_ext = k2_ext = k3_ext = k4_ext = k6_ext = k7_ext = k9_ext = k10_ext = 1.0, kcat1 = 0.038, Km1 = 3.7, kcat2 = 0.38, Km2 = 0.37, k_ns1_init = k_ns2_init = k_ns3_init = 1.5×10^{-5}, all nonspecific binding rates = 0.15, all nonspecific extension rates = 1.0, and all nonspecific ds-formation rates = 1.0.
ODE System
This document summarizes the current ODE system describing the double-track RPA–CRISPR/Cas system with explicit bound intermediates and three independent nonspecific amplification channels.

Species	Differential Equation	Explanation
**FP (Forward primer)**	d[FP]/dt = -r1 - r4 - V3 - V6 - rns1_init - rns3_init - rns1_1 - rns3_1	FP is consumed by specific amplification, primer cleavage, nonspecific initiation, and nonspecific binding in channels 1 and 3.

**BP (Backward primer)**	d[BP]/dt = -r2 - r3 - r8 - r9 - V4 - V7 - rns1_init - rns2_init - rns1_2 - rns2_2	BP is consumed by specific amplification, primer cleavage, nonspecific initiation, and nonspecific binding in channels 1 and 2.
**FPP (Engineered forward primer)**	d[FPP]/dt = -r6 - r7 - r10 - r11 - V5 - V8 - rns2_init - rns3_init - rns2_1 - rns3_2	FPP is consumed by the FPP-specific branch, primer cleavage, nonspecific initiation, and nonspecific binding in channels 2 and 3.
**FP·D**	d[FP_D]/dt = +r1 - r1e	Binding intermediate for FP on D.
**FPP·D**	d[FPP_D]/dt = +r6 - r6e	Binding intermediate for FPP on D.
**BP·D**	d[BP_D]/dt = +r2 - r2e	Binding intermediate for BP on D.
**BP·ST**	d[BP_ST]/dt = +r3 - r3e	Binding intermediate for BP on ST.
**FP·AT**	d[FP_AT]/dt = +r4 - r4e	Binding intermediate for FP on AT.
**FPP·AT**	d[FPP_AT]/dt = +r7 - r7e	Binding intermediate for FPP on AT.
**ST**	d[ST]/dt = +r1e - r3 - r5	ST is produced by FP extension on D and consumed by BP binding and hybridization.
**AT**	d[AT]/dt = +r2e - r4 - r5	AT is produced by BP extension on D and consumed by FP binding and hybridization.
**ST2**	d[ST2]/dt = +r6e + r7e + r10e - r8 - r12	ST2 is generated from FPP extension and consumed to form D2.
**AT2**	d[AT2]/dt = +r9e - r11 - r12	AT2 is generated from BP extension on D2 and consumed to form D2.
**D (Original dsDNA)**	d[D]/dt = +r3e + r4e + r5 - r13 - r1 - r6 + r1e + r6e	D is regenerated through extension/hybridization, consumed by Cas binding and

		primer binding, and restored after extension.
**D2 (New dsDNA)**	d[D2]/dt = +r8 + r11 + r12 - r14 - r9 - r10 + r9e + r10e	D2 is generated by the FPP branch, consumed by Cas binding and primer binding, and restored after extension.
**C (Cas12a–crRNA)**	d[C]/dt = -r13 - r14	Free Cas12a–crRNA is consumed by binding D and D2.
**CD**	d[CD]/dt = +r13	Cas12a bound to D.
**CD2**	d[CD2]/dt = +r14	Cas12a bound to D2.
**R (Reporter)**	d[R]/dt = -V1 - V2	Reporter is consumed by Cas-mediated cleavage.
**F (Fluorescence)**	d[F]/dt = +V1 + V2	Fluorescence accumulates as reporter is cleaved.
**BP·D2**	d[BP_D2]/dt = +r9 - r9e	Binding intermediate for BP on D2.
**FPP·D2**	d[FPP_D2]/dt = +r10 - r10e	Binding intermediate for FPP on D2.
**NS1**	d[NS1]/dt = +rns1_init + rns1_1e + rns1_2e + rns1_3 - rns1_1 - rns1_2	Nonspecific seed species for the FP/BP channel.
**FP·NS1**	d[FP_NS1]/dt = +rns1_1 - rns1_1e	Intermediates and extension products in nonspecific channel 1.
**BP·NS1**	d[BP_NS1]/dt = +rns1_2 - rns1_2e	Intermediates and extension products in nonspecific channel 1.
**NS1_ST**	d[NS1_ST]/dt = +rns1_1e - rns1_3	Intermediates and extension products in nonspecific channel 1.
**NS1_AT**	d[NS1_AT]/dt = +rns1_2e - rns1_3	Intermediates and extension products in nonspecific channel 1.
**NS2**	d[NS2]/dt = +rns2_init + rns2_1e + rns2_2e + rns2_3 - rns2_1 - rns2_2	Nonspecific seed species for the FPP/BP channel.

**FPP·NS2**	d[FPP_NS2]/dt = +rns2_1 - rns2_1e	Intermediates and extension products in nonspecific channel 2.
**BP·NS2**	d[BP_NS2]/dt = +rns2_2 - rns2_2e	Intermediates and extension products in nonspecific channel 2.
**NS2_ST**	d[NS2_ST]/dt = +rns2_1e - rns2_3	Intermediates and extension products in nonspecific channel 2.
**NS2_AT**	d[NS2_AT]/dt = +rns2_2e - rns2_3	Intermediates and extension products in nonspecific channel 2.
**NS3**	d[NS3]/dt = +rns3_init + rns3_1e + rns3_2e + rns3_3 - rns3_1 - rns3_2	Nonspecific seed species for the FP/FPP channel.
**FP·NS3**	d[FP_NS3]/dt = +rns3_1 - rns3_1e	Intermediates and extension products in nonspecific channel 3.
**FPP·NS3**	d[FPP_NS3]/dt = +rns3_2 - rns3_2e	Intermediates and extension products in nonspecific channel 3.
**NS3_ST**	d[NS3_ST]/dt = +rns3_1e - rns3_3	Intermediates and extension products in nonspecific channel 3.
**NS3_AT**	d[NS3_AT]/dt = +rns3_2e - rns3_3	Intermediates and extension products in nonspecific channel 3.

Catalytic Terms (Michaelis–Menten kinetics)
V1 = (kcat1 * [CD] * [R]) / (Km1 + [R])   (Reporter cleavage by CD)
V2 = (kcat2 * [CD2] * [R]) / (Km2 + [R])   (Reporter cleavage by CD2)
V3 = (kcat3 * [CD] * [FP]) / (Km3 + [FP]) (FP cleavage by CD)
V4 = (kcat3 * [CD] * [BP]) / (Km3 + [BP]) (BP cleavage by CD)
V5 = (kcat3 * [CD] * [FPP]) / (Km3 + [FPP]) (FPP cleavage by CD)
V6 = (kcat4 * [CD2] * [FP]) / (Km4 + [FP]) (FP cleavage by CD2)
V7 = (kcat4 * [CD2] * [BP]) / (Km4 + [BP]) (BP cleavage by CD2)
V8 = (kcat4 * [CD2] * [FPP]) / (Km4 + [FPP]) (FPP cleavage by CD2)
with a small numerical offset (+ 1e-16) included in the code denominator for numerical stability.
Qualitative interpretation of key kinetic parameters in the TEMPO model

Primer binding asymmetry (k1 versus k6).

k1 describes binding of the fully matched forward primer (FP) to the original template, whereas k6 describes binding of the engineered forward primer (FPP). Because FP is fully complementary to its target, its binding is more favorable and proceeds more readily. By contrast, FPP contains intentionally introduced mismatches, which reduce hybridization efficiency and slow the initiation of the FPP-dependent branch. This difference is a key design principle of TEMPO: the original amplification pathway proceeds first, while the PAM-installing pathway is delayed. In physical terms, the relative magnitude of k1 and k6 determines how long the reaction remains in a pre-activation amplification phase before PAM-containing products begin to accumulate.

PAM-dependent Cas12a target recognition (k13 versus k14).

k13 and k14 describe Cas12a–crRNA binding to the two classes of amplicons generated during the reaction. k13 corresponds to binding of the original amplicon D, which lacks a PAM and therefore interacts only weakly with Cas12a. k14 corresponds to binding of the engineered amplicon D2, which contains the introduced PAM and is therefore recognized much more efficiently. This large difference provides the gating mechanism of TEMPO: amplification can proceed initially without substantial CRISPR activation, and only after sufficient D2 has been produced does Cas12a binding rise sharply.

Trans-cleavage efficiency after target binding (kcat1/Km1 versus kcat2/Km2).

Once Cas12a binds its target, the resulting complex can cleave the fluorescent single-stranded DNA reporter in trans. The parameters kcat1 and Km1 describe reporter cleavage by the weakly activated complex CD, whereas kcat2 and Km2 describe reporter cleavage by the strongly activated complex CD2. In the model, CD has much lower catalytic efficiency than CD2. As a result, PAM-free amplicons contribute little to fluorescence generation, whereas PAM-containing amplicons trigger rapid reporter cleavage and a strong signal increase. This difference explains why the experimental fluorescence output remains low during the early phase and then rises abruptly once the PAM-dependent branch becomes dominant.

Collateral primer degradation by activated Cas12a (kcat3–4, Km3–4).

In addition to reporter cleavage, activated Cas12a can also cleave other single-stranded DNA molecules present in the reaction, including the primers FP, FPP, and BP. This process is represented by kcat3, Km3, kcat4, and Km4, which are assigned lower efficiency than reporter cleavage to reflect the lower substrate preference of Cas12a for primers relative to the reporter. Biologically, this introduces a delayed negative feedback effect: once Cas12a becomes activated, it gradually depletes the primer pool and thereby slows further amplification. This effect helps limit late-stage amplification and contributes to the eventual leveling-off of the fluorescence signal.

Rehybridization of amplification intermediates (k5 and k12).

During amplification, single-stranded intermediates can re-anneal to form double-stranded products. k5 describes rehybridization of the original strands (ST and AT) to regenerate D, while k12 describes rehybridization of the engineered strands (ST2 and AT2) to form D2. These reactions represent a background strand-pairing process that occurs in parallel with primer-driven

extension. In kinetic terms, they help shape the balance between free single-stranded intermediates and amplifiable double-stranded products, and thus influence how quickly each amplification branch progresses.

Non-specific amplification parameters.

In the model, additional parameters are included to describe three independent non-specific amplification channels. These terms represent unintended primer–primer or off-target interactions that can still consume primers and generate background products even in the absence of the intended amplification route. Although these channels do not directly activate the strongest CRISPR response in the same way as the PAM-containing specific product, they compete for shared reaction resources and therefore affect the overall reaction efficiency, signal onset, and background kinetics. Including these terms makes the model better reflect the experimentally observed trade-off between productive amplification and non-specific consumption of primers.

Together, these parameters define a hierarchical kinetic framework in which reaction order emerges from molecular design. Faster amplification of the original branch precedes slower PAM-installing amplification, which in turn enables strong PAM-dependent Cas12a activation. At the same time, collateral primer cleavage and non-specific amplification provide competing and self-limiting effects that shape the late-phase behavior of the system. This kinetic hierarchy explains how TEMPO can autonomously separate amplification from CRISPR activation within a single reaction mixture.

Supplementary References

1. Kottur, J. & Nair, D. T. Pyrophosphate hydrolysis is an intrinsic and critical step of the DNA synthesis reaction. Nucleic Acids Res. 46, 5875–5885 (2018).
2. Huyke, D. A. *et al.* Enzyme Kinetics and Detector Sensitivity Determine Limits of Detection of Amplification-Free CRISPR-Cas12 and CRISPR-Cas13 Diagnostics. *Anal. Chem.* **94**, 9826–9834 (2022).

Uncropped gel images

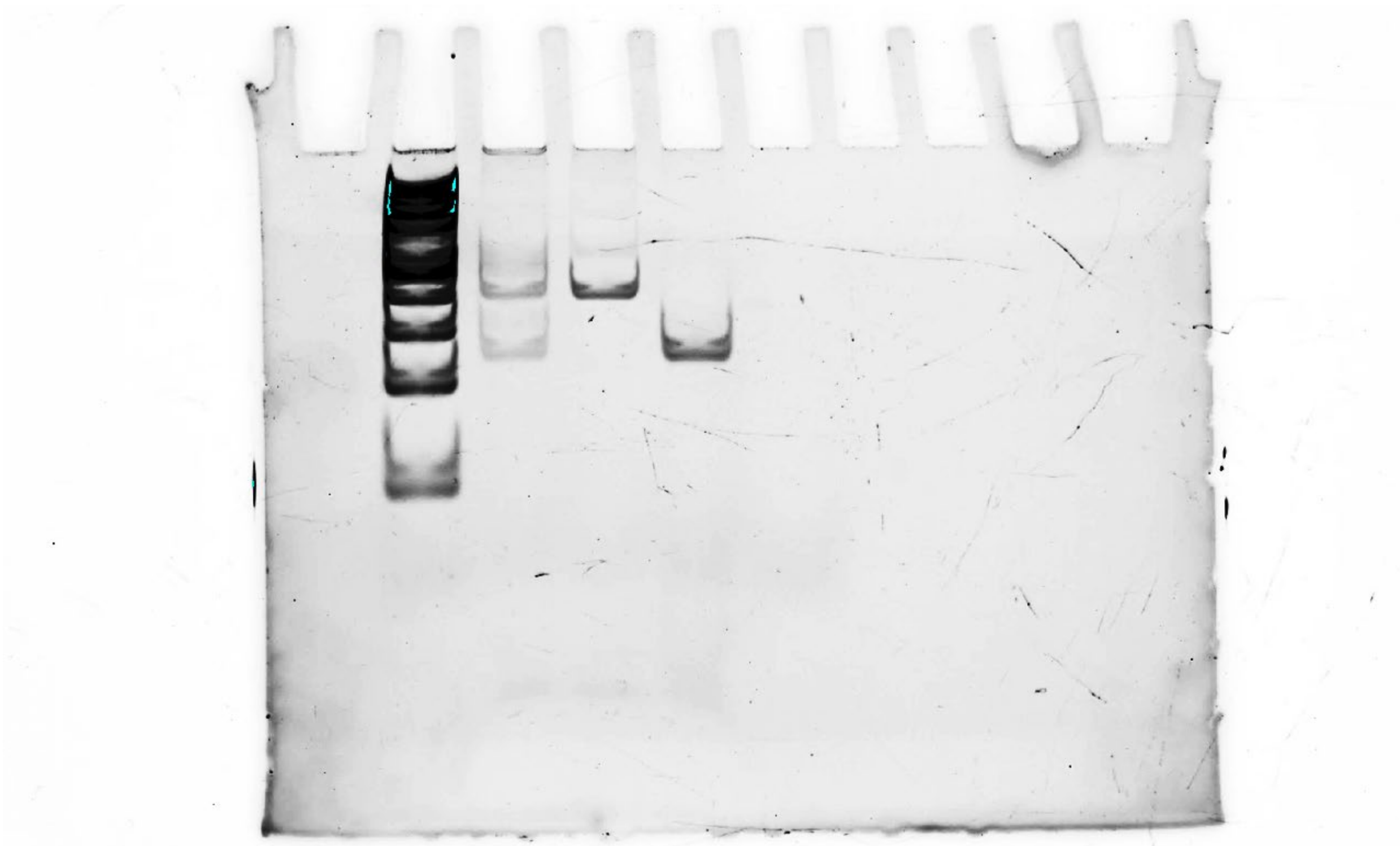

Uncropped gel images corresponding to Supplementary Figure 2A.

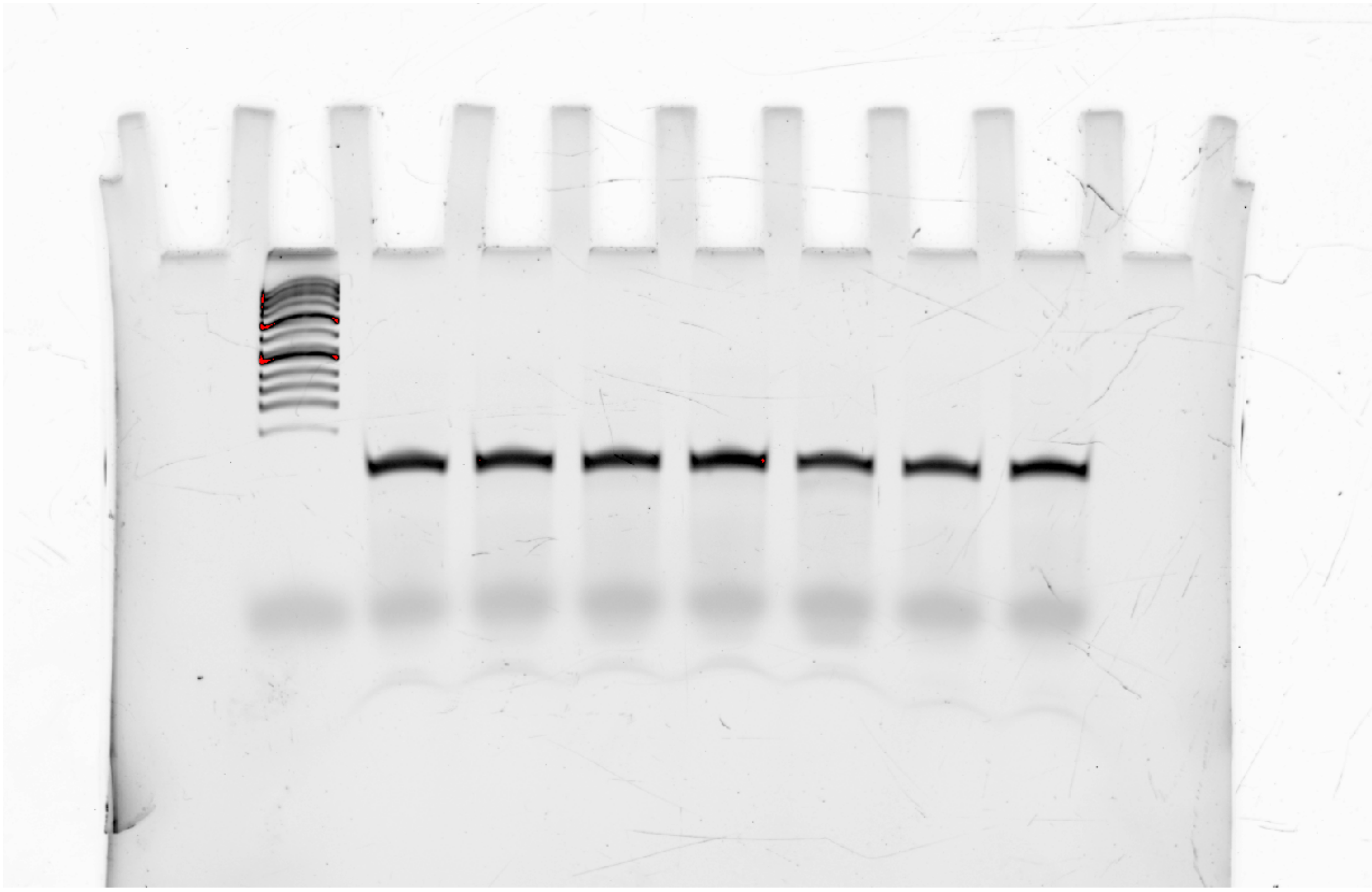

Uncropped gel images corresponding to Supplementary Figure 7A.

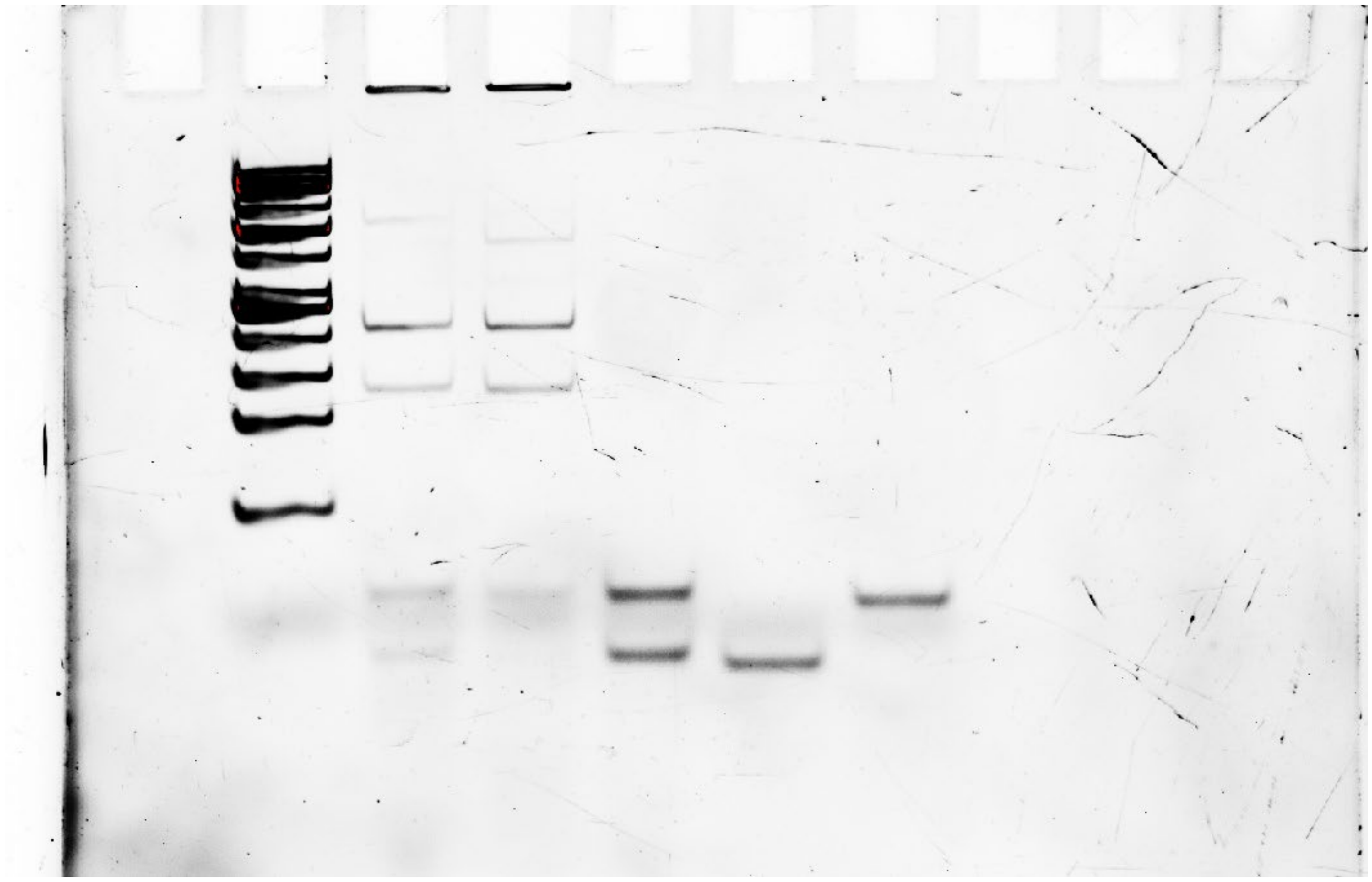

Uncropped gel images corresponding to Supplementary Figure 7C.
