# Supplementary material for: Thermodynamically programmed one-pot CRISPR platform for point-of-care SNP genotyping
Source: Nat Commun. 2026 Jul 7;17:8376. doi: 10.1038/s41467-026-75358-1 (PMC13473493; doi:10.1038/s41467-026-75358-1)
Supplement: Supplementary file 2 [file 41467_2026_75358_MOESM2_ESM.pdf]

Description of Additional Supplementary Files

File Name: Supplementary Data 1
Description: TEMPObased detection and Sanger sequence result for human samples.

File Name: Supplementary Data 2
Description: Nucleic acid sequences used in this study.

File Name: Supplementary Data 3
Description: Sanger sequencing raw Data generated in this study.
