# Supplementary material for: Thermodynamically programmed one-pot CRISPR platform for point-of-care SNP genotyping
Source: Nat Commun. 2026 Jul 7;17:8376. doi: 10.1038/s41467-026-75358-1 (PMC13473493; doi:10.1038/s41467-026-75358-1)
Supplement: Supplementary file 3 [file 41467_2026_75358_MOESM3_ESM.pdf]

TEMPO-based detection and Sanger sequence result for human samples

No.	rs1229984	rs671	rs199476104	TEMPO-based result
1	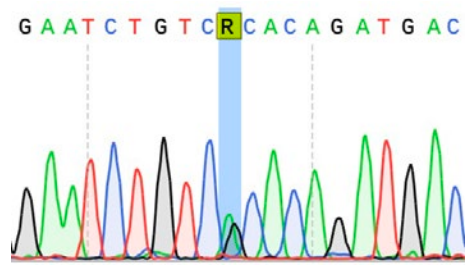	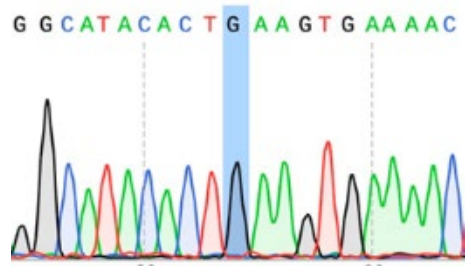	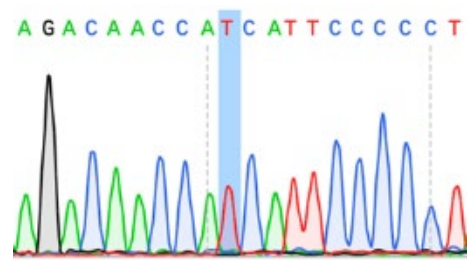	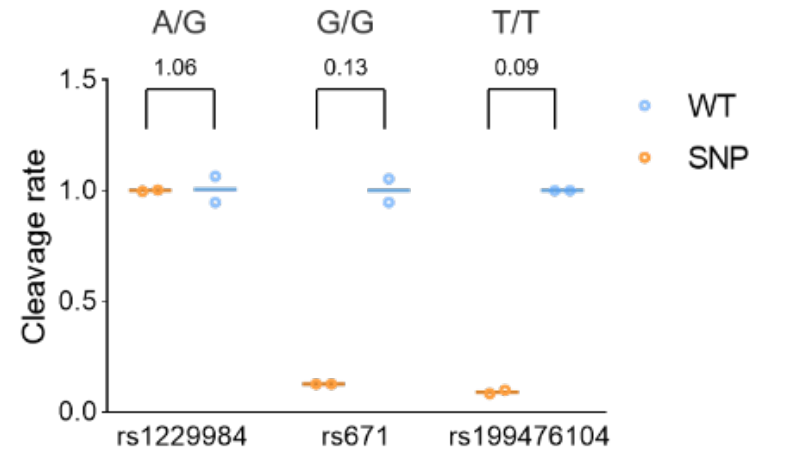
2	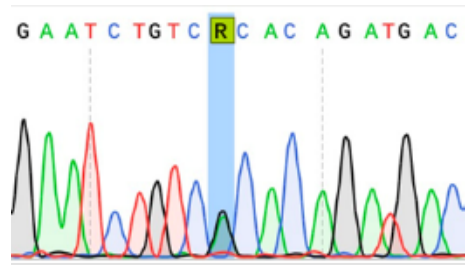	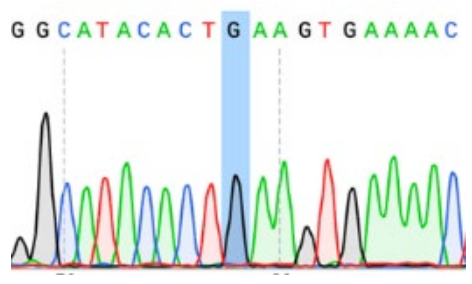	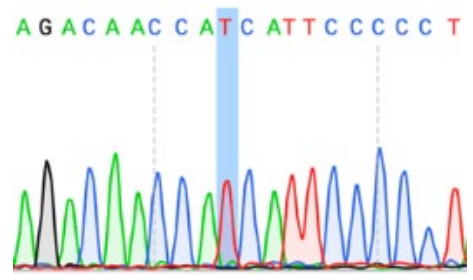	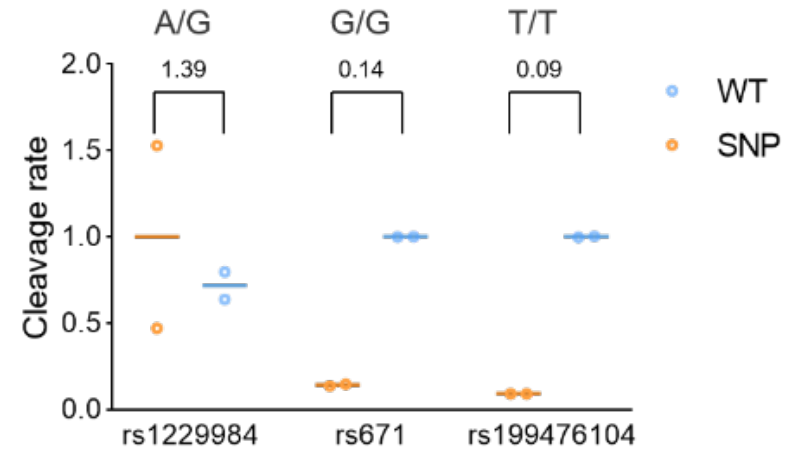
3	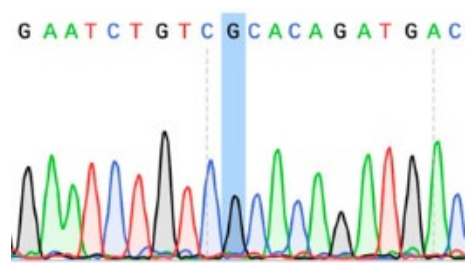	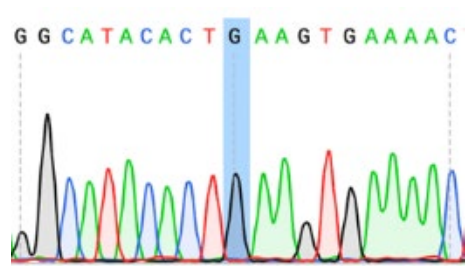	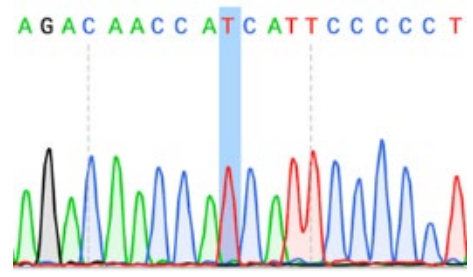	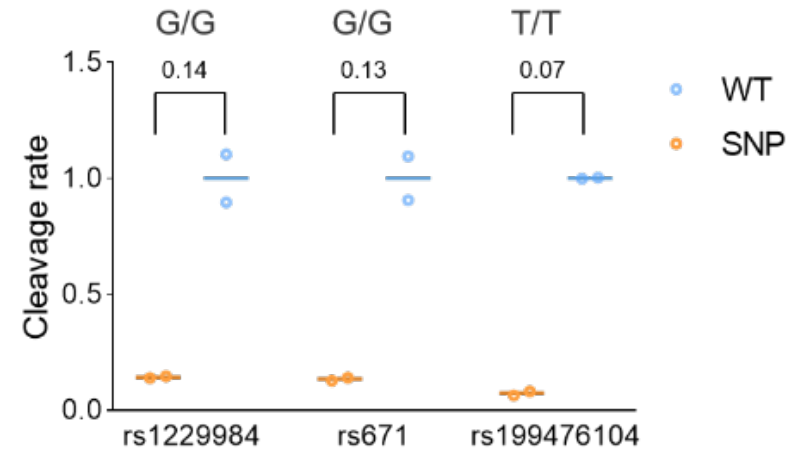
4	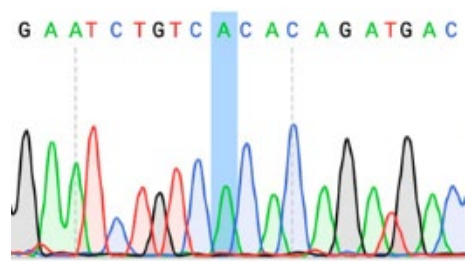	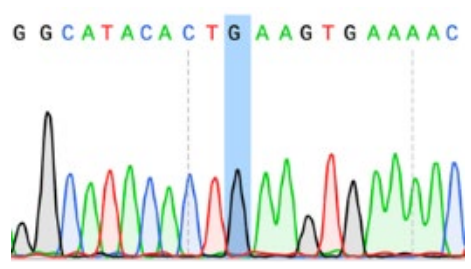	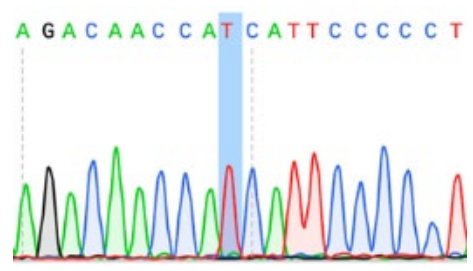	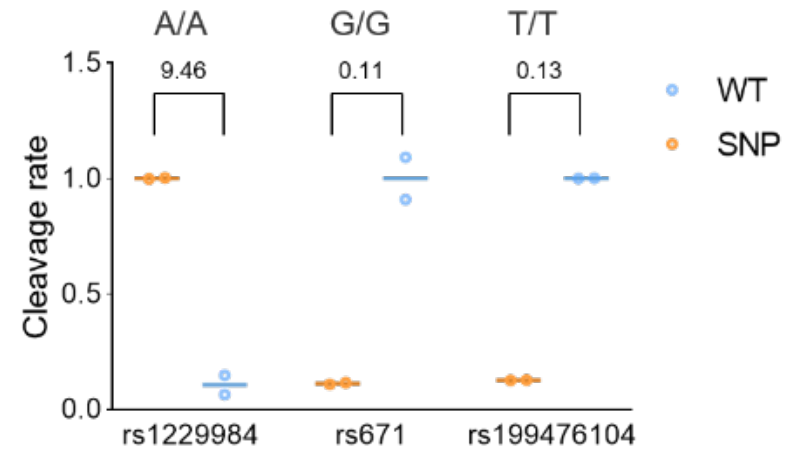
5	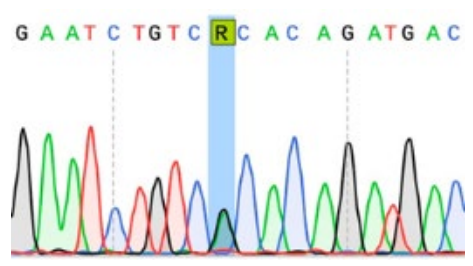	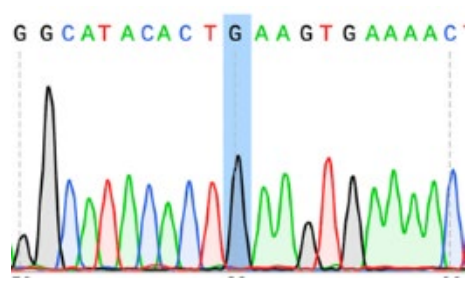	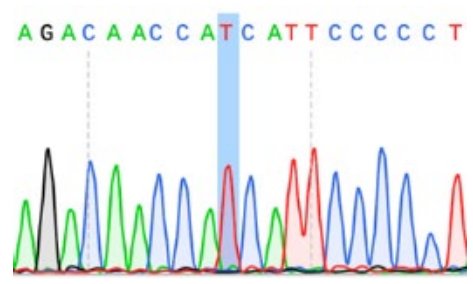	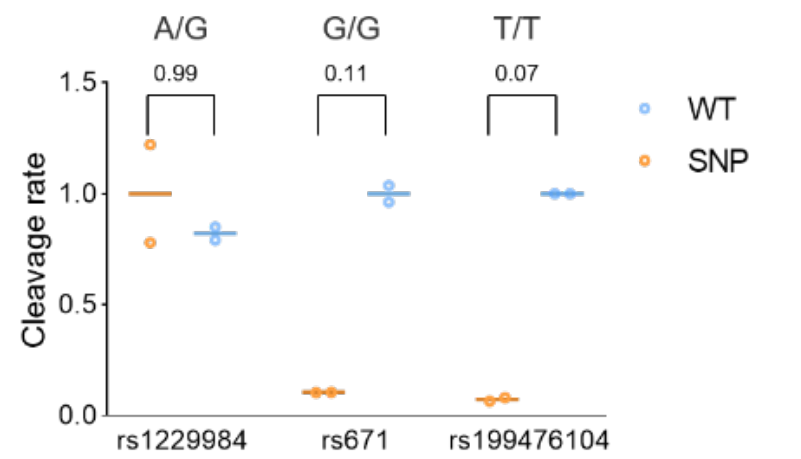
6	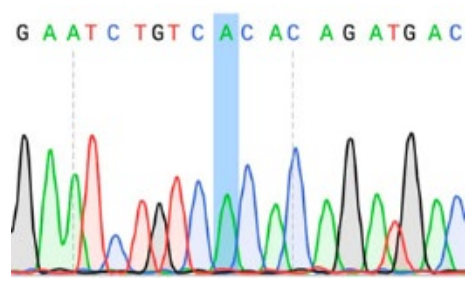	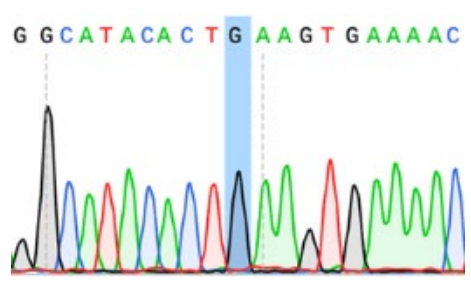	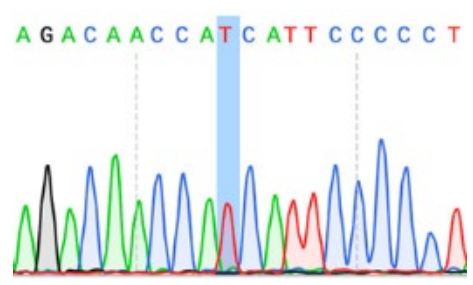	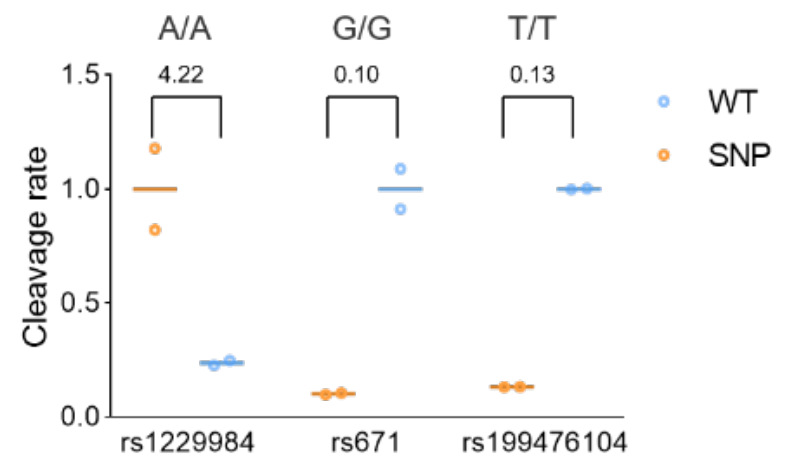

7	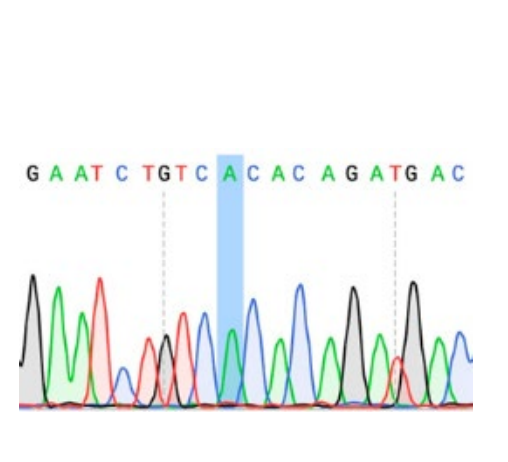	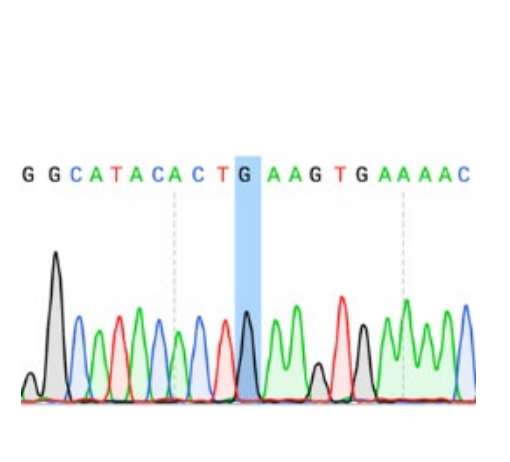	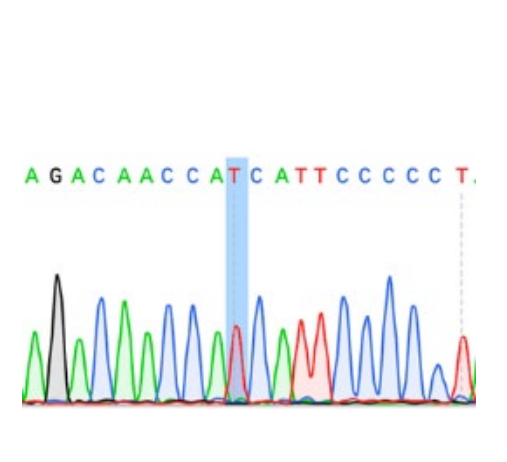	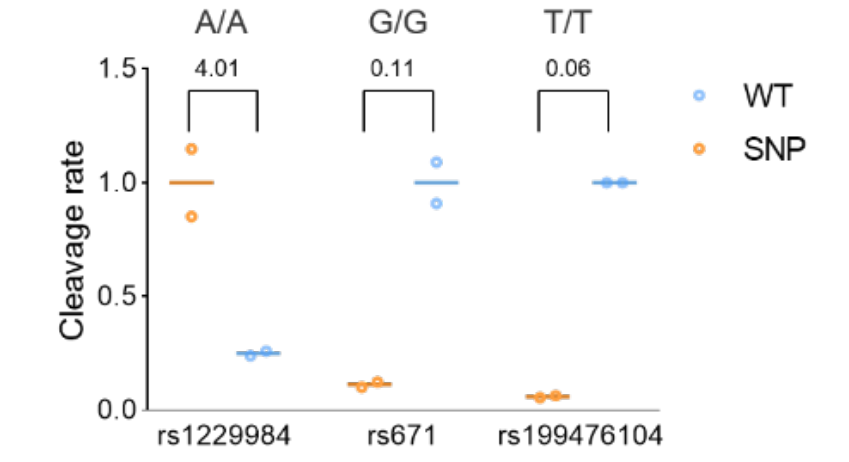
8	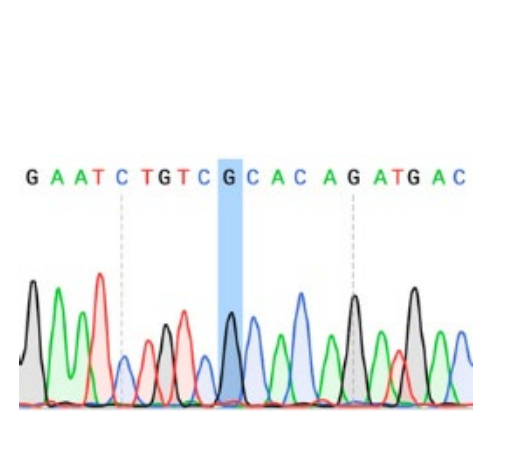	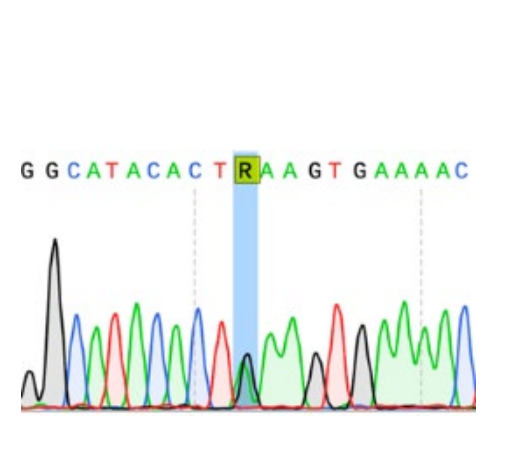	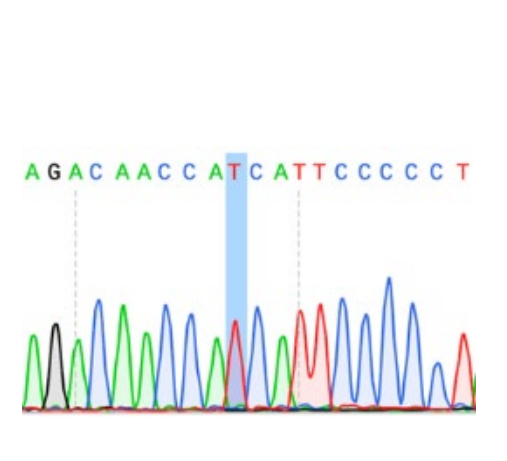	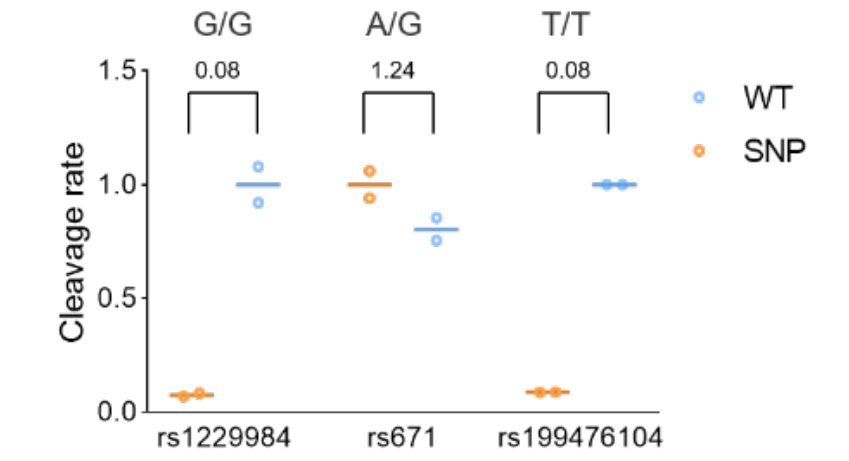
9	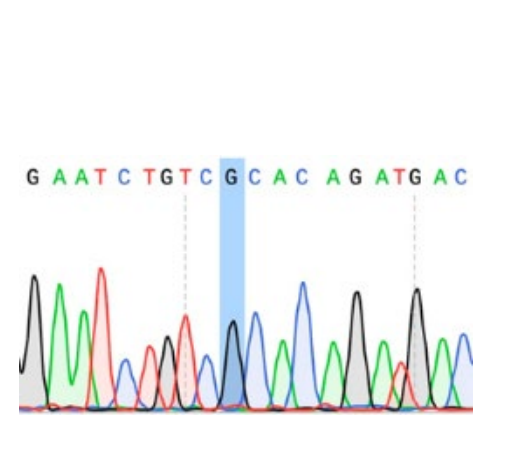	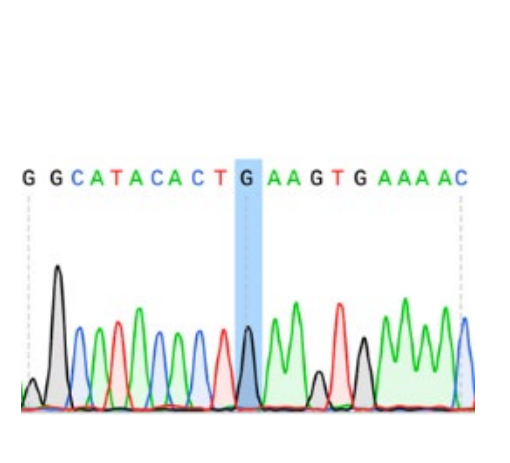	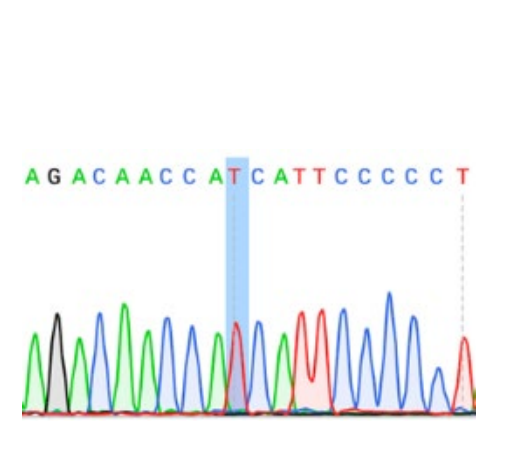	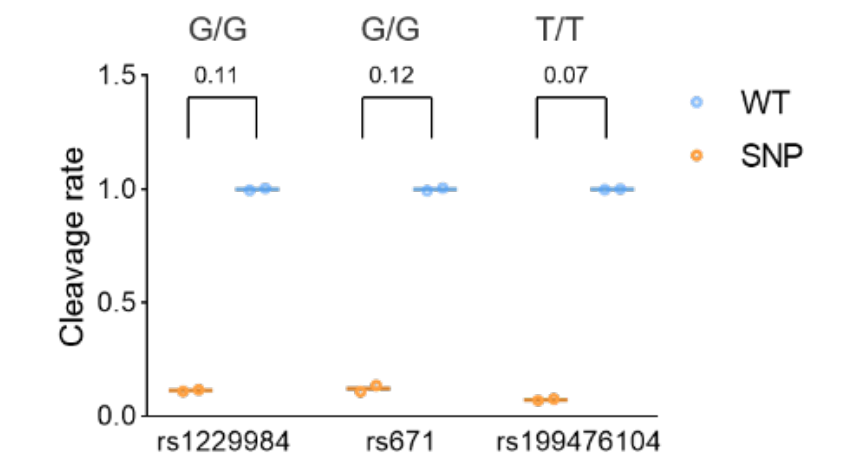
10	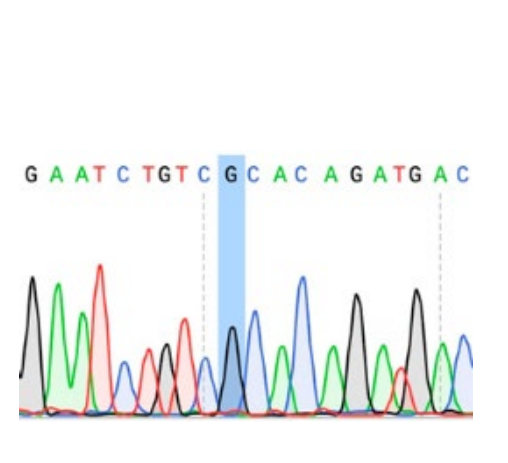	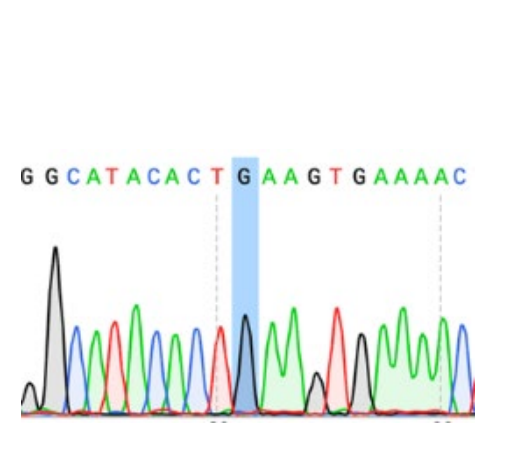	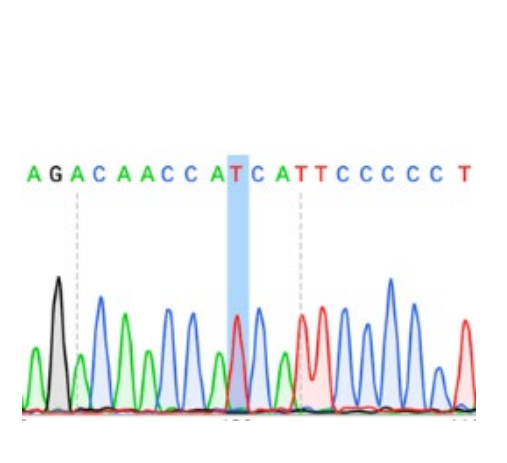	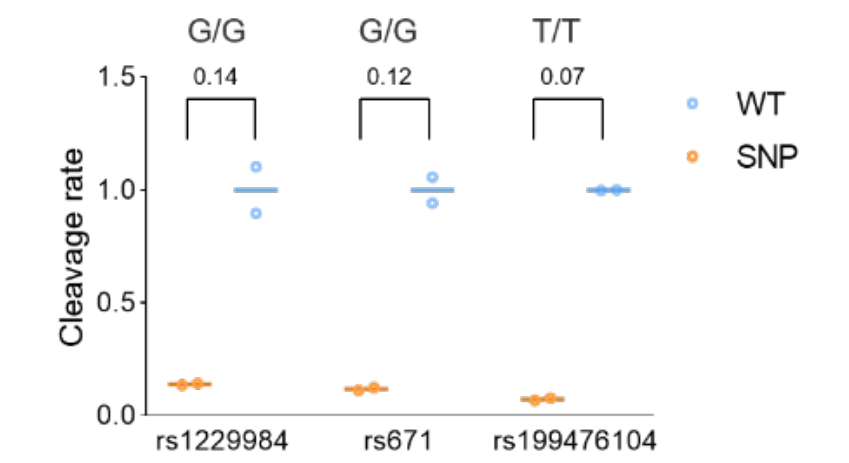
11	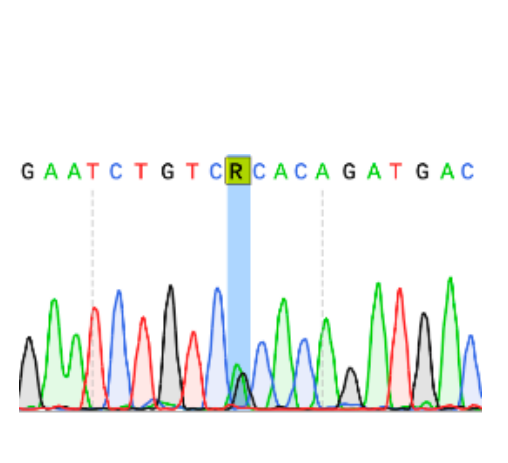	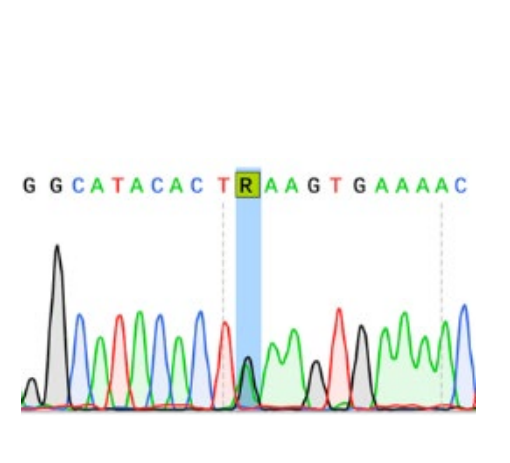	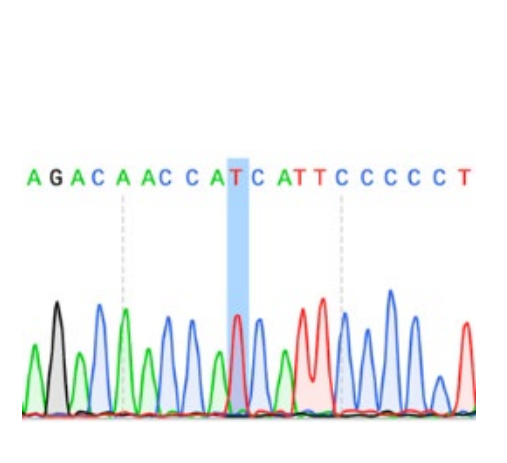	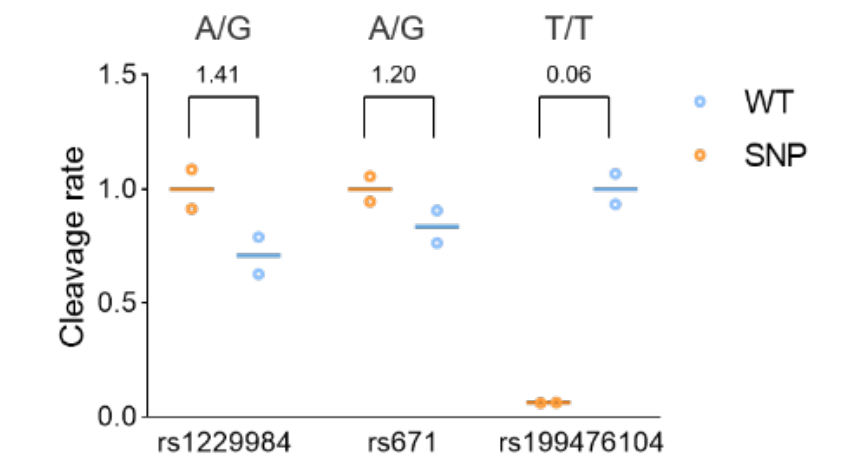
12	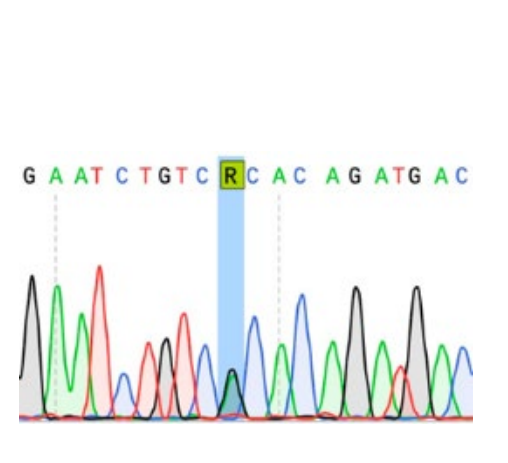	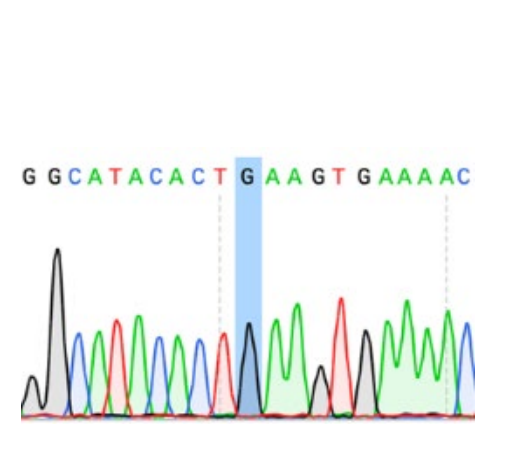	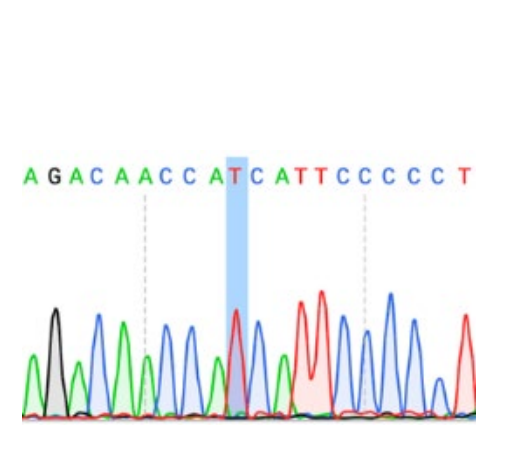	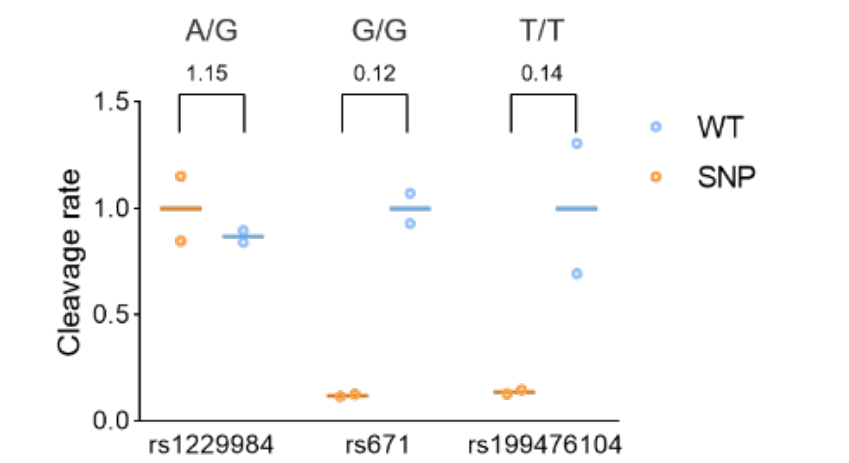
13	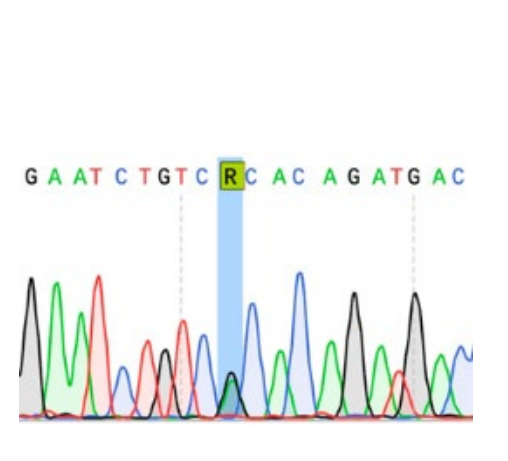	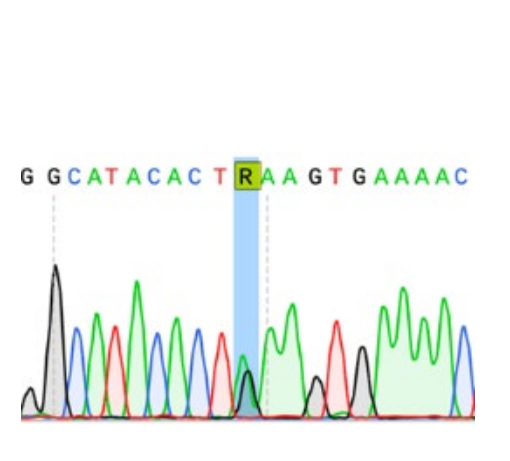	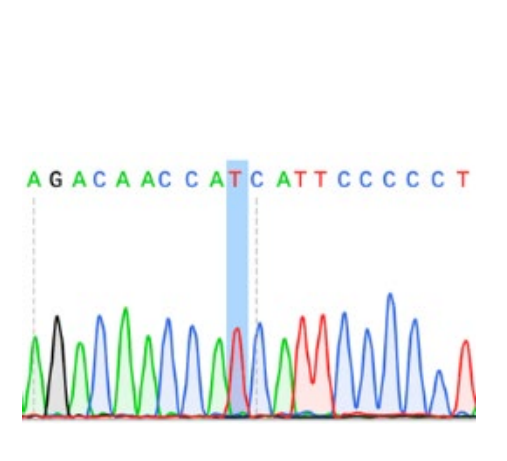	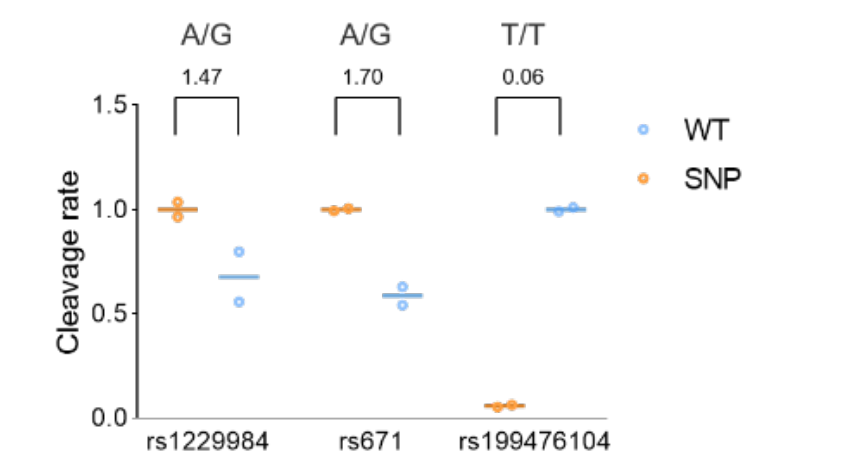

14	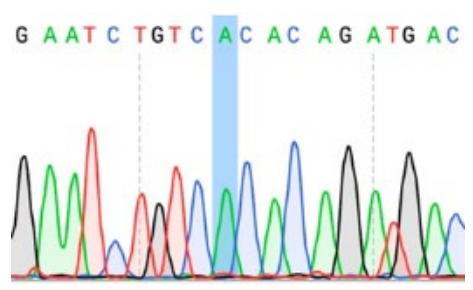	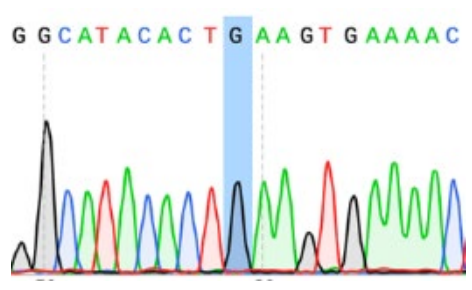	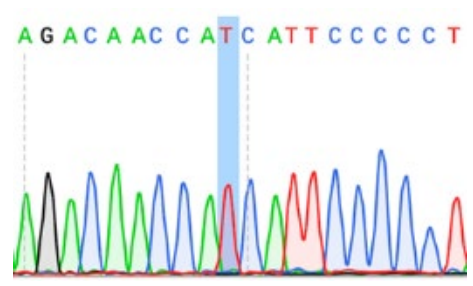	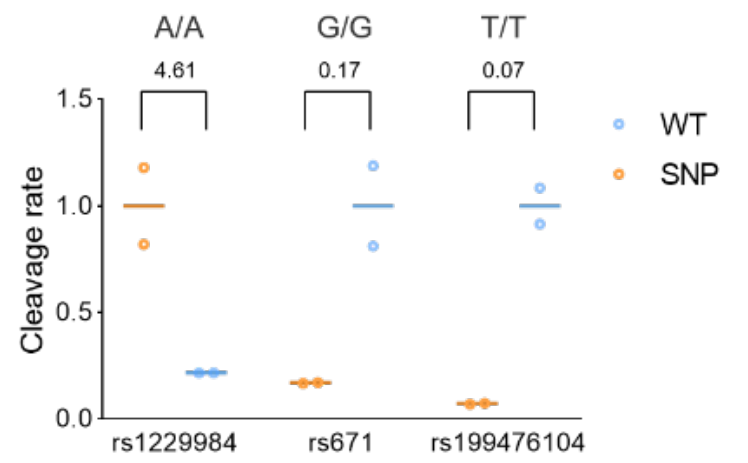
15	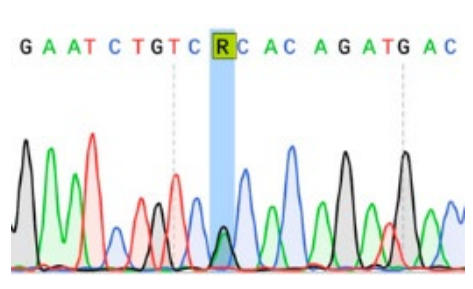	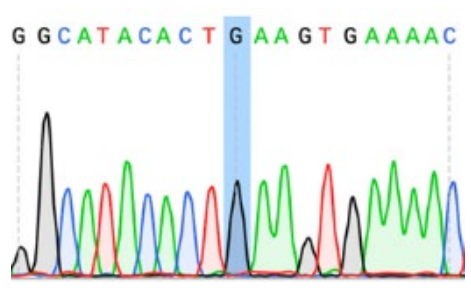	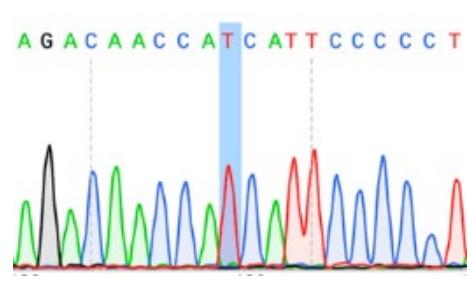	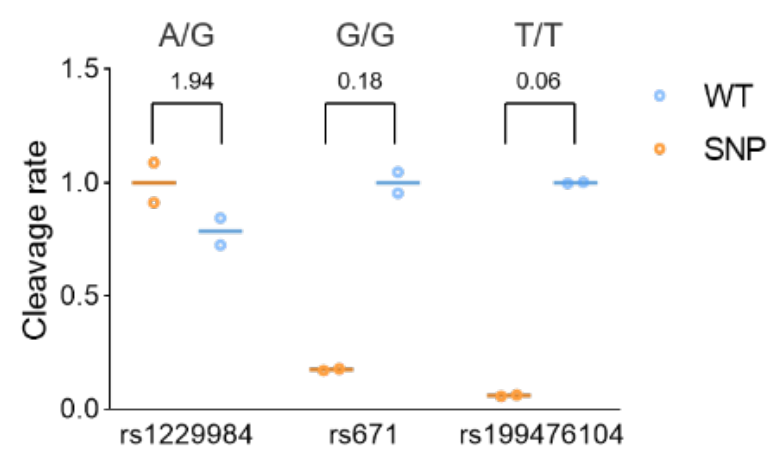
16	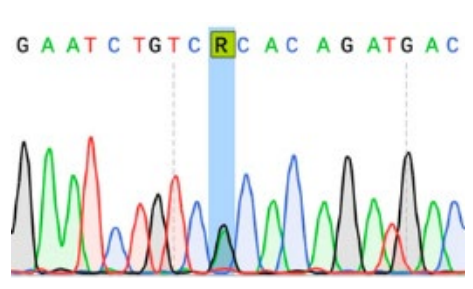	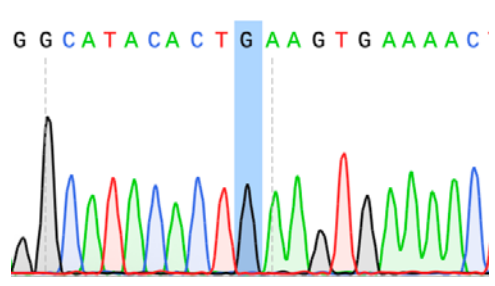	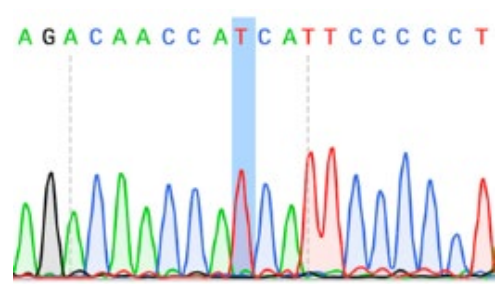	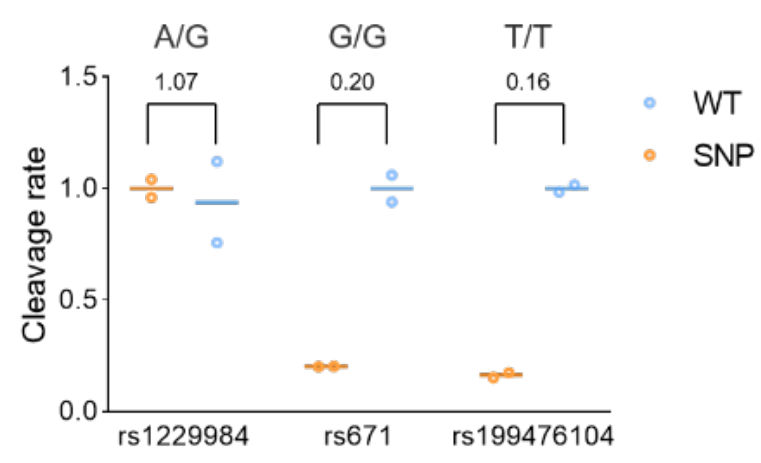
17	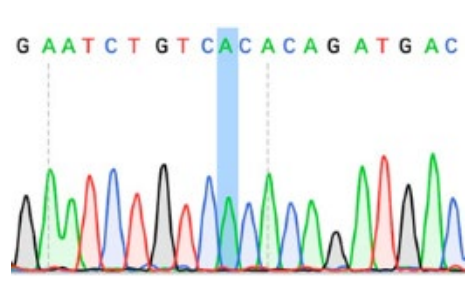	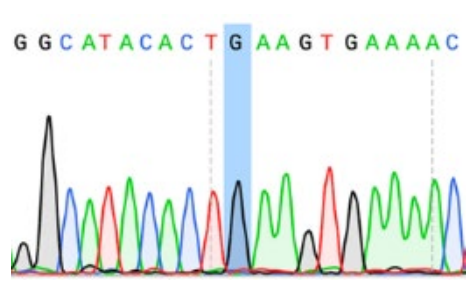	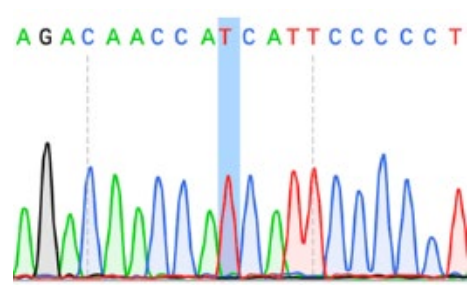	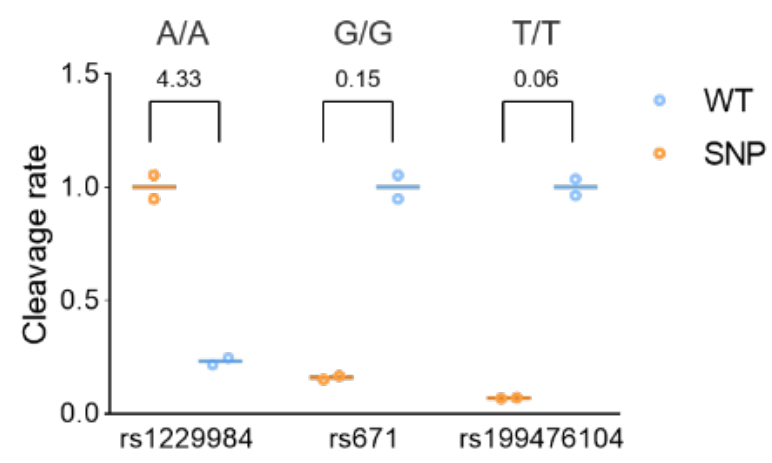
18	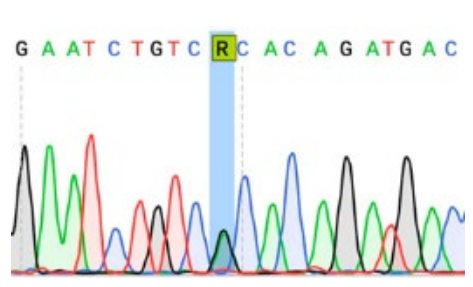	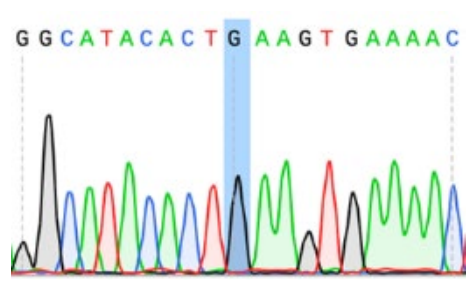	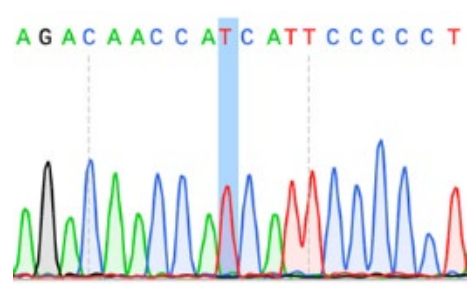	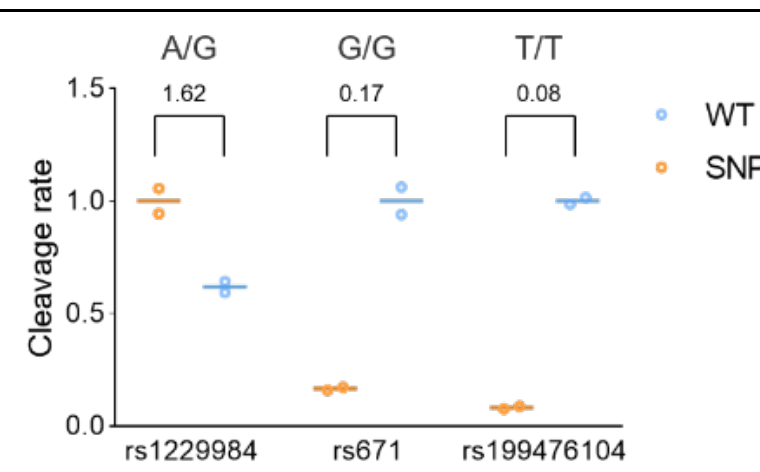
19	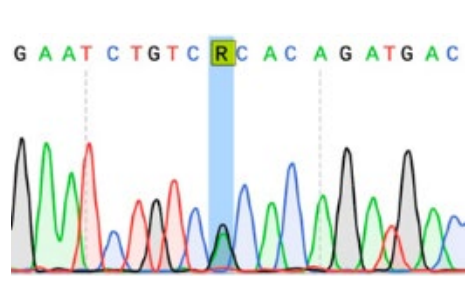	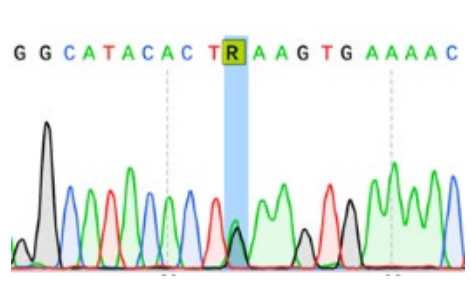	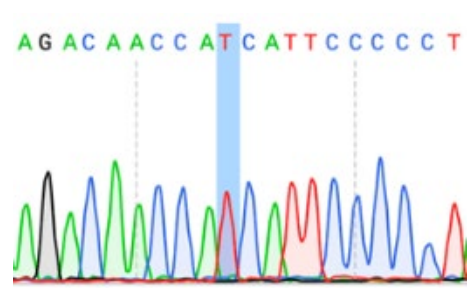	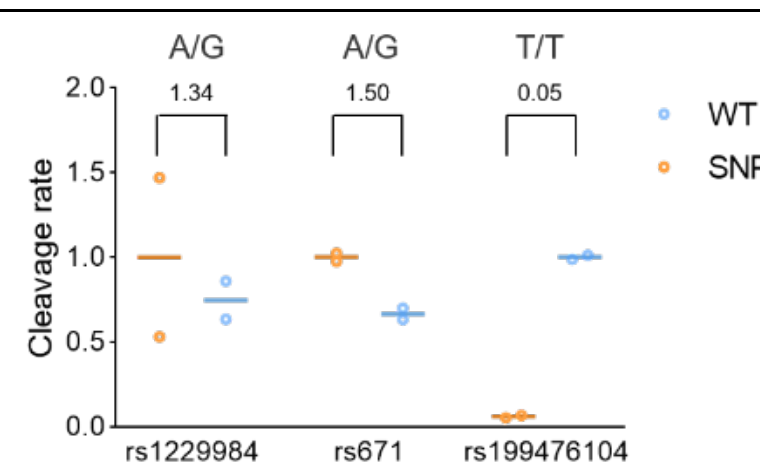

* Genotype calls were assigned based on DF thresholds, with DF values of 0-0.5 classified as wild type, 0.5-2.0 as heterozygous, and >2 as SNP.

* R (highlighted in yellow) denotes a degenerate purine base representing either A (adenine) or G (guanine)

* Source data are provided as a Source Data file.
