# Supplementary material for: Thermodynamically programmed one-pot CRISPR platform for point-of-care SNP genotyping
Source: Nat Commun. 2026 Jul 7;17:8376. doi: 10.1038/s41467-026-75358-1 (PMC13473493; doi:10.1038/s41467-026-75358-1)
Supplement: Supplementary file 4 [file 41467_2026_75358_MOESM6_ESM.pdf]

nature portfolio

Corresponding author(s): I-ming Hsing

Last updated by author(s): Jun 18, 2026

Reporting Summary

Nature Portfolio wishes to improve the reproducibility of the work that we publish. This form provides structure for consistency and transparency in reporting. For further information on Nature Portfolio policies, see our Editorial Policies and the Editorial Policy Checklist.

Statistics

For all statistical analyses, confirm that the following items are present in the figure legend, table legend, main text, or Methods section.

n/a	Confirmed	
[ ]	[x]	The exact sample size (*n*) for each experimental group/condition, given as a discrete number and unit of measurement
[ ]	[x]	A statement on whether measurements were taken from distinct samples or whether the same sample was measured repeatedly
[ ]	[x]	The statistical test(s) used AND whether they are one- or two-sided<br>*Only common tests should be described solely by name; describe more complex techniques in the Methods section.*
[x]	[ ]	A description of all covariates tested
[x]	[ ]	A description of any assumptions or corrections, such as tests of normality and adjustment for multiple comparisons
[ ]	[x]	A full description of the statistical parameters including central tendency (e.g. means) or other basic estimates (e.g. regression coefficient) AND variation (e.g. standard deviation) or associated estimates of uncertainty (e.g. confidence intervals)
[ ]	[x]	For null hypothesis testing, the test statistic (e.g. *F*, *t*, *r*) with confidence intervals, effect sizes, degrees of freedom and *P* value noted<br>*Give P values as exact values whenever suitable.*
[x]	[ ]	For Bayesian analysis, information on the choice of priors and Markov chain Monte Carlo settings
[x]	[ ]	For hierarchical and complex designs, identification of the appropriate level for tests and full reporting of outcomes
[x]	[ ]	Estimates of effect sizes (e.g. Cohen's *d*, Pearson's *r*), indicating how they were calculated

Our web collection on statistics for biologists contains articles on many of the points above.

Software and code

Policy information about availability of computer code

Data collection	The fluorescence signal intensities in this study was determined and collected by using Bio-Rad Real-Time PCR machine. The gels were visualized by Bio-Rad gel imaging system.
Data analysis	Fluorescence signal and qPCR data were analyzed using Bio-Rad CFX Maestro. Microfluidic chip design was performed using SolidWorks 2020. Data analysis was performed with GraphPad Prism 10, Origin Lab version 2018 or Microsoft Excel 2019. Schematic illustrations were created with BioRender, Adobe Illustrator 2024 and Microsoft PowerPoint 2019. Nucleic acid secondary structures were predicted using NUPACK 4.1. Custom SNP chip smartphone application and the quantitative reaction-network model for TEMPO are available at https://github.com/zibin-zhao/TEMPO.

For manuscripts utilizing custom algorithms or software that are central to the research but not yet described in published literature, software must be made available to editors and reviewers. We strongly encourage code deposition in a community repository (e.g. GitHub). See the Nature Portfolio guidelines for submitting code & software for further information.

Data

Policy information about availability of data

All manuscripts must include a data availability statement. This statement should provide the following information, where applicable:

- Accession codes, unique identifiers, or web links for publicly available datasets
- A description of any restrictions on data availability
- For clinical datasets or third party data, please ensure that the statement adheres to our policy

Source data are provided as a Source Data file with this paper. TEMPO-based detection and Sanger sequence result for human samples are provided in Supplementary Data 1. All nucleic acid sequences used in this study are provided in Supplementary Data 2. The Sanger sequencing raw data generated in this study is provided in Supplementary Data 3. Newly generated materials and assay-related information are available for academic research use, and requests should be directed to the corresponding author.

Research involving human participants, their data, or biological material

Policy information about studies with human participants or human data. See also policy information about sex, gender (identity/presentation), and sexual orientation and race, ethnicity and racism.

Reporting on sex and gender	Influenza A and SARS-CoV-2 swab samples used in this study were obtained from the Prince of Wales Hospital under approval from the Clinical Research Ethics Committee of The Chinese University of Hong Kong (CREC-2021-0188). Human genome swab samples used in this study were obtained from volunteers at The Hong Kong University of Science and Technology (HREP-2026-0148). Samples were selected based on availability. Age and sex were not used as variables in sample selection or analysis, and individual-level demographic information was not available to the investigators.
Reporting on race, ethnicity, or other socially relevant groupings	Race, ethnicity and other socially relevant groupings were not collected or used in this study. The clinical swab samples were obtained from the Prince of Wales Hospital. Human genome samples were obtained from recruited volunteers at The Hong Kong University of Science and Technology, and no categorization based on these variables was performed.
Population characteristics	Samples were selected based on availability. Age and sex were not used as variables in sample selection or analysis, and individual-level demographic information was not available to the investigators. Influenza A and SARS-CoV-2 swab samples used in this study were obtained from the Prince of Wales Hospital under approval from the Clinical Research Ethics Committee of The Chinese University of Hong Kong (CREC-2021-0188). Based on qPCR results, 4 samples were positive for Influenza A and 6 samples were negative; 9 samples were positive for SARS-CoV-2 and 13 samples were negative. Human genomic DNA samples were obtained from volunteers recruited at The Hong Kong University of Science and Technology under a protocol approved by the university's Human and Artefacts Research Ethics Committee (HREP-2026-0148). Genotypes of all 20 clinical samples were fully concordant with Sanger sequencing-validated results across three loci: rsl229984 (4 WT, 6 SNP, 10 HET), rs671 (16 WT, 4 HET), and rsl99476104 (20 WT).
Recruitment	Influenza A and SARS-CoV-2 clinical samples were provided by The Prince of Wales Hospital with approval from the Chinese University of Hong Kong Clinical Research Ethics Committee (CREC-2021-0188). Human genomic DNA samples were obtained from volunteers recruited at The Hong Kong University of Science and Technology under a protocol approved by the university's Human and Artefacts Research Ethics Committee (HREP-2026-0148). Participation was voluntary and may therefore be subject to self-selection bias. However, the study focused on analytical performance and SNP genotyping rather than population-level characteristics. Consequently, any potential self-selection bias is unlikely to affect the conclusions of the study.
Ethics oversight	Influenza A and SARS-CoV-2 clinical samples were provided by The Prince of Wales Hospital with approval from the Chinese University of Hong Kong Clinical Research Ethics Committee (CREC-2021-0188). Human genomic DNA samples were obtained from volunteers recruited at The Hong Kong University of Science and Technology under a protocol approved by the university's Human and Artefacts Research Ethics Committee (HREP-2026-0148).

Note that full information on the approval of the study protocol must also be provided in the manuscript.

Field-specific reporting

Please select the one below that is the best fit for your research. If you are not sure, read the appropriate sections before making your selection.

- [x] Life sciences
- [ ] Behavioural & social sciences
- [ ] Ecological, evolutionary & environmental sciences

For a reference copy of the document with all sections, see nature.com/documents/nr-reporting-summary-flat.pdf

Life sciences study design

All studies must disclose on these points even when the disclosure is negative.

Sample size	No statistical method was used to predetermine sample size. Sample sizes were chosen based on standard practice, sample availability and feasibility. Exact n values and whether replicates were technical or biological are provided in the figure legends.
Data exclusions	No data were excluded from the analyses.

Replication	Each experiment was done with at least 2 replicates to ensure that the results are reproducible. All attempts at replication were successful, and standard deviations were within the expected ranges.
Randomization	Samples were allocated to experimental groups according to the predefined assay conditions, such as target identity, genotype, concentration or control status. For synthetic and simulated samples, the order of sample preparation and detection was randomized where applicable to reduce positional or run-order bias. Covariate control was not relevant because the study primarily used synthetic nucleic acid targets and instrument-based quantitative readouts.
Blinding	Blinding was not performed during data collection or analysis because the primary readouts were instrument-based quantitative measurements, including fluorescence and RT-qPCR, and the data were analysed using predefined criteria. Therefore, blinding was not relevant to outcome assessment.

Reporting for specific materials, systems and methods

We require information from authors about some types of materials, experimental systems and methods used in many studies. Here, indicate whether each material, system or method listed is relevant to your study. If you are not sure if a list item applies to your research, read the appropriate section before selecting a response.

Materials & experimental systems

n/a	Involved in the study
☒	☐ Antibodies
☒	☐ Eukaryotic cell lines
☒	☐ Palaeontology and archaeology
☒	☐ Animals and other organisms
☒	☐ Clinical data
☒	☐ Dual use research of concern
☒	☐ Plants

Methods

n/a	Involved in the study
☒	☐ ChIP-seq
☒	☐ Flow cytometry
☒	☐ MRI-based neuroimaging

Plants

Seed stocks	*Report on the source of all seed stocks or other plant material used. If applicable, state the seed stock centre and catalogue number. If plant specimens were collected from the field, describe the collection location, date and sampling procedures.*
Novel plant genotypes	*Describe the methods by which all novel plant genotypes were produced. This includes those generated by transgenic approaches, gene editing, chemical/radiation-based mutagenesis and hybridization. For transgenic lines, describe the transformation method, the number of independent lines analyzed and the generation upon which experiments were performed. For gene-edited lines, describe the editor used, the endogenous sequence targeted for editing, the targeting guide RNA sequence (if applicable) and how the editor was applied.*
Authentication	*Describe any authentication procedures for each seed stock used or novel genotype generated. Describe any experiments used to assess the effect of a mutation and, where applicable, how potential secondary effects (e.g. second site T-DNA insertions, mosiacism, off-target gene editing) were examined.*
