# Supplementary material for: Thermodynamically programmed one-pot CRISPR platform for point-of-care SNP genotyping
Source: Nat Commun. 2026 Jul 7;17:8376. doi: 10.1038/s41467-026-75358-1 (PMC13473493; doi:10.1038/s41467-026-75358-1)
Supplement: Supplementary file 5 [file 41467_2026_75358_MOESM7_ESM.pdf]

nature portfolio

Peer Review File

Thermodynamically programmed one-pot CRISPR platform for point-of-care SNP genotyping

Corresponding Author: Professor I-Ming Hsing

This file contains all reviewer reports in order by version, followed by all author rebuttals in order by version.

Version 0:

Reviewer comments:

Reviewer #1

(Remarks to the Author)
The manuscript by Wu et al. introduces a novel molecular design strategy termed "TEMPO" to address the long-standing challenge of kinetic incompatibility in one-pot CRISPR diagnostics. By embedding a defined free-energy differential into competing primers, the authors achieve autonomous, threshold-gated activation of CRISPR-Cas12a without the need for physical compartmentalization. The theoretical foundation is robustly supported by an ordinary differential equation (ODE) model, and clinical utility is convincingly demonstrated through attomolar-level sensitivity and high-resolution SNP genotyping for HIV drug resistance and HBV targets. Overall, this is a highly innovative and rigorous study that redefines the reaction logic of one-pot molecular diagnostics from a biophysical chemistry perspective and holds substantial translational promise.

I recommend the manuscript for publication after revision, provided the authors can address the following specific scientific questions regarding quantitative boundaries, system robustness, and underlying mechanistic limitations.

Major Comments

The TEMPO system relies on a non-linear, switch-like activation mechanism to gate CRISPR cleavage. While this markedly improves the limit of detection for trace targets, it remains unclear whether this mechanism inherently compresses the linear dynamic range of the assay. If the platform is intended for applications requiring precise initial target quantification (e.g., viral load monitoring), the authors should clarify the mathematical relationship between time-to-peak and initial target concentration and discuss whether a reliable standard curve can be established to support its potential for absolute quantification.

This kinetic stratification depends critically on the precise design of primer hybridization free-energy differentials. Given that nucleic acid binding thermodynamics are highly sensitive to environmental ion concentrations, the authors should evaluate whether complex matrix effects in unpurified clinical samples—such as fluctuations in salt concentration or interference from background nucleic acids—could perturb the preset thermodynamic equilibrium, potentially leading to premature or delayed binding of the protected primers.

Although the initial thermodynamic parameters were designed based on static buffer conditions, the RPA amplification process continuously consumes dNTPs and generates pyrophosphate, resulting in significant depletion of free magnesium ions over time. The authors should discuss whether such dynamic changes in the reaction microenvironment could induce "thermodynamic drift," thereby compromising the stability of the preset free-energy thresholds and affecting batch-to-batch reproducibility.

While the system achieves excellent macroscopic kinetic stratification, the intrinsic stochasticity of molecular collisions implies a finite probability of early microscopic binding events for protected primers prior to reaching the amplification threshold. The authors should explore whether this stochastic "kinetic leakage" could be non-linearly amplified by the high catalytic turnover rate of Cas12a, potentially contributing to baseline noise or an increased risk of false-positive signals.

Once the reaction reaches the threshold and activates Cas12a, its robust trans-cleavage ssDNase activity indiscriminately cleaves single-stranded nucleic acids within the system. The authors should clarify whether this activity degrades free

primers or RPA single-stranded intermediates, thereby acting as a self-limiting negative feedback loop that physically terminates RPA amplification. It would also be valuable to know whether this enzymatic interplay has been accurately captured in their ODE model.

The current thermodynamic design performs well for single-target SNP genotyping. However, modern point-of-care testing increasingly requires multiplexed analysis of multiple targets. Given the biochemical complexity of simultaneously balancing multiple thermodynamic thresholds in a single reaction—without inducing cross-talk or heterodimer formation—the authors should objectively discuss the feasibility of extending the TEMPO platform to multiplexed diagnostics and identify the core mechanistic barriers to such an extension.

Minor Comments

The kinetic model presented in the supplementary materials is detailed and technically sound. To enhance accessibility for a broader audience with biological backgrounds, it is recommended to include a qualitative description that explains the physical meaning of key kinetic parameters in plain language within the context of the TEMPO reaction.

In presenting the SNP genotyping results—particularly for low-copy-number samples or heterozygous states—it is advisable to include statistical significance analyses to quantitatively support the visual differences observed in the kinetic plots.

The fonts used for axis labels and legends in some real-time kinetic charts are relatively small and may be difficult to read when printed or scaled. Enlarging these elements would improve figure clarity and overall readability.

Reviewer #2

(Remarks to the Author)
This manuscript introduces a novel TEMPO platform rooted in thermodynamic encoding, effectively addressing the core contradiction between nucleic acid amplification and CRISPR activation in one-pot diagnostic systems. The study exhibits rigorous scientific methodology, featuring innovative design principles, comprehensive experimental validation, and clear translational potential for point-of-care SNP genotyping and pathogen detection. Notably, the conceptual breakthrough of programming reaction order via primer free-energy asymmetry stands out, while integration with microfluidic technology and smartphone-based readout further enhances its practical applicability.
However, to elevate the manuscript's rigor, clarity, and reproducibility, several minor issues and gaps—pertaining to unresolved details and technical oversights—require attention, as detailed below:
1. Figure citation errors: On line 10 of page 4, "Fig. 2B" should be corrected to "Fig. 1B". Similar inconsistencies appear on pages 9 and 10, where "Fig. 4D-I" incorrectly references content that belongs to "Fig. 3D-I" and should be revised accordingly.
2. Limited sample size for HIV diagnosis: Only 11 clinical samples were tested for HIV detection. To strengthen the reliability and generalizability of the findings, expanding the sample size (e.g., to over 50 specimens) is highly recommended.
3. Lack of storage stability data for lyophilized reagents: While Fig. S14 identifies 5% sucrose + 2% mannitol as the optimal lyoprotectant combination, the manuscript provides no data on the storage stability of lyophilized TEMPO reagents—such as activity retention after 1, 2, or 4 weeks of storage at room temperature or 4°C. Supplementing short-term stability data will further validate the platform's feasibility for practical point-of-care deployment.
4. For improved experimental reproducibility, the full composition of the rapid lysis buffer used for buccal swab samples should be explicitly detailed in the Materials and Methods section.
5. The potential of TEMPO for multiplexed diagnosis remains untested and undiscussed. Given that TEMPO requires four RPA primers per target, concerns arise regarding its compatibility with multi-target RPA amplification and detection. This critical point warrants either experimental validation or a detailed discussion of technical feasibility and potential optimization strategies.
6. The Discussion section outlines two key limitations—quantitative precision inferior to RT-qPCR and SNP discrimination dependence on sequence context—but only briefly mentions data-driven modeling as a potential solution. It would be valuable to expand this section with additional actionable mitigation strategies (e.g., integrating digital isothermal amplification to enhance quantification accuracy).

Reviewer #3

(Remarks to the Author)
In this paper, researchers introduce a one-step CRISRP detection method based on thermodynamic principles, which can be applied for SNP detection.
The article designed two forward primers (FP, FPP) and one reverse primer. The first forward primer (FP) has a higher affinity for the target than the second primer (FPP), and the FP position is upstream of the FPP. Therefore, during RPA amplification, the region of the FP will be amplified first, and after the product accumulation, the region of the FPP will be amplified.
In addition, the researchers made base changes in the design of the FPP, artificially inserting the PAM sequence, so that the FP amplification will not be recognized and cleaved by CAS during the entire amplification process, while the FPP-modified amplification product will be cleaved by CAS. Thus, a one-step method is achieved. They explored the recognition efficiency of different PAM sequences. And they also explored the recognition efficiency of different mutation positions in SNPs. Therefore, they achieved secondary amplification by artificially changing the PAM sequence, and only the second

amplification can be recognized and cleaved by Cas.
This approach is innovative and solves the limitation of the PAM sequence.

The following questions should be addressed to improve the quality of this manuscript:
1. On page 9, in “DNA engineering enables broad-spectrum and highly specific SNP detection section”, the fig 4, D-I should be changed to fig 3, D-I 7.
2. In SI sequence, why these two sequences are completely complementary? These two should be a pair of primers for amplification.

3. According to the design concept of the SNP in the text, the mutation site of MUT-1 should be located at the fourth base of the PAM sequence. This mutation should not affect the recognition of the crRNA. Please explain the H graph in Fig. 2. MUT-No and MUT-1 overlap, while the differences between MUT-No and MUT-1 in the I graph are significant.
4. On page 9, in line 36, the comma between "superior" and "control" should be removed.

Version 1:

Reviewer comments:

Reviewer #2

(Remarks to the Author)
The revised manuscript is greatly revised, and all my concerns have been well resolved. Despite insufficient storage stability of the lyophilized powder, which needs to achieve at least 4-week stability at room temperature for further clinical application, I approve the manuscript for publication owing to its core innovative TEMPO strategy. Future studies are recommended to optimize protectants and lyophilization protocols. Additionally, please correct the spelling error of RPA in the abstract.

Reviewer #3

(Remarks to the Author)
I am happy with all revisions; this version should be published.

Open Access This Peer Review File is licensed under a Creative Commons Attribution 4.0 International License, which permits use, sharing, adaptation, distribution and reproduction in any medium or format, as long as you give appropriate credit to the original author(s) and the source, provide a link to the Creative Commons license, and indicate if changes were made.
In cases where reviewers are anonymous, credit should be given to 'Anonymous Referee' and the source.
The images or other third party material in this Peer Review File are included in the article’s Creative Commons license, unless indicated otherwise in a credit line to the material. If material is not included in the article’s Creative Commons license and your intended use is not permitted by statutory regulation or exceeds the permitted use, you will need to obtain permission directly from the copyright holder.

To view a copy of this license, visit https://creativecommons.org/licenses/by/4.0/

Our responses highlighted in blue to the three reviewers' comments to our Nature Communications manuscript (NCOMMS-26-011593)

Pages 1-8: Reviewer#1, **Pages 9-12**: Reviewer#2, **Pages 13-14**: Reviewer#3.

Reviewer #1

The manuscript by Wu et al. introduces a novel molecular design strategy termed "TEMPO" to address the long-standing challenge of kinetic incompatibility in one-pot CRISPR diagnostics. By embedding a defined free-energy differential into competing primers, the authors achieve autonomous, threshold-gated activation of CRISPR-Cas12a without the need for physical compartmentalization. The theoretical foundation is robustly supported by an ordinary differential equation (ODE) model, and clinical utility is convincingly demonstrated through attomolar-level sensitivity and high-resolution SNP genotyping for HIV drug resistance and HBV targets. Overall, this is a highly innovative and rigorous study that redefines the reaction logic of one-pot molecular diagnostics from a biophysical chemistry perspective and holds substantial translational promise.

Thank you for your comments and suggestions concerning our manuscript. The comments and suggestions are all valuable and very helpful for revising and improving our paper, as well as providing important guidance for our research. We have studied the comments carefully and made corrections that we hope will meet with approval.

I recommend the manuscript for publication after revision, provided the authors can address the following specific scientific questions regarding quantitative boundaries, system robustness, and underlying mechanistic limitations.

Major Comments

1. The TEMPO system relies on a non-linear, switch-like activation mechanism to gate CRISPR cleavage. While this markedly improves the limit of detection for trace targets, it remains unclear whether this mechanism inherently compresses the linear dynamic range of the assay. If the platform is intended for applications requiring precise initial target quantification (e.g., viral load monitoring), the authors should clarify the mathematical relationship between time-to-peak and initial target concentration and discuss whether a reliable standard curve can be established to support its potential for absolute quantification.

Thank you for this valuable suggestion. We agree that the switch-like activation mechanism may constrain the quantitative dynamic range and therefore requires explicit evaluation for quantification purposes. To address this, we experimentally assessed the quantitative performance of TEMPO using a 10-fold serial dilution series from 0.1 aM to 10,000 aM (**Supplementary Fig. 9**). Using a fluorescence threshold of 5,000 a.u., we extracted the threshold time (Tt) for each concentration and constructed a standard curve. TEMPO showed a measurable concentration-dependent response over 1–10,000 aM, with a linear regression coefficient of $R^2 = 0.968$.

We further clarified this point in the Discussion and added ODE simulations incorporating nonspecific amplification (**line 501-502**). As shown in **Supplementary Fig. 24**, nonspecific amplification increasingly affects Tt at low target concentrations, leading to larger deviations from the ideal concentration-dependent spacing and thus reducing quantitative linearity. This behavior is consistent with the experimental data.

Accordingly, TEMPO in its current form is better suited for semi-quantitative analysis within a defined concentration range, rather than high-precision absolute quantification such as viral load monitoring.

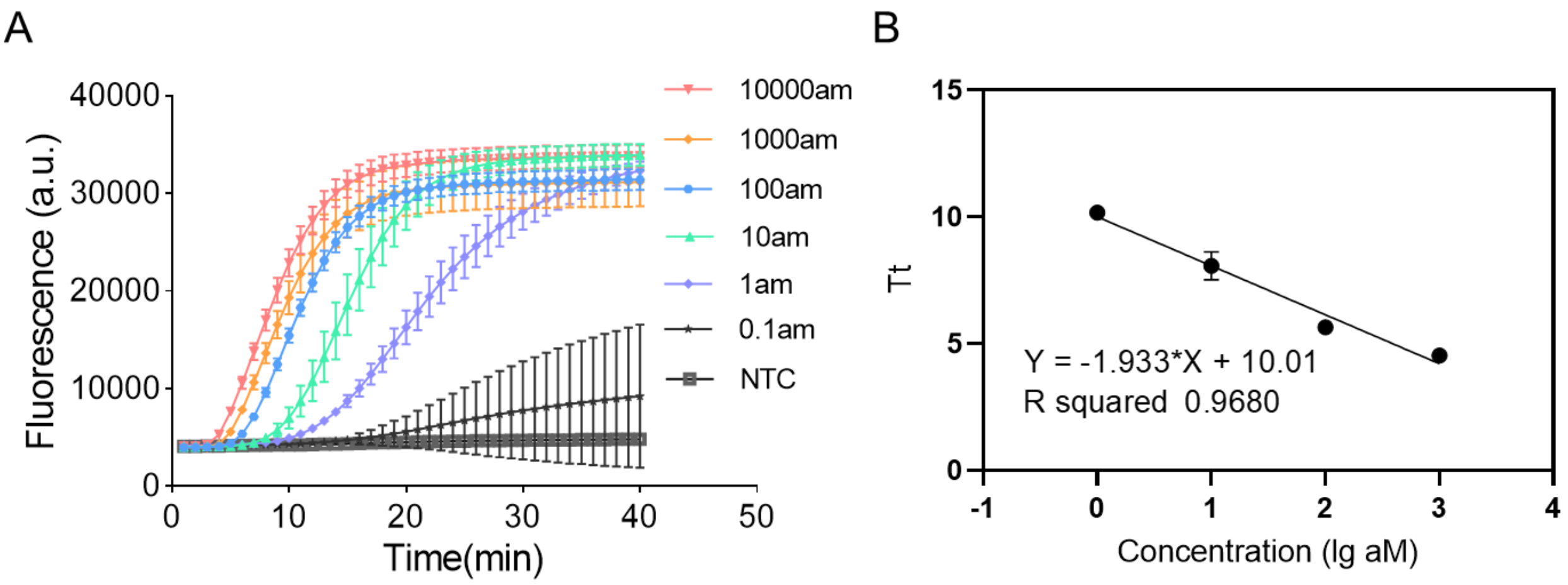

Fig. S9. Analytical sensitivity of the TEMPO assay for detection of HIV derived DNA concentrations. (A) Real-time fluorescence amplification curves obtained using serially diluted HIV derived DNA inputs (10,000 aM, 1,000 aM, 100 aM, 10 aM, 1 aM, and 0.1 aM). Higher target concentrations produced earlier fluorescence onset and faster signal accumulation, whereas lower concentrations exhibited delayed amplification kinetics. (B) Standard curve of the detection platform. Threshold times (Tt) were determined using a fluorescence threshold of 5,000 a.u. across the tested concentration range. NTC, non-template control reaction; n = 3 technical replicates; Error bars represent the means ± SD.

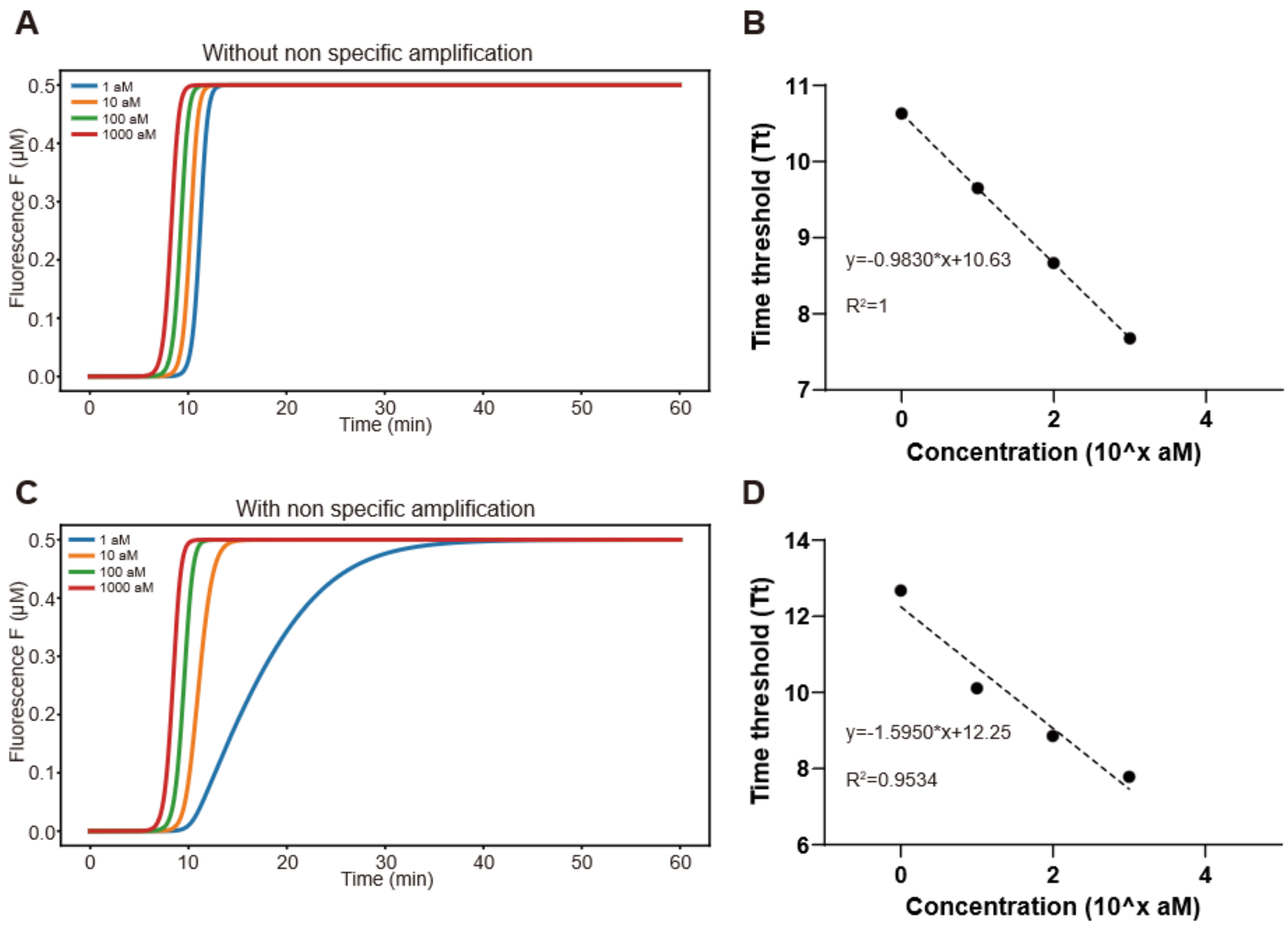

Fig. S24. ODE modelling of how nonspecific amplification influences quantitative performance. (A) Simulated fluorescence generation kinetics for a gradient of template concentrations in the absence of nonspecific amplification ($k_{ns\_init} = 0$). (B) Corresponding standard curve for a, generated using the threshold time (Tt), defined as the time required for the fluorescence signal to reach a preset threshold. In the absence of nonspecific amplification, the simulated signal trajectories showed near-ideal behavior, with approximately equal Tt intervals between successive 10-fold changes in template concentration, consistent with quantitative detection performance ($R^2 = 1$). (C) Simulated fluorescence generation kinetics for a gradient of template concentrations in the presence of nonspecific amplification ($k_{ns\_init} =$

1.5×10^{-5}). (D) Corresponding standard curve for c, generated using Tt values. Because nonspecific amplification is an intrinsic feature that must be considered in isothermal amplification, its inclusion in the model caused the Tt values at lower template concentrations to shift to earlier times relative to the ideal case. This compression of Tt spacing reduced the accuracy of template quantification, indicating impaired quantitative performance ($R^2 = 0.9534$).

2. This kinetic stratification depends critically on the precise design of primer hybridization free-energy differentials. Given that nucleic acid binding thermodynamics are highly sensitive to environmental ion concentrations, the authors should evaluate whether complex matrix effects in unpurified clinical samples—such as fluctuations in salt concentration or interference from background nucleic acids—could perturb the preset thermodynamic equilibrium, potentially leading to premature or delayed binding of the protected primers.

Thank you for this insightful suggestion regarding the thermodynamic stability of our system. We agree that potential matrix interference should be directly evaluated. To address this point, we compared TEMPO reaction kinetics in purified samples and unpurified samples (crude extracts). The fluorescence kinetics in crude extracts were nearly identical to those of the purified samples, indicating that the clinical matrix did not measurably disrupt the preset thermodynamic hierarchy of the system (**Supplementary Fig. 13**).

These results show that TEMPO remains robust in the presence of crude sample components, including background genomic DNA and endogenous ionic fluctuations. We have revised the text accordingly to clarify that the current buffer and primer design are sufficient to preserve the intended kinetic stratification under direct-sample conditions (**line 366-368**).

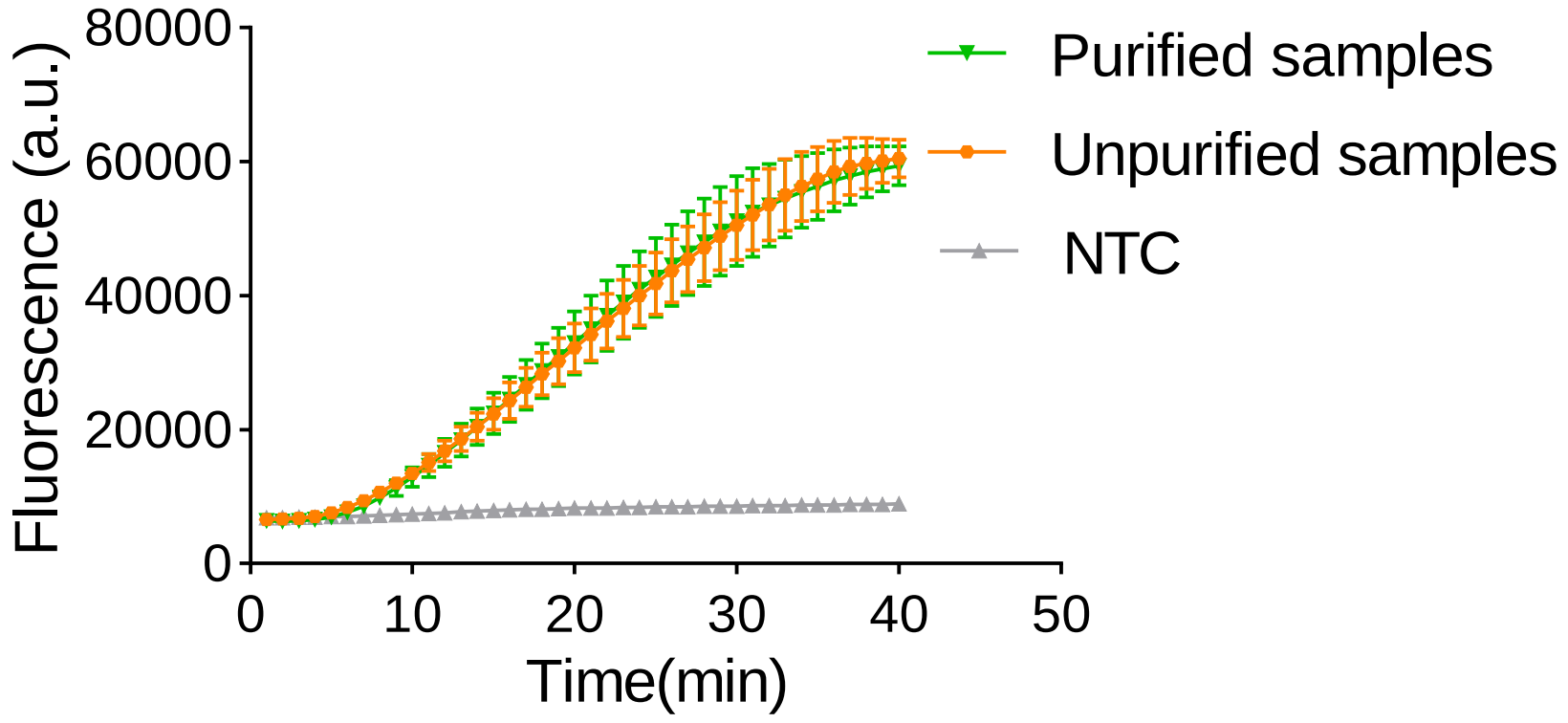

Fig. S13. Robustness of the diagnostic system against complex matrix effects. Real-time fluorescence kinetic curves were compared between the positive control (PC, with purified templates) and the unpurified samples (crude human clinical extracts) to evaluate the impact of matrix interference. NTC, non-template control reaction; n = 3 technical replicates; Error bars represent the means ± SD.

3. Although the initial thermodynamic parameters were designed based on static buffer conditions, the RPA amplification process continuously consumes dNTPs and generates pyrophosphate, resulting in significant depletion of free magnesium ions over time. The authors should discuss whether such dynamic changes in the reaction microenvironment could induce "thermodynamic drift," thereby compromising the stability of the preset free-energy thresholds and affecting batch-to-batch reproducibility.

Thank you for this insightful suggestion. We agree that Mg^{2+} fluctuations during RPA could in principle affect the preset thermodynamic thresholds. To address this, we added both a stoichiometric analysis and an experimental Mg^{2+} perturbation test (**Supplementary Fig. 5**).

Our calculation showed that, under our reaction conditions, the maximum Mg^{2+} decrease expected from amplification-associated pyrophosphate formation is limited relative to the initial 20 mM Mg^{2+} concentration (In RPA, the main sink of free Mg^{2+} is expected to be pyrophosphate (PPi) released during nucleotide incorporation. Since each incorporated nucleotide releases 1 mol of PPi, and each mol of PPi can sequester up to 2 mol of Mg^{2+}, the maximal Mg^{2+} decrease in our system can be estimated stoichiometrically. Assuming 400 nM primer consumption and generation of a 200 bp amplicon, the maximal product concentration would be ~400 nM, corresponding to ~0.16 mM incorporated nucleotides and a theoretical Mg^{2+} decrease of ~0.32 mM, which is small relative to the initial 20 mM Mg^{2+} concentration.)[1]. We then tested TEMPO across 15–21 mM magnesium acetate. The fluorescence kinetics were essentially unchanged between 19 and 21 mM, indicating that Mg^{2+} variations within the range expected during RPA do not measurably affect the programmed reaction behavior. A moderate kinetic reduction was only observed at 15 mM Mg^{2+}.

These results indicate that amplification-associated Mg^{2+} changes are insufficient to cause meaningful thermodynamic drift in TEMPO under standard conditions. We have revised the text accordingly to clarify that this effect is unlikely to compromise the stability of the preset free-energy thresholds or batch-to-batch reproducibility (**line 142-145**).

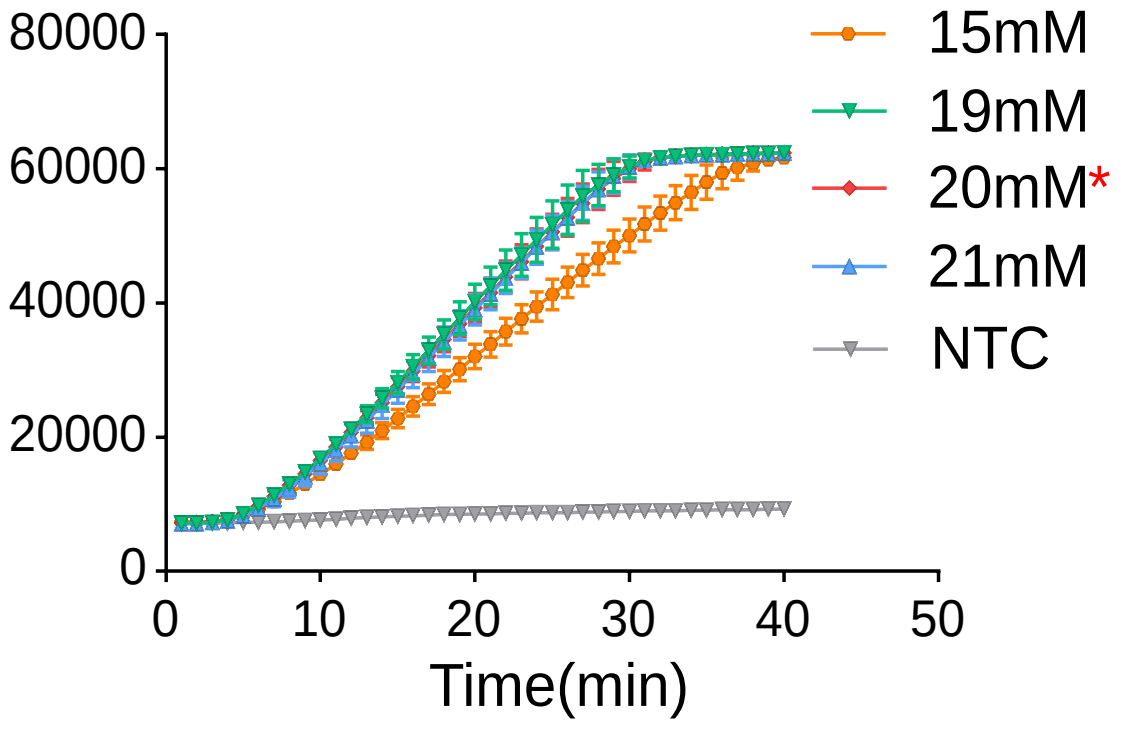

Fig. S5. Robustness of TEMPO kinetics against magnesium ion fluctuations. To evaluate the impact of "thermodynamic drift" caused by potential Mg^{2+} depletion during RPA amplification, we monitored the fluorescence trajectories at varying initial magnesium acetate concentrations (15, 19, 20, and 21 mM). The overlapping kinetic curves across this concentration gradient demonstrate that the preset thermodynamic equilibrium is highly resistant to fluctuations in the ionic environment. 20 mM* denotes the standard concentration used in this study. NTC, non-template control. Data are presented as mean ± SD (n = 3). In RPA, the main sink of free Mg^{2+} is expected to be pyrophosphate (PPi) released during nucleotide incorporation. Since each incorporated nucleotide releases 1 mol of PPi, and each mol of PPi can sequester up to 2 mol of Mg^{2+}, the maximal Mg^{2+} decrease in our system can be estimated stoichiometrically. Assuming 400 nM primer consumption and generation of a 200 bp amplicon, the maximal product concentration would be ~400 nM, corresponding to ~0.16 mM incorporated nucleotides and a theoretical Mg^{2+} decrease of ~0.32 mM, which is small relative to the initial 20 mM Mg^{2+} concentration[1].

4. While the system achieves excellent macroscopic kinetic stratification, the intrinsic stochasticity of molecular collisions implies a finite probability of early microscopic binding events for protected primers prior to reaching the amplification threshold. The authors should explore whether this stochastic "kinetic leakage" could be non-linearly amplified by the high catalytic turnover rate of Cas12a, potentially contributing to baseline noise or an increased risk of false-positive signals.

Thank you for this insightful comment. We agree that stochastic microscopic leakage should be evaluated experimentally. To address this, we analyzed 20 independent low-input positive samples (1 aM) and 20 non-template controls (NTCs) (**Supplementary Fig. 10**). The NTC group showed no appreciable baseline elevation, and the 1 aM samples retained a clear delayed-onset fluorescence profile without premature signal emergence.

Using an endpoint threshold defined as the mean NTC signal plus three standard deviations, the false-positive rate in the NTC group was 5% (1/20), whereas the detection rate for 1 aM samples was 100% (20/20). These results indicate that any stochastic leakage, if present, remains rare and does not materially compromise assay discrimination under the tested conditions.

Because NTC samples lack template, the single false-positive event is unlikely to arise from the protected-primer leakage mechanism itself, and more likely reflects low-level nonspecific background noise.

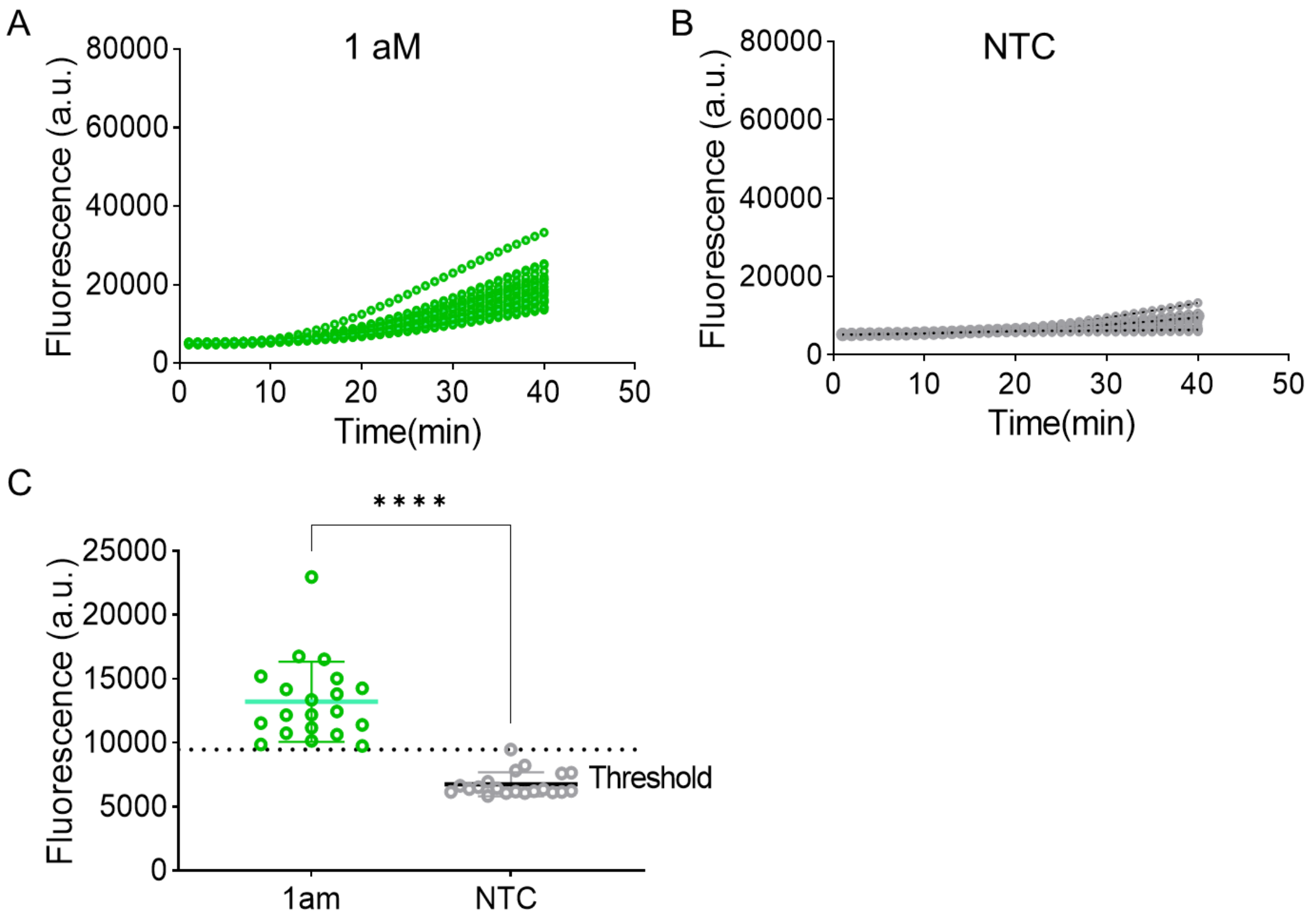

Fig. S10. Fluorescence signal distribution at the limit of detection. (A and B) Real-time fluorescence kinetic curves of the assay in the presence of 1 aM HIV-derived DNA and non-template control (NTC). Each curve represents an individual technical replicate (n = 20). (C) Statistical analysis of end-point fluorescence intensity at 30 min for the 1 aM target and NTC. Each point represents an independent replicate (n = 20 per group). The dashed line indicates the detection threshold defined as the mean fluorescence of NTC plus three standard deviations (mean + 3σ). Using this criterion, all 1 aM samples (20/20) were classified as positive, while only a small fraction of NTC replicates (1/20) exceeded the threshold, corresponding to a low false-positive rate. Statistical significance was evaluated using a two-tailed t-test (****, $P < 0.0001$).

5. Once the reaction reaches the threshold and activates Cas12a, its robust trans-cleavage ssDNase activity indiscriminately cleaves single-stranded nucleic acids within the system. The authors should clarify whether this activity degrades free primers or RPA single-stranded intermediates, thereby acting as a self-limiting negative feedback loop that physically terminates RPA amplification. It would also be valuable to know whether this enzymatic interplay has been accurately captured in their ODE model.

Thank you for this insightful comment. We agree that activated Cas12a could in principle cleave free primers or transient single-stranded amplification intermediates and thereby introduce negative feedback on RPA. To address this, we combined modelling and experiment. Our data showed that activated Cas12a had only weak trans-cleavage activity toward primers (**Supplementary Fig. 7A, B**), and RPA amplification was not terminated during the TEMPO reaction

(**Supplementary Fig. 7C**). These results indicate that Cas12a-mediated collateral cleavage does not act as a dominant self-limiting mechanism under our experimental conditions.

This effect was also included in our ODE model by treating primers as a secondary trans-cleavage substrate with lower activity than the reporter. The simulation showed that primer cleavage may slightly reduce final amplicon yield, but does not extinguish amplification or measurably affect fluorescence output in the one-pot assay (**Supplementary Fig. 7D-F**)

We have revised the text accordingly to clarify that this enzymatic interplay is captured in the model, but is not sufficient to physically terminate RPA amplification in TEMPO (**line 216-220**).

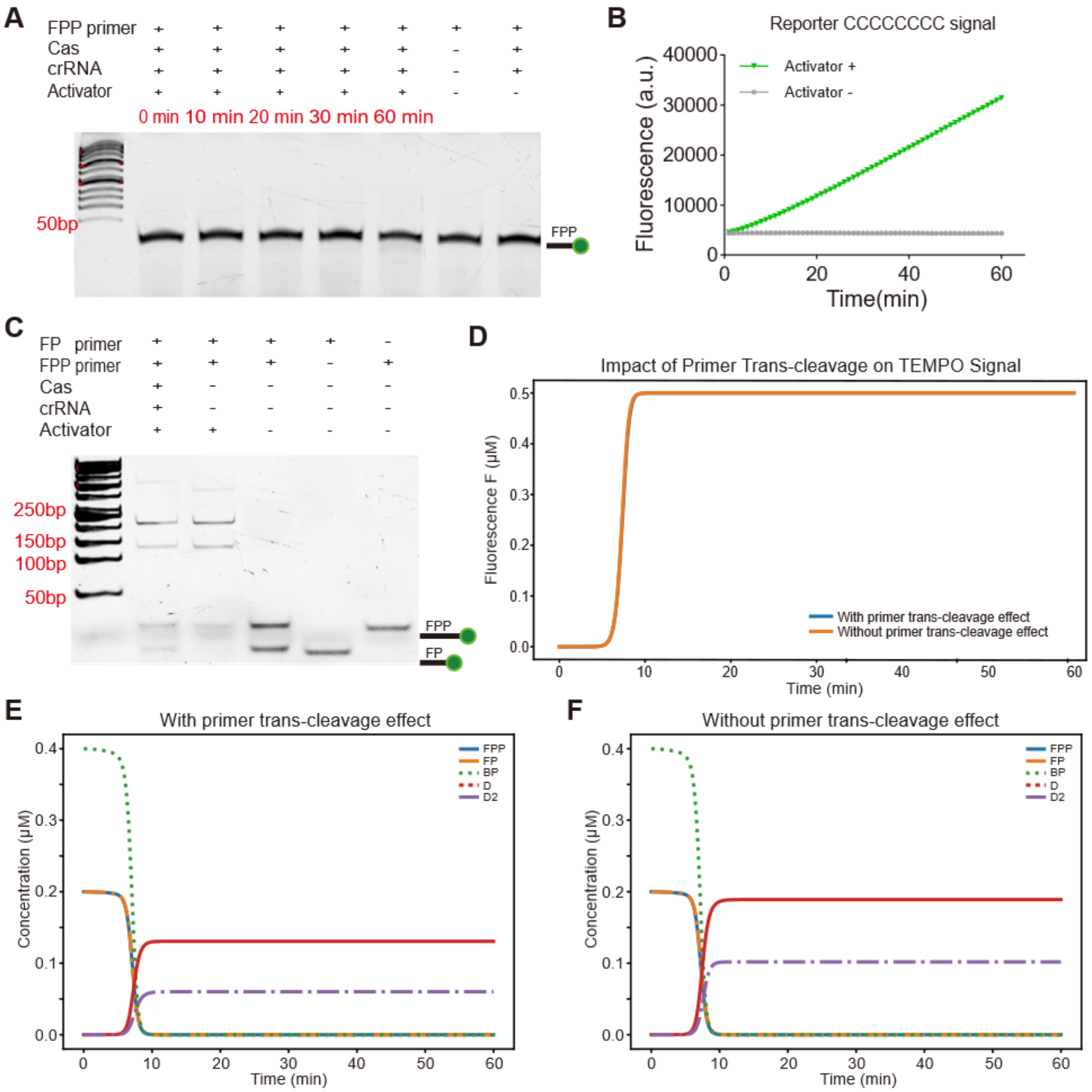

Fig. S7. Impact of primer trans-cleavage on TEMPO signal generation. (A) Time-course analysis of primer stability under Cas12a trans-cleavage activity. Gel electrophoresis illustrates the impact of activated Cas12a on primer integrity across a series of incubation time points (0, 10, 20, 30, and 60 min). (B) Positive control confirming that, under the same experimental conditions as in A, activated Cas12a efficiently cleaved the fluorescent reporter. n = 3 technical replicates; error bars indicate mean ± SD. (C) Gel electrophoresis of RPA products subjected to Cas12a trans-cleavage. Results indicate limited degradation of the target amplicons within a 30-min timeframe post-activation of the Cas12a-crRNA complex. (D) Simulated signal trajectories in the ODE model with primer trans-cleavage. Experimental results showed

that activated CRISPR–Cas12a exhibited only weak trans-cleavage activity toward primers. Consistent with these observations, the ODE model indicated that primer trans-cleavage slightly reduced amplicon yield but produced no observable effect on fluorescence signal generation in the one-pot reaction. (E and F) Simulated variation of molecular species in the ODE model with (E) or without (F) primer trans-cleavage.

6. The current thermodynamic design performs well for single-target SNP genotyping. However, modern point-of-care testing increasingly requires multiplexed analysis of multiple targets. Given the biochemical complexity of simultaneously balancing multiple thermodynamic thresholds in a single reaction—without inducing cross-talk or heterodimer formation, the authors should objectively discuss the feasibility of extending the TEMPO platform to multiplexed diagnostics and identify the core mechanistic barriers to such an extension.

Thank you for highlighting multiplexing as an important direction for point-of-care diagnostics. We agree that true homogeneous multiplexing within a single one-pot reactor remains challenging and should be stated more clearly. We have therefore revised the Discussion (**line 509-521**) to explicitly acknowledge this limitation.

At the same time, the current study already supports a practical form of multiplexing through spatial compartmentalization on chip. In our microfluidic format, six independent reaction chambers enable simultaneous analysis of three SNP loci, with two chambers assigned to each site. Because each chamber functions as an isolated one-pot TEMPO module, this design enables parallel multiplexed detection while avoiding biochemical cross-talk.

We have also clarified in the revised Discussion (**line 509-521**) that extension to true single-reactor multiplexing will require further optimization, including control of primer cross-reactivity, competition for shared amplification resources, indiscriminate Cas12a trans-cleavage, and overlap between target-specific activation windows.

Minor Comments

1. The kinetic model presented in the supplementary materials is detailed and technically sound. To enhance accessibility for a broader audience with biological backgrounds, it is recommended to include a qualitative description that explains the physical meaning of key kinetic parameters in plain language within the context of the TEMPO reaction.

Thank you for this constructive suggestion. We agree that the kinetic model would benefit from a more accessible qualitative explanation for readers with biological backgrounds. In response, we added a new subsection, "**Qualitative interpretation of key kinetic parameters,**" to **Supplementary Note 1**.

In this section, we explain in plain language the physical meaning of the main parameters in the TEMPO reaction, including the asymmetry between FP- and FPP-mediated amplification, the distinction between PAM-independent and PAM-dependent Cas12a recognition, differences in reporter-cleavage efficiency, delayed negative feedback from primer degradation, and the contribution of rehybridization and nonspecific amplification pathways.

We believe this addition improves the accessibility of the model by linking the key parameters directly to the underlying molecular processes in TEMPO.

2. In presenting the SNP genotyping results—particularly for low-copy-number samples or heterozygous states—it is advisable to include statistical significance analyses to quantitatively support the visual differences observed in the kinetic plots.

Thank you for this helpful suggestion. To provide a clearer and more quantitative representation of the SNP genotyping states, we have now adopted a unified discrimination factor (DF)-based metric rather than relying only on visual differences in the kinetic curves. Specifically, the DF was calculated as the ratio of the mean fluorescence signal obtained

from the SNP-targeting reaction to that from the WT-targeting reaction. Based on this ratio, samples were classified as follows: 0–0.5 as wild type (WT), 0.5–2.0 as heterozygous, and >2.0 as SNP. This classification scheme is now presented in **Fig. 5C, D** and **Supplementary Table 2**, where the DF values provide a direct quantitative basis for genotype assignment, including for heterozygous and low-copy-number samples.

3. The fonts used for axis labels and legends in some real-time kinetic charts are relatively small and may be difficult to read when printed or scaled. Enlarging these elements would improve figure clarity and overall readability.

Thank you for this helpful suggestion. We have revised the figures to improve readability by enlarging the font size of the axis labels, tick labels, and legends in the real-time kinetic plots. We agree that these adjustments improve clarity, particularly when the figures are printed or viewed at reduced scale.

Reviewer #2

This manuscript introduces a novel TEMPO platform rooted in thermodynamic encoding, effectively addressing the core contradiction between nucleic acid amplification and CRISPR activation in one-pot diagnostic systems. The study exhibits rigorous scientific methodology, featuring innovative design principles, comprehensive experimental validation, and clear translational potential for point-of-care SNP genotyping and pathogen detection. Notably, the conceptual breakthrough of programming reaction order via primer free-energy asymmetry stands out, while integration with microfluidic technology and smartphone-based readout further enhances its practical applicability.

Thank you for the positive and encouraging assessment of our work. We are grateful for your thoughtful comments and constructive suggestions, which have been very helpful in improving the clarity and rigor of the manuscript. We have carefully considered all points raised and revised the manuscript accordingly. We hope that the revisions have adequately addressed your concerns.

However, to elevate the manuscript's rigor, clarity, and reproducibility, several minor issues and gaps—pertaining to unresolved details and technical oversights—require attention, as detailed below:

1. Figure citation errors: On line 10 of page 4, "Fig. 2B" should be corrected to "Fig. 1B". Similar inconsistencies appear on pages 9 and 10, where "Fig. 4D-I" incorrectly references content that belongs to "Fig. 3D-I" and should be revised accordingly.

Thank you for carefully identifying these figure citation errors. The incorrect citations have been corrected in the revised manuscript, including changing "Fig. 2B" to "Fig. 1B" in **line 117**, and revising "Fig. 4D–I" to "Fig. 3D–I" in **line 309-318**.

2. Limited sample size for HIV diagnosis: Only 11 clinical samples were tested for HIV detection. To strengthen the reliability and generalizability of the findings, expanding the sample size (e.g., to over 50 specimens) is highly recommended.

Thank you for this important comment regarding the sample size and scope of the clinical validation. We agree that the initial HIV dataset was limited and does not by itself support broad clinical generalizability. We wish to clarify that these samples were mock clinical samples and were included as a proof-of-concept validation.

To strengthen the manuscript, we expanded the clinical validation to include Influenza A and SARS-CoV-2 using real clinical specimens. TEMPO showed 100% concordance with RT-qPCR in 10 Influenza A samples and 22 SARS-CoV-2 samples. These results are now included in Fig. 4D, E and Supplementary Table 1. We also included SNP genotyping data from 20 human clinical swab samples in Fig. 5. Taken together, our expanded clinical validation now encompasses 52 samples across three distinct viral pathogens and a separate genotyping cohort, collectively supporting the robustness and generalizability of the TEMPO platform. While we acknowledge that larger cohort studies would further strengthen clinical translation, the diversity of targets and sample types across these validation sets provides meaningful evidence of broad applicability.

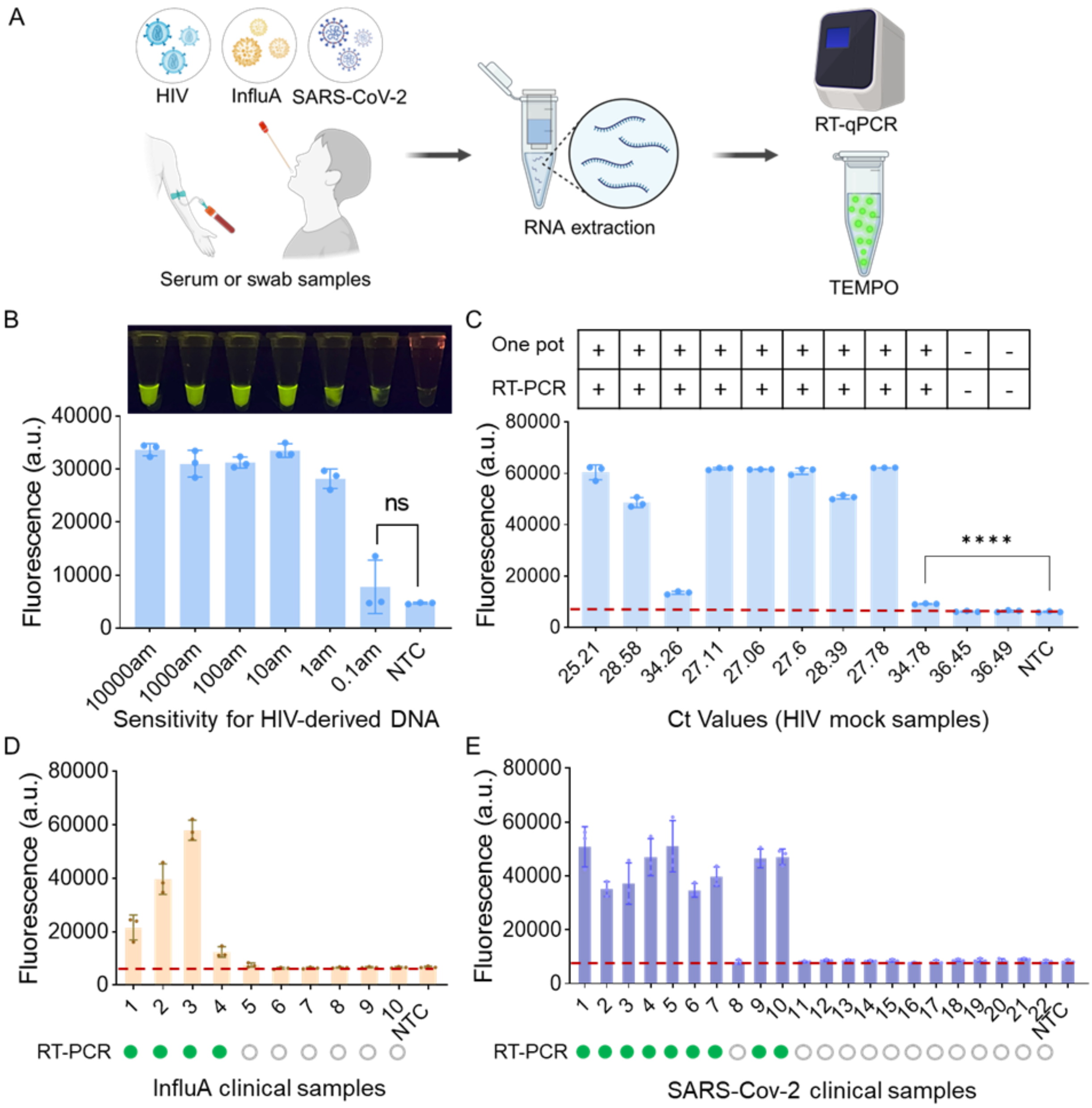

Fig. 4. Rapid pathogen detection via TEMPO. (A) Schematic of the streamlined diagnostic workflow for multi-pathogen detection. Clinical specimens (serum or swabs) undergo rapid RNA extraction followed by parallel evaluation via TEMPO and gold-standard RT-qPCR. (B) Sensitivity of TEMPO for detecting different concentrations of HIV-derived DNA (10000 aM,1000 aM, 100 aM, 10 aM,1 aM, and 0.1 aM). Strong fluorescence signals were observed down to 1 aM concentration, with clear visual fluorescence under blue light. NTC, non-template control reaction; n = 3 technical replicates; Error bars represent the means ± SD. The unpaired two-tailed t-test was used to analyze the statistical significance; ns, not significant. (C) Fluorescence intensity of TEMPO platform across extracted HIV RNA mock samples with varying RT-qPCR Ct values. NTC, non-template control reaction; n = 3 technical replicates; Error bars represent the means ± SD. The unpaired two-tailed t-test was used to analyze the statistical significance. (D and E) Validation of TEMPO across clinical specimens. Endpoint fluorescence output of TEMPO measured at 30 min for (D) 10 Influenza A and (E) 22 SARS-CoV-2 clinical samples, with corresponding RT-qPCR results indicated below (green dots: positive; empty circles: negative). The dashed red line represents the threshold for positive detection. For all panels, NTC, non-template control reaction; n = 3 technical replicates; Error bars represent the means ± SD.

3. Lack of storage stability data for lyophilized reagents: While Fig. S14 identifies 5% sucrose + 2% mannitol as the optimal lyoprotectant combination, the manuscript provides no data on the storage stability of lyophilized TEMPO

reagents—such as activity retention after 1, 2, or 4 weeks of storage at room temperature or 4°C. Supplementing short-term stability data will further validate the platform's feasibility for practical point-of-care deployment.

Thank you for this insightful suggestion regarding storage stability of the lyophilized TEMPO reagents. We agree that storage stability data are important for evaluating the practical feasibility of lyophilized TEMPO reagents. To address this, we performed additional short-term stability experiments using the optimized lyophilization formulation (5% sucrose + 2% mannitol) and assessed reagent performance after storage for 1–4 weeks at room temperature, 4 °C, and −20 °C (**Supplementary Fig. 19**). The lyophilized reagents retained robust activity at 4 °C and −20 °C over 4 weeks, with minimal loss of amplification and CRISPR performance. In contrast, activity gradually declined during room-temperature storage and was nearly lost after 4 weeks. We have revised the text accordingly to clarify the storage conditions that support stability of the lyophilized TEMPO system and its practical relevance for deployment (**line 425-431**).

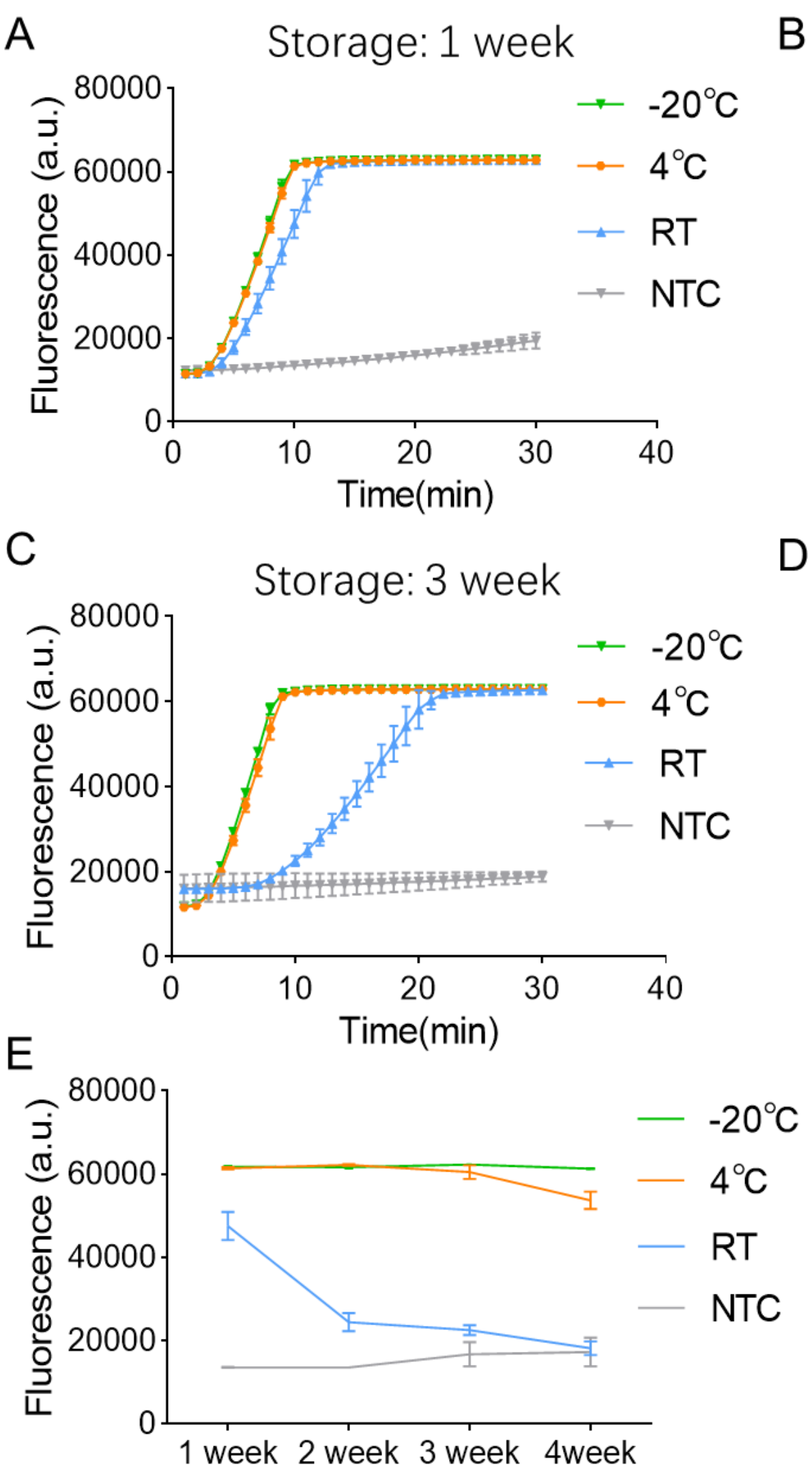

Fig. S19. Storage stability and shelf-life assessment of lyophilized TEMPO reagents. (A-D) Real-time fluorescence kinetics of the lyophilized TEMPO assay reagents stored at various temperatures (-20°C, 4°C, and room temperature) over 4 weeks. Each panel represents a specific storage duration: 1 week, 2 weeks, 3 weeks, and 4 weeks. The performance of the reagents was evaluated by monitoring the fluorescence recovery at 30-minute intervals upon rehydration and initiation of the reaction. (E) Statistical comparison of end-point fluorescence intensities at 10 min for samples across various storage durations (1, 2, 3, and 4 weeks); n = 3 technical replicates; error bars indicate mean ± SD; NTC denotes non-template controls.

4. For improved experimental reproducibility, the full composition of the rapid lysis buffer used for buccal swab samples should be explicitly detailed in the Materials and Methods section.

Thank you for your suggestion. We completely agree that transparency regarding chemical reagents is vital for reproducibility. The rapid lysis buffer used in this study is a commercially available product, the Rapid Nucleic Acid Release Reagent (Amp-future, Changzhou, China). While the exact proprietary formulation is not disclosed by the manufacturer, we have updated the Materials and Methods section to explicitly name the supplier and include the specific product information to ensure that other researchers can accurately replicate our experimental conditions (**line 563; line 603**).

5. The potential of TEMPO for multiplexed diagnosis remains untested and undiscussed. Given that TEMPO requires four RPA primers per target, concerns arise regarding its compatibility with multi-target RPA amplification and detection. This critical point warrants either experimental validation or a detailed discussion of technical feasibility and potential optimization strategies.

Thank you for this insightful comment regarding the scalability of the TEMPO platform for multiplexed diagnostics. We agree that true single-pot multiplexing has not yet been experimentally validated in this study and should be discussed more explicitly. We have therefore revised the Discussion (**line 509-521**) to clarify this limitation and the key technical challenges.

At the same time, the current study already supports a practical form of multiplexing through spatial compartmentalization on chip. In our microfluidic format, six independent reaction chambers enable simultaneous analysis of three SNP loci, with two chambers assigned to each target. Because each chamber functions as an isolated one-pot TEMPO module, this design enables parallel multiplexed detection while avoiding biochemical cross-talk.

We have also added discussion of the main barriers to true homogeneous multiplexing, including primer cross-reactivity, competition for shared amplification resources, indiscriminate Cas12a trans-cleavage, and overlap between target-specific activation windows. These points are now discussed in the revised manuscript (**line 509-521**).

6. The Discussion section outlines two key limitations—quantitative precision inferior to RT-qPCR and SNP discrimination dependence on sequence context—but only briefly mentions data-driven modeling as a potential solution. It would be valuable to expand this section with additional actionable mitigation strategies (e.g., integrating digital isothermal amplification to enhance quantification accuracy).

Thank you for this constructive suggestion. We agree that the Discussion is greatly strengthened by providing concrete and actionable strategies such as the transition from bulk to digital formats.

As suggested, we have expanded this section to include a more detailed outlook on enhancing the quantitative precision and robustness of TEMPO. Specifically, we now highlight the integration of TEMPO into digital isothermal amplification architectures as a primary route toward achieving absolute quantification, which would inherently mitigate the limitations associated with bulk kinetic measurements (**line 531-533**).

Reviewer #3

In this paper, researchers introduce a one-step CRISRP detection method based on thermodynamic principles, which can be applied for SNP detection. The article designed two forward primers (FP, FPP) and one reverse primer. The first forward primer (FP) has a higher affinity for the target than the second primer (FPP), and the FP position is upstream of the FPP. Therefore, during RPA amplification, the region of the FP will be amplified first, and after the product accumulation, the region of the FPP will be amplified. In addition, the researchers made base changes in the design of the FPP, artificially inserting the PAM sequence, so that the FP amplification will not be recognized and cleaved by CAS during the entire amplification process, while the FPP-modified amplification product will be cleaved by CAS. Thus, a one-step method is achieved. They explored the recognition efficiency of different PAM sequences. And they also explored the recognition efficiency of different mutation positions in SNPs. Therefore, they achieved secondary amplification by artificially changing the PAM sequence, and only the second amplification can be recognized and cleaved by Cas. This approach is innovative and solves the limitation of the PAM sequence.

Thank you for your positive and encouraging assessment of our work. We are grateful for your thoughtful evaluation and for recognizing the innovation of the TEMPO strategy, particularly its use of thermodynamic programming to coordinate one-pot amplification and CRISPR activation while overcoming PAM-dependent target limitations. Your supportive comments are greatly appreciated. We have carefully considered your suggestions and revised the manuscript accordingly, and we hope that the revised version meets with your approval.

The following questions should be addressed to improve the quality of this manuscript:

1. On page 9, in "DNA engineering enables broad-spectrum and highly specific SNP detection section", the fig 4, D-I should be changed to fig 3, D-I 7.

Thank you for carefully identifying this error. The figure citation has been corrected from "**Fig. 4D–I**" to "**Fig. 3D–I**" in the revised manuscript.

2. In SI sequence, why these two sequences are completely complementary? These two should be a pair of primers for amplification.

Thank you for pointing this out. The two sequences in question (**Sequence Nos. 20-23**) are not an amplification primer pair, but rather a pair of complementary oligonucleotides used to assemble a short double-stranded template substrate for mechanistic validation. We agree that the original labels, HIV-template-FP and HIV-template-BP, could be misunderstood as forward and backward amplification primers.

To avoid this ambiguity, we have revised the nomenclature in the Supplementary Information as follows:

HIV-template-FP → HIV-template-top

HIV-template-BP → HIV-template-bottom

We have made the same clarification for the corresponding PAM-containing template strands where applicable. This revision is intended to clearly distinguish these complementary template oligonucleotides from the actual amplification primers and prevent further confusion.

3. According to the design concept of the SNP in the text, the mutation site of MUT-1 should be located at the fourth base of the PAM sequence. This mutation should not affect the recognition of the crRNA. Please explain the H graph in Fig. 2. MUT-No and MUT-1 overlap, while the differences between MUT-No and MUT-1 in the I graph are significant.

Thank you for this insightful question. We agree that this point requires clarification.

In TEMPO, the design of the FPP primer is highly flexible. In the initial design, the FPP primer was engineered primarily to alter the PAM sequence and thereby introduce a thermodynamic penalty relative to FP, enabling temporal separation between amplification and CRISPR activation in the one-pot format. Subsequently, we found that the FPP primer could also be extended into the region overlapping with the crRNA spacer, up to the sequence immediately upstream of the SNP site. This design creates an additional degree of control: by mutating the FPP–spacer overlap region, one can not only tune primer hybridization and amplification kinetics, but also indirectly program the position and identity of the mismatch seen by the crRNA. This provides an additional kinetic dimension for improving single-nucleotide discrimination.

Accordingly, Fig. 2G–I was intended to investigate how mutations introduced within the FPP–spacer overlap region affect reaction kinetics, rather than to test only PAM perturbation. Although the mutations in MUT-1 in Fig. 2H and Fig. 2I both correspond to the first position within the crRNA spacer, the sequence contexts are different because the two panels use different DNA targets. In particular, the nucleotide substitution differs between the two cases: in Fig. 2H, the mutation changes A to C, whereas in Fig. 2I, it changes C to A. These two substitutions are expected to produce different thermodynamic consequences for crRNA–target mismatch formation, which may explain why MUT-1 shows little change relative to MUT-No in Fig. 2H but a much stronger effect in Fig. 2I[2].

In addition, the same mutation is positioned differently with respect to the FPP primer itself in the two targets. In the design used for Fig. 2I, the mutated position lies closer to the 3′ end of the FPP primer than in Fig. 2H. Because mismatches near the 3′ end generally have a stronger impact on primer extension efficiency in RPA, this is likely to make the kinetics in Fig. 2I more sensitive to sequence perturbation in this region.

We have now clarified this design rationale and the interpretation of Fig. 2H and Fig. 2I in the revised manuscript (**line 230-236).**

FPP2-HIV-0 (Fig. 2H)	TACAGTGCAGGGGAAAGAATAATTGATATAATAG
FPP2-HIV-1 (Fig. 2H)	TACAGTGCAGGGGAAAGAATAATTGCTATAATAG
FPP4- ND6-0 (Fig. 2I)	AGCCATCGCTGTAGTATATCCAAAGATTACCA
FPP4- ND6-1 (Fig. 2I)	AGCCATCGCTGTAGTATATCCAAAGATTAACA

4. On page 9, in line 36, the comma between "superior" and "control" should be removed.

Thank you for pointing out this grammatical issue. The comma between “superior” and “control” has been removed in the revised manuscript.

References

1. Kottur, J. & Nair, D. T. Pyrophosphate hydrolysis is an intrinsic and critical step of the DNA synthesis reaction. *Nucleic Acids Res.* **46**, 5875–5885 (2018).
2. Kohabir, K. A. V. *et al.* Synthetic mismatches enable specific CRISPR-Cas12a-based detection of genome-wide SNVs tracked by ARTEMIS. *Cell Rep. Methods* **4**, 100912 (2024).
